# *AdhesionScore*: A Prognostic Predictor of Breast Cancer Patients Based on a Cell Adhesion-Associated Gene Signature

**DOI:** 10.3390/cancers17233731

**Published:** 2025-11-21

**Authors:** Catarina Esquível, Rogério Ribeiro, Ana Sofia Ribeiro, Pedro G. Ferreira, Joana Paredes

**Affiliations:** 1 Cancer Metastasis, i3S, Instituto de Investigação e Inovação em Saúde, Universidade do Porto, 4200-135 Porto, Portugal; 2 ICBAS, Instituto de Ciências Biomédicas Abel Salazar, 4050-313 Porto, Portugal; 3 Department of Computer Science, Faculty of Sciences, University of Porto, Rua do Campo Alegre 1055, 4169-007 Porto, Portugal; 4 Laboratory of Artificial Intelligence and Decision Support, Institute for Systems and Computer Engineering, Technology and Science, Rua Dr. Roberto Frias, 4200-465 Porto, Portugal; 5 IPATIMUP, Instituto de Patologia e Imunologia Molecular da Universidade do Porto, 4200-135 Porto, Portugal; 6 FMUP, Faculdade de Medicina da Universidade do Porto, 4200-319 Porto, Portugal

**Keywords:** breast cancer, molecular signature, prognosis, cell adhesion, patient survival

## Abstract

Background: Aberrant or loss of cell adhesion drives invasion and metastasis, key hallmarks of cancer progression. In this work, we hypothesized that a gene signature related to cell adhesion could predict breast cancer prognosis. Methods: Highly variant genes were tested for association with overall survival using Cox regression. Adhesion-related genes were identified through gene ontology analysis and multivariate Cox regression, with AIC selection, defined the prognostic signature. The *AdhesionScore* was then calculated as a weighted sum of gene expression, with risk stratification assessed by Kaplan–Meier and log-rank tests. Results: We found that the *AdhesionScore* was a significant independent predictor of poor survival in three large independent datasets, as it provided a robust stratification of patient prognosis in the Molecular Taxonomy of Breast Cancer International Consortium (METABRIC) (HR: 2.65; 95% CI: 2.33–3.0, *p* = 2.34 × 10^−51^), The Cancer Genome Atlas (TCGA) (HR: 3.46; 95% CI: 2.35–5.09, *p* = 3.50 × 10^−10^), and the GSE96058 (HR: 2.83; 95% CI: 2.20–3.65, *p* = 6.29 × 10^−16^) datasets. The 5-year risk of death in the high-risk group was 32.41% for METABRIC, 27.8% for TCGA, and 17.54% for GSE96058 datasets. Consistently, HER2-enriched and triple-negative breast carcinomas (TNBC) cases showed higher *AdhesionScores* than luminal subtypes, indicating an association with aggressive tumor biology. Conclusions: We have developed, for the first time, a molecular signature based on cell adhesion, as well as an associated *AdhesionScore* that can predict patient prognosis in invasive breast cancer, with potential clinical application. We developed a novel adhesion-based molecular signature, the *AdhesionScore*, that robustly predicts prognosis in breast cancer across independent cohorts, highlighting its potential clinical utility for patient risk stratification.

## Simple Summary

Aberrant or loss of cell adhesion promotes invasion and metastasis, key features of cancer progression. In this study, we developed a gene signature based on cell adhesion called the *AdhesionScore* to predict breast cancer prognosis. We analyzed three large independent patient datasets and found that the *AdhesionScore* is an independent predictor of survival, effectively stratifying patients by risk. Aggressive subtypes, such as HER2-enriched and triple-negative breast cancers, showed higher *AdhesionScores*. This molecular signature may help identify high-risk patients and has potential to guide clinical decision-making in invasive breast cancer.

## 1. Introduction

Cell adhesion homeostasis plays a fundamental role in maintaining tissue architecture and function. Disruption in cell adhesion mechanisms are pivotal in cancer development, as the loss of cell–cell adhesion and alterations in adhesion-related signaling pathways contribute to tumor invasion and metastatic dissemination [1,2,3,4]. A key mechanism by which cancer cells acquire invasive properties is the epithelial-to-mesenchymal transition (EMT), a biological process in which epithelial cells lose their cell–cell adhesion, gain mesenchymal traits, and acquire stem-like properties [5,6]. Importantly, dysregulation of adhesion-related mechanisms in cancer does not necessarily correspond to a uniform increase or decrease in gene expression. Instead, both upregulation and downregulation of specific adhesion molecules, as well as alterations in their signaling pathways, can contribute to the loss of adhesion homeostasis that favors tumor invasion and metastatic dissemination. Therefore, transcriptional imbalance within adhesion-related pathways, rather than absolute gene expression levels, is increasingly recognized as a key determinant of breast cancer progression.

Breast cancer is the most frequently diagnosed cancer in women and exhibits significant heterogeneity at both histological and molecular levels. Traditionally, molecular subtyping based on gene expression profiles and receptor status has classified breast cancer into luminal A, luminal B, HER2-enriched, and basal-like subtypes [7]. Additional classifications, such as claudin-low and normal-like subtypes, have also been identified, further highlighting the complexity and molecular heterogeneity of this disease [8,9].

Actually, dysregulation of cell adhesion has been implicated in several breast cancer histological and molecular subtypes. Invasive lobular breast carcinoma, for instance, is characterized by mutations or loss of heterozygosity (LOH) of the *CDH1* gene, which encodes E-cadherin, a key epithelial cell–cell adhesion molecule. Although invasive lobular carcinoma is generally associated with a favorable prognosis, it can be further categorized into three molecular subtypes with distinct survival outcomes: reactive-like, immune-related, and proliferative. Among these, the reactive-like transcriptional subtype exhibits a more favorable prognosis compared to the immune-related or proliferative subtypes [10].

Claudin-low breast carcinomas, a molecular subset of ductal carcinomas, are characterized by low to absent expression of claudins and luminal differentiation markers, along with upregulated expression of EMT markers, immune response genes, and cancer stem cell-like features [11]. Clinically, claudin-low tumors are associated with poorer overall survival relative to luminal tumors, with prognostic outcomes similar to those of basal-like breast carcinomas [12,13]. Interestingly, basal-like carcinomas are also highly enriched for cell adhesion molecules, such as P-cadherin and a6b4-integrin, for example, which are crucial for stem-like properties and collective invasive behavior observed in this disease [14,15,16,17,18]. Despite identical histological parameters and treatment regimens, substantial variability in disease progression and patient outcomes persist. This underscores the critical need for novel prognostic and predictive biomarkers to refine treatment strategies, monitor disease progression, and predict therapeutic responses more accurately. Advancements in genomic and transcriptomic technologies over the past decade have elucidated the extensive molecular heterogeneity that underlies breast cancer prognosis and therapeutic response. Several commercial available molecular signatures have been developed to assess the likelihood of disease recurrence, long-term survival, and response to chemotherapy or radiotherapy, as well as to determine the potential benefits of extended adjuvant endocrine therapy [19,20,21,22,23,24,25]. However, the majority of these prognostic tools are focused on early-stage estrogen receptor (ER)-positive breast cancer and do not provide predictive insights regarding the site of distant metastasis. Additionally, some molecular signatures contribute to the classification of breast cancer subtypes and the refinement of patient staging [26]. Notably, genes associated with cellular proliferation and hormone receptor status are recurrently incorporated into various breast cancer prognostic models [26,27]. These models aim to identify the optimal gene combinations that predict disease recurrence and patient overall survival, independent of specific cellular phenotypes. Despite the availability of multiple genomic tools, no clinically available signature specifically captures adhesion-related transcriptional dysregulation, a key biological process underlying breast cancer invasion and metastatic behavior. To address this gap, we used METABRIC, TCGA, and GSE96058 datasets to build the *AdhesionScore*, a prognostic signature derived exclusively from adhesion-associated genes.

## 2. Materials and Methods

### 2.1. METABRIC Dataset

Normalized gene expression data for 2509 breast carcinomas from the Molecular Taxonomy of Breast Cancer International Consortium (METABRIC [12,13]) were retrieved from cBioPortal (http://www.cbioportal.org/ [28], accessed on 21 October 2024 ). Gene expression was profiled with Illumina HT-12 v3 microarrays (Illumina, San Diego, CA, USA), with probe-level intensity values being mean-summarized per gene. Normal breast cases were removed from the analysis and only cancer cases with complete data (expression and clinical) were considered. A summary of clinical and pathological features of this dataset is presented in Table S1A.

### 2.2. TCGA Dataset

Publicly available RNAseqV2 RSEM expression levels for 20,531 genes in 1084 breast cancer cases and the corresponding clinical data from The Cancer Genome Atlas (TCGA, http://cancergenome.nih.gov/, accessed on 12 November 2024) were downloaded from cBioPortal (http://www.cbioportal.org/ [28], accessed on 12 November 2024 ). Normal breast cases were removed from the analysis and only cancer cases with complete data (expression and clinical) were considered. Molecular subtype classification of TCGA-BRCA tumors was obtained from Perou and colleagues’ study [10]. A summary of clinical and pathological features of this dataset is presented in Table S1B. The TCGA dataset was exclusively used as a validation cohort to assess the performance of the *AdhesionScore*.

### 2.3. GSE96058 Dataset

Normalized RNA-seq expression data for 3409 breast cancer cases from the Sweden Cancerome Analysis Network—Breast (SCAN-B) initiative (GSE96058, [29,30]) were retrieved from the Gene Expression Omnibus (GEO, https://www.ncbi.nlm.nih.gov/geo/ [31], accessed on 29 September 2025). Gene expression was profiled using Illumina HiSeq 2000 and Illumina NextSeq 500 platforms (Illumina, San Diego, CA, USA). Only invasive breast cancer cases with complete clinical and molecular information were considered for analysis. Normal breast samples and technical replicates were excluded. Clinical and pathological features of this dataset are summarized in Table S1C. The GSE96058 dataset was exclusively used as an independent external validation cohort to further assess the prognostic performance of the *AdhesionScore*.

### 2.4. Survival Analysis by Cox Proportional Hazards Model

Univariate Cox proportional hazards models were developed for each gene to assess their statistical association with patients’ overall survival (OS). Highly correlated genes (correlation > 0.75) were removed prior to model selection. The coxph() function from the ‘survival’ package (version 3.8.3) in R version 4.4.1 was used to fit a model for each gene, with overall survival time (OS_MONTHS) and status (OS_STATUS) as the response variables. Hazard ratios (HR), confidence intervals (CI), and *p*-values were extracted for each gene. 

A multivariate Cox proportional hazards model was calculated using the stepAIC function from the ‘MASS’ package (version 7.3.61) in R version 4.4.1. The stepwise selection procedure was performed with both forward and backward steps based on Akaike Information Criterion (AIC), resulting in a final set of 61 genes. Only genes with adhesion-related phenotypes were included in the model creation process. Since the METABRIC dataset does not include other factors significantly implicated in breast cancer prognosis, such as histological grade and tumor size, these variables were not individually considered in the multivariate model. Model fitness was evaluated with Cox–Snell R^2^ and concordance-index (C-Index). 

LASSO-Cox regression was performed using the ‘glmnet’ package (version 4.1.9) in R version 4.4.1 to identify the most relevant adhesion-related genes associated with overall survival. The selection of the optimal lambda parameter was based on 10-fold cross-validation using the cv.glmnet function. A total of 57 genes with nonzero coefficients at the optimal lambda (lambda.min) were retained for further analysis. Model fitness was evaluated with Cox–Snell R^2^ and C-Index.

### 2.5. Survival Analysis by Kaplan–Meier

Risk assessment was conducted using the Kaplan–Meier (KM) estimator and log-rank test at 5 years (60 months), with a 95% confidence interval. The KM estimator was computed using the survfit function from the ‘survival’ package (version 3.8.3) in R, while survival curves were obtained with the ggsurvplot function from the ‘survminer’ package (version 0.5.0) in R. 

### 2.6. Gene Ontology Overrepresentation Analysis

Gene ontology overrepresentation analysis in cellular components (CC) was performed using highly variant genes (HVG) as background. The analysis was performed using the enrichGO function from the ‘clusterProfiler’ R package (version 4.12.6) [32]. Gene symbols were converted to ENTREZID identifiers using the bitr function. We focused on CC terms, with results ranked by gene ratio. The significant phenotypes related to cellular adhesion were selected.

### 2.7. Calculation of the AdhesionScore

An *AdhesionScore* was calculated for each case as the weighted sum of gene expression values, where the weights applied to the gene expressions were the corresponding beta values from the multivariate Cox proportional hazards regression with stepwise variable AIC selection. Patients were then classified into ‘Low score’ and ‘High score’ groups based on the median value of the *AdhesionScore* across all cases.

### 2.8. Unpaired Samples Non-Parametric Statistical Analyses

The median *AdhesionScores* from three or more samples (i.e., groups corresponding to breast cancer molecular subtypes) were compared with the non-parametric Kruskal–Wallis test [33].

### 2.9. Unsupervised Clustering Analysis

Unsupervised hierarchical clustering of gene expression was performed using the hclust function in R. Heatmap visualization was generated using the Heatmap function from the ‘ComplexHeatmap’ package (version 2.20.0) [34]. Clinical annotations (overall survival, molecular subtype, chemotherapy, hormone therapy, and radiation therapy) were included as heatmap annotations, with custom color schemes for each feature. Principal component analysis (PCA) was also performed to the gene expression data using the prcomp function from the ‘stats’ R package (version 4.4.1) [33].

### 2.10. Gene Expression Analysis

The dispersion analysis of the 61 adhesion-related genes, acquired using the stepAIC function, was performed using three approaches: PCA, boxplot comparison, and individual gene expression distribution. PCA was employed to evaluate whether the selected genes exhibit distinct global expression patterns compared to the background gene set. Additionally, boxplot analyses were performed to assess statistical differences in expression levels between adhesion-related genes and background genes. PCA was conducted using the prcomp function in R package, with genes grouped as either adhesion signature genes or background. The first two principal components (PC1 and PC2) were extracted and visualized to summarize the global variance structure of the dataset. PCA was applied to reduce dimensionality and identify major trends in gene expression variation. Boxplot comparisons were conducted using the ‘ggplot2’ R package (version 3.5.1) to compare the expression levels of adhesion-related genes against the background genes. The Kolmogorov–Smirnov test was applied to assess normality, and the Wilcoxon rank-sum test was used to compare gene expression levels between groups. Statistical significance was set at *p* < 0.05.

### 2.11. Receiver Operating Characteristic (ROC) Analysis

The predictive accuracy of the multivariate Cox regression model and of the *AdhesionScore* was evaluated by time-dependent ROC curve analysis. ROC curves and the area under the curve (AUC) were calculated at 5 years of overall survival using the ‘timeROC’ R package (version 0.4). Analyses were performed independently in the METABRIC, TCGA, and GSE96058 cohorts.

All statistical analyses were performed in R (version 4.4.1; R Foundation for Statistical Computing, Vienna, Austria). The following R packages were used: ‘survival’, ‘MASS’, ‘glmnet’, ‘survminer’, ‘clusterProfiler’, ‘org.Hs.eg.db’, ‘ComplexHeatmap’, ‘stats’, ‘ggplot2’, and ‘timeROC’. Figures were generated using ‘ggplot2’, ‘ComplexHeatmap’, and base R graphics.

## 3. Results

### 3.1. Cell Adhesion-Related Phenotypes Associated with the Prognostic Gene Set

**Figure 1:**
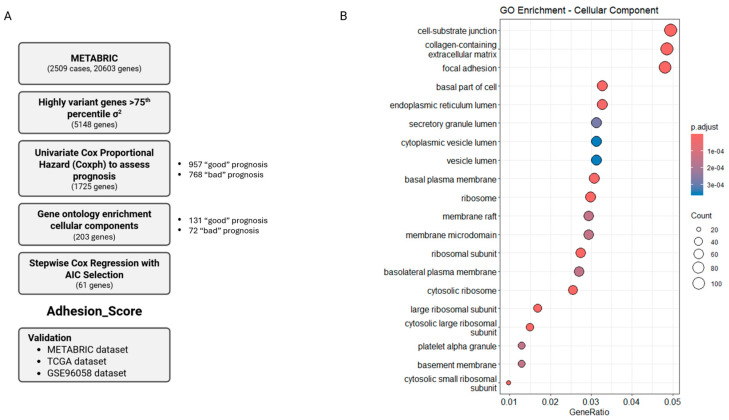
(**A**) Flow chart depicting the strategy used to derive the adhesion-related molecular signature. (**B**) Gene ontology enrichment analysis for CC terms of the 1725 survival-associated genes. CC terms were ranked according to the number of genes within each function, highlighting phenotypes related to cellular adhesion. A total of 203 genes were selected based on their association with adhesion-related phenotypes (see Figure S1 and Table S3).

A summary of the strategy used to derive an adhesion-based prognostic signature is represented in Figure 1A and is described in detail below. Transcriptomic information on invasive breast tumors available in the METABRIC dataset [12,13] was analyzed. Only cases with complete information regarding OS and transcriptional gene expression were considered, resulting in 1980 cases (out of 2509 cases). Normal breast tissue (148/1980) and unclassified tumors (6/1980) were not included in the analysis. Concerning molecular subtypes, the series comprised 700 luminal A, 487 luminal B, 223 HER2 overexpressing, 207 basal-like, and 210 claudin-low carcinomas. Considering the immunohistochemical profile of the tumors and the SNP6 affymetrix array classification, 1268 were classified as luminal, 198 as HER2 overexpressing, and 301 as triple-negative. Overall patient survival for the stratified groups matched what is expected in the clinical setting, with luminal tumors presenting the best outcome, whereas HER2 overexpressing and basal-like tumors showed the worst overall survival 5 years after diagnosis (Table S1A). Clinical and pathological characterization of the breast carcinomas showed that 69.8% (1382/1980) of the patients were more than 54 years old at diagnosis and 78.6% (1556/1980) were post-menopausal. Therapy was given to 85.6% (1695/1980) of the patients, which included hormonal therapy, chemotherapy, and/or radiotherapy (details in Table S1A). Overall survival of patients, at 5 years after diagnosis (5y OS), was 41.5% (821/1980) (Table S1A).

The variance of gene expression across all carcinoma samples (1980) was computed for 20,603 genes. Genes that had a variance above the 75th percentile were selected, yielding 5148 HVG. From these genes, univariate Cox proportional hazards model showed that 1725 genes individually had a significant prognostic impact (false discovery rate (FDR) < 0.05) (Table S2). From this prognostic gene set, 957 genes were significantly associated with ‘good prognosis’ (HR < 1) and 768 genes were associated with ‘bad prognosis’ (HR > 1) (Figure 1A). 

Enrichment of gene ontology cellular components terms was tested in the prognostic gene set. We found that several terms associated with cell–cell and cell–matrix properties were significantly overrepresented in the prognostic gene set compared with genes present in the whole genome. The top 20 significantly overrepresented terms with the highest gene ratio included 7 terms, which were associated with cell adhesion, namely ‘collagen-containing extracellular matrix’, ‘cell–substrate junction’, ‘basement membrane’, ‘focal adhesion’, ‘basal part of cell’, ‘basal plasma membrane’, and ‘basolateral plasma membrane’ (Figure 1B and Table S3). These functions comprised a total of 203 genes, of which 131 genes were associated with ‘good prognosis’ (HR < 1) and 72 genes were associated with ‘bad prognosis’ (HR > 1) in the univariate Cox proportional hazards models (Figure 1A and Figure S1). However, the list of 203 genes did not effectively distinguish molecular subtypes or survival upon 5 years of follow up (Figure S2A,B). Similarly, unsupervised hierarchical clustering revealed that these genes failed to clearly separate cases based on OS or molecular subtypes (Figure S2C).

### 3.2. Multivariate Analysis Identifies the Best Combination of Adhesion-Associated Genes for Prognosis Prediction

The findings above highlight the critical role of cell–cell adhesion in breast cancer survival, so we hypothesized that a gene signature based on the expression of adhesion-related genes could serve as a valuable prognostic tool.

Thus, we explored two distinct approaches to identify the best combination of the 203 previous mentioned genes for overall patient survival prediction. The first approach used Cox proportional hazards regression with stepwise variable AIC selection. The second approach implemented a Cox–Lasso model, which performs embedded feature selection using L1 regularization. A Cox proportional hazards model was initially fitted using gene expression data from 203 genes. Stepwise selection based on AIC was applied to identify the most relevant genes for survival prediction, resulting in a final model of 61 significant adhesion-related genes (*p* < 0.05). On the training set, this model achieved a Cox–Snell R^2^ of 0.209, a C-Index of 0.685, and an area under the ROC curve of 0.718 (Table S4 and Figure S3). To evaluate the model’s predictive performance, we calculated the ROC curve shown in Figure S3B. Additionally, we assessed the impact of reducing the number of genes by generating a model with the top 20 genes, which resulted in a lower Cox–Snell R^2^ of 0.121 and a C-Index of 0.632 (Figure S3A). 

Alternatively, a Cox–Lasso regression model was trained using the ‘glmnet’ package. This method identified 57 genes as relevant for survival prediction. The resulting model achieved a Cox–Snell R^2^ of 0.141 and a C-Index of 0.647, after 1000 model iterations (Table S5 and Figure S3A). 

Given its superior model performance, as indicated by a higher Cox–Snell R^2^ and C-Index, we proceeded with the stepwise AIC-selected model for further analyses. This approach provided a more robust prognostic signature, retaining the optimal balance between predictive power and gene set interpretability.

### 3.3. Expression and Dispersion of the 61 Adhesion-Related Genes

**Figure 2:**
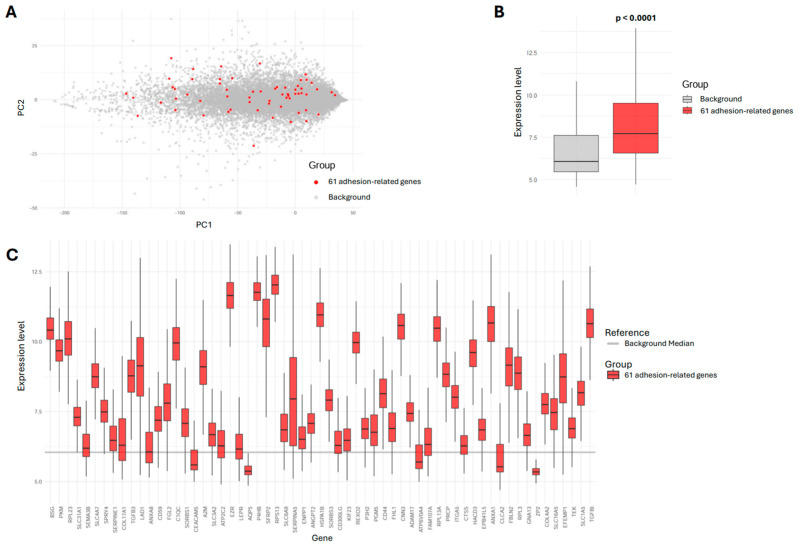
Expression and dispersion of 61 adhesion-related genes compared to background genes. (**A**) PCA of gene expression, displaying the distribution of the 61 adhesion-related genes (red) and background genes (gray) in the first two principal components. (**B**) Boxplot analysis of gene expression levels, with the 61 adhesion-related genes in red and background genes in gray. Statistical comparisons were performed using the Wilcoxon rank-sum test (*p* < 0.0001). (**C**) Individual gene expression levels for the 61 adhesion-related genes. The horizontal gray line represents the global background median expression.

To characterize the expression patterns of the 61 adhesion-related genes identified through the stepwise AIC-selected model, we conducted PCA and comparative expression analyses. The PCA plot (Figure 2A) provides a global view of gene expression variation, with PC1 (*x*-axis) representing the largest variance and PC2 (*y*-axis) capturing the second-largest independent variance. The 61 adhesion-related genes (in red) were distributed within the broader background gene set (in gray), indicating that their expression variation follows general transcriptional patterns rather than forming a distinct cluster. However, despite this broad distribution, the 61 adhesion-related genes showed significantly higher overall expression levels compared to background genes (Figure 2B). The boxplot analysis confirmed this, revealing a significantly higher median expression for adhesion-related genes (*p* < 0.0001, Wilcoxon rank-sum test). Examining individual genes showed substantial variability in expression levels (Figure 2C). While most adhesion-related genes are expressed above the background median (gray line), expression levels vary across genes, suggesting differences in regulation within this group. 

### 3.4. The AdhesionScore Is a Strong Prognostic Predictor in Breast Cancer

**Figure 3:**
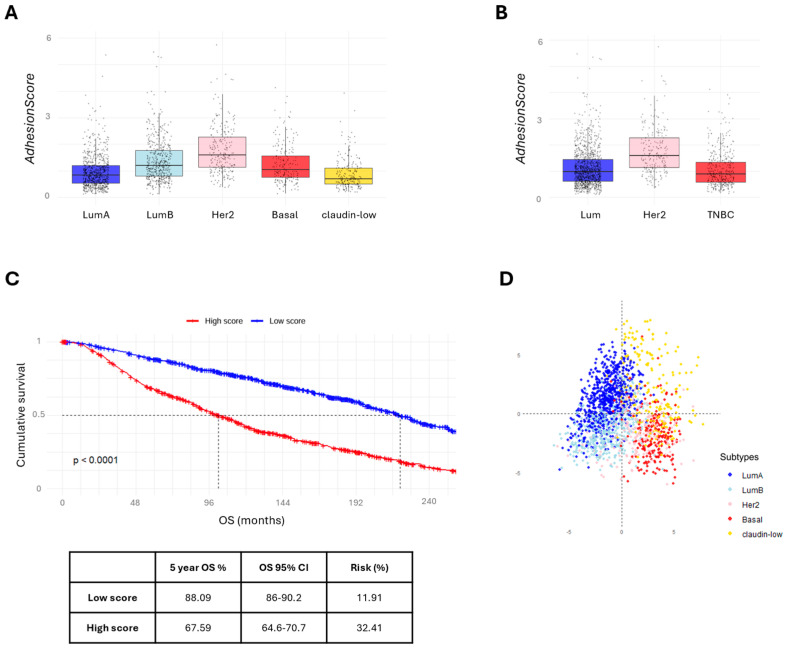
*AdhesionScore* and patient stratification in METABRIC dataset. (**A**) Distribution of the *AdhesionScore* across the five molecular subtypes of breast cancer. (**B**) Distribution of the *AdhesionScore* across the three immunohistochemistry subtypes. (**C**) Kaplan–Meier survival curves for breast cancer patients stratified by high and low *AdhesionScore*. (**D**) PCA for the 61 adhesion-related genes showing patient stratification according to molecular subtypes. LumA: luminal A; LumB: luminal B; TNBC: triple-negative breast cancer.

**Figure 4:**
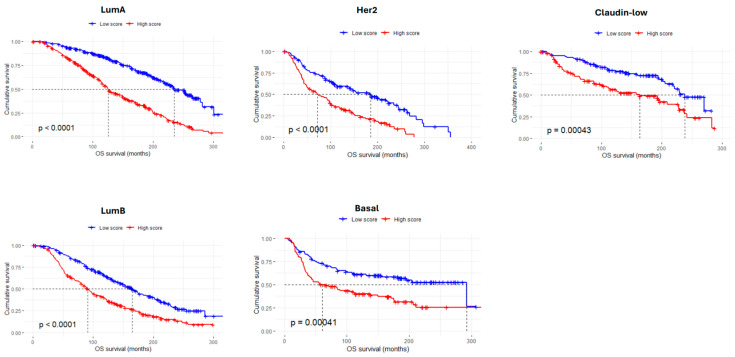
Kaplan–Meier plots showing survival of breast cancer patients according to the *AdhesionScore*, stratified by molecular subtype. The *AdhesionScore* is a significant predictor of survival across all subtypes in the METABRIC dataset. LumA: luminal A; LumB: luminal B.

Using the 61 adhesion-related genes, we developed an *AdhesionScore*, which was applied to the entire METABRIC dataset. The *AdhesionScore* was found to be significantly higher in HER2-overexpressing carcinomas (*p* < 0.05, Kruskal–Wallis tests) (Figure 3A,B). Kaplan–Meier analysis showed that the 5-year overall survival for patients in the ‘low score’ group was 88.09% (CI: 86–90.2), corresponding to a death risk of 11.91% (Figure 3C). The predictive performance of the *AdhesionScore* was further evaluated using ROC curves, yielding an area under the curve (AUC) of 0.674 (Figure S4A). In addition, it was very interesting to observe that the *AdhesionScore* clearly separates molecular subtypes by PCA (Figure 3D) and can stratify patients across all breast cancer subtypes (Figure 4). 

### 3.5. Validation of the AdhesionScore in Two Independent Breast Cancer Datasets

After applying the *AdhesionScore* to the METABRIC dataset, we validated the same signature in two independent breast cancer cohorts: TCGA-BRCA and GSE96058. The TCGA-BRCA cohort includes gene expression data from 1084 cases with comprehensive clinical and survival information, while GSE96058 comprises 3409 breast cancer cases with similar molecular and clinical annotations.

**Figure 5:**
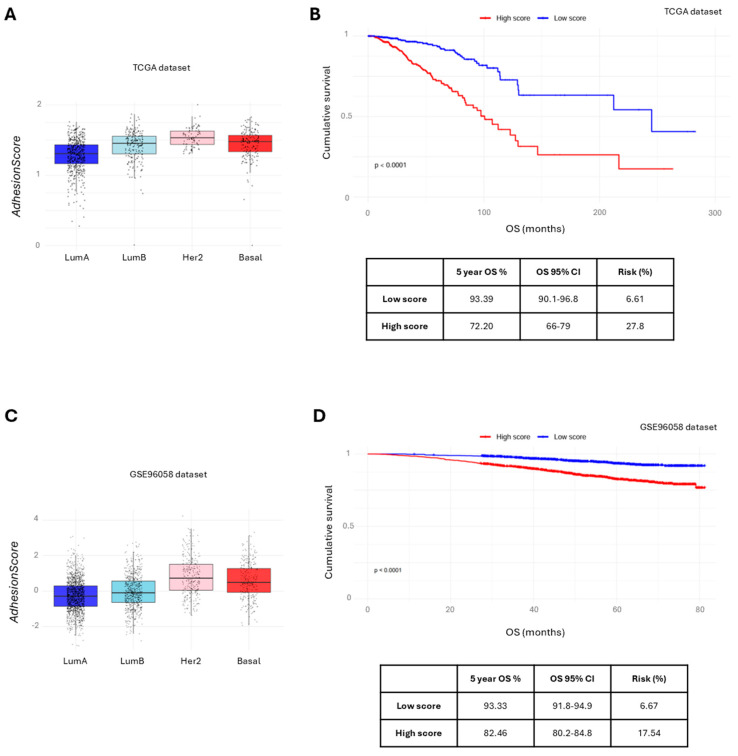
Validation of the *AdhesionScore* in independent breast cancer cohorts. (**A**) Boxplot showing the distribution of the *AdhesionScore* across molecular subtypes in the TCGA dataset (**B**) Kaplan–Meier survival curves of TCGA patients stratified by high and low *AdhesionScore*, with significance assessed using the Cox proportional hazards model. (**C**) Boxplot showing the distribution of the *AdhesionScore* across molecular subtypes in the GSE96058 dataset. (**D**) Kaplan–Meier survival curves of GSE96058 patients stratified by high and low *AdhesionScore*, with significance assessed using the Cox proportional hazards model. LumA: luminal A; LumB: luminal B.

Although the survival data of TCGA-BRCA is considered immature in some studies [35], the *AdhesionScore* was also significantly higher in HER2-enriched breast carcinomas (*p* < 0.05, Kruskal–Wallis, Figure 5A) and effectively stratified patients by overall survival, with a 5-year survival rate of 93.39% in the low-risk group (CI: 90.1–96.8%) and an estimated risk of death of 6.61% (log-rank test, *p* = 0.011, Figure 5B). The predictive performance of the *AdhesionScore* was further evaluated using ROC curves, yielding an area under the curve (AUC) of 0.617 for TCGA (Figure S4B), indicating a moderate ability to discriminate patients with different survival outcomes. Similarly, in the GSE96058 cohort, the *AdhesionScore* distinguished HER2 tumors (*p* < 0.05, Figure 5C) and stratified OS in accordance with risk groups, with a 5-year survival rate of 93.33% in the low-risk group (CI: 91.8–94.9%) corresponding to a death risk of 6.67% (Figure 5D). The AUC was 0.659 (Figure S4C), demonstrating once again a moderate predictive performance and confirming the reproducibility of the signature across independent datasets.

**Figure 6:**
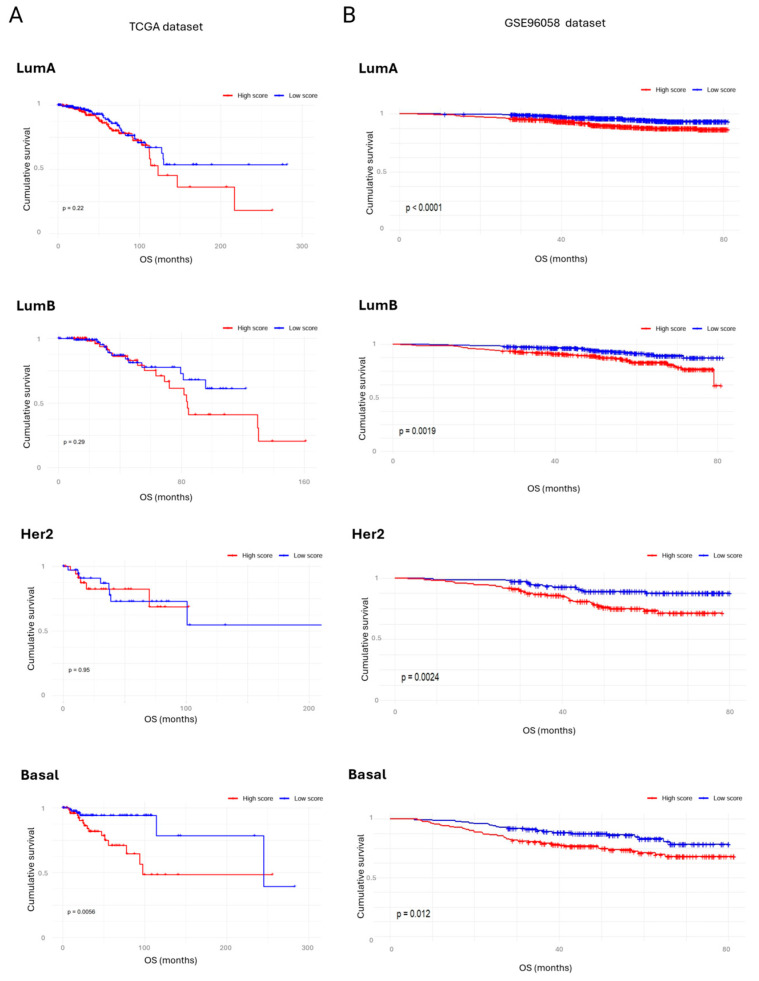
*AdhesionScore* and survival stratification by molecular subtype. (**A**) Kaplan–Meier survival curves of TCGA patients stratified by molecular subtypes, showing prognostic value of the *AdhesionScore* within each subtype. (**B**) Kaplan–Meier survival curves of GSE96058 patients stratified by molecular subtypes, confirming the predictive performance of the *AdhesionScore* across independent cohorts. LumA: luminal A; LumB: luminal B.

Further stratification by molecular subtypes revealed cohort-specific differences in the prognostic value of the *AdhesionScore*. In the TCGA dataset, a significant survival association was observed only for basal-like tumors (*p* = 0.0056), whereas luminal and HER2-enriched subtypes did not reach statistical significance (Figure 6A). In contrast, in the GSE96058 cohort, consistent with the METABRIC results, high *AdhesionScore* values were significantly associated with poorer survival across all molecular subtypes (Figure 6B). These findings confirm the robustness of the *AdhesionScore* as a predictor of aggressive disease, particularly in GSE96058 and METABRIC, supporting its potential clinical utility across multiple independent breast cancer cohorts.

## 4. Discussion

Cell adhesion molecules play a fundamental role throughout all stages of cancer progression [36]. Indeed, previous studies have demonstrated the relevance of adhesion molecules in breast cancer progression and patient outcomes [1,2,3,4]. In this study, 61 adhesion-related genes were identified and an *AdhesionScore* that can help predict patient prognosis was generated. Although the PCA did not reveal significant dispersion, collective expression of these genes was notably high. Key genes, such as *BSG*, *TGFBI*, and *ADAM17*, were already shown as having well-established roles in breast cancer biology. *BSG* promotes matrix metalloproteinase activation and ECM degradation, facilitating invasion and metastasis [37]. *TGFBI* is a secreted ECM protein regulated by TGF-β that modulates integrin signaling and metastatic potential [38,39]. *ADAM17* is a key molecule that regulates EGFR ligand activation and inflammatory signaling, contributing to tumor–stromal communication [40,41]. Other signature genes, including *ITGA5*, *ANGPT2*, and *CD44*, have recognized roles in cancer progression and are already under investigation as therapeutic targets [42,43,44]. 

Clinically, the *AdhesionScore* may facilitate early disease detection, risk stratification, and identification of patients who could benefit from future adhesion-targeted interventions. Several molecules, such as integrin inhibitors, anti-CD44 agents, ANGPT2-targeting compounds, and ECM modulators, are in clinical or preclinical evaluation [42,45,46,47,48,49], illustrating how modulation of adhesion pathways through integrin signaling, mechanotransduction, and stromal activation may influence therapeutic vulnerability. Although not yet in clinical practice, these strategies suggest that patients with high *AdhesionScores* could potentially benefit from adhesion-focused therapies, alone or in combination with conventional treatments.

The 5-year risk of death for patients in the ‘Low Score’ subgroup was 11.91% in METABRIC, consistent with the general cut-off of 10% used in commercial molecular signatures for guiding treatment decisions. A major limitation of new molecular signatures is their reproducibility in independent datasets and their insufficient sensitivity or specificity to meet clinical needs [50]. In this study, the *AdhesionScore* was validated not only in TCGA-BRCA, but also in the independent GSE96058 cohort, replicating the prognostic performance observed in METABRIC. ROC analysis demonstrated moderate, but consistent, predictive performance, with AUCs of 0.674, 0.617, and 0.659 in METABRIC, TCGA, and GSE96058, respectively. Stratification by molecular subtypes revealed cohort-specific differences: in TCGA, only basal-like tumors showed significant survival differences, whereas in GSE96058, the *AdhesionScore* robustly predicted poor survival across all molecular subtypes, mirroring the results obtained with the METABRIC datsaset. These findings confirm the robustness and generalizability of the *AdhesionScore*.

Overall, the signature preserved prognostic performance across intrinsic subtypes, although some cohort-specific differences were observed, likely reflecting clinical and biological heterogeneity. Importantly, differences in statistical significance across the three cohorts may also arise from clinical and treatment-related variability that is not captured in the available datasets. Postoperative therapies differ between METABRIC, TCGA, and GSE96058, and treatment information is incomplete or inconsistently reported, preventing adjustment for potential therapy-induced effects on adhesion-related transcriptional programs. As a result, it remains unclear whether differences in treatment exposure could contribute to the observed variation in survival significance between low- and high-score groups. Moreover, differences in prognostic performance across molecular subtypes can also be explained by subtype-specific adhesion biology. Basal-like tumors show enrichment in P-cadherin, ITGA6/ITGB4, and CD44 [16,17,51]; claudin-low tumors show downregulation of tight junction proteins and high EMT-related adhesion gene expression [11]; luminal tumors tend to maintain epithelial adhesion markers [52]. This biological context helps explain why the *AdhesionScore* performs slightly differently across cohorts and subtypes.

When compared with commercial breast cancer molecular signatures, such as Oncotype DX^®^, MammaPrint^®^, Prosigna^®^ (PAM50), EndoPredict^®^, Breast Cancer Index^®^, Mammostrat^®^, and IHC4, the *AdhesionScore* is biologically distinct. Most commercial assays primarily assess cell proliferation, hormone receptor signaling, and endocrine response pathways, whereas the *AdhesionScore* exclusively captures adhesion- and ECM-related transcriptional states [19,20,21,22,23,24,25,26,27,53]. This biological difference likely explains the slightly lower AUC observed here (0.617–0.674) compared with reported AUCs of approximately 0.68–0.78 for recurrence prediction in large validation cohorts of Oncotype DX^®^ and MammaPrint^®^ [54]. However, these commercial models are not directly comparable due to differences in cohort composition, clinical endpoints, and proprietary algorithms. Importantly, adhesion-based signatures, such as the *AdhesionScore,* may provide complementary prognostic information, particularly in patients with intermediate or indeterminate risk classification in existing commercial tests. Integrating adhesion-related biology with proliferation and endocrine-response markers could enhance multigene prognostic models in future studies.

Although the current signature does not provide guidance for therapy modification, it can identify patients with poor prognosis who may benefit from more frequent monitoring, more aggressive treatment, or therapies targeting ECM remodeling, integrins, or stromal activation. Future studies integrating genetic and epigenetic alterations of adhesion-related genes may further enhance predictive power. Molecular signatures based on cell adhesion have shown prognostic value in other cancers, including ovarian, osteosarcoma, hepatocellular, gastric cancers, and multiple myeloma [55,56,57,58,59], highlighting the translational relevance of this biological pathway.

In conclusion, the *AdhesionScore* represents a biologically grounded, prognostically informative signature. Its validation across METABRIC, TCGA, and GSE96058 cohorts demonstrates its potential clinical utility, offering a tool for personalized risk stratification in invasive breast cancer.

## 5. Conclusions

In this study, a transcriptome-wide approach was employed to identify genes involved in cell adhesion with implications on breast cancer patients’ outcomes. Through this methodology, we established a novel prognostic molecular signature for invasive breast cancer. Our proposed 61-gene signature may aid in identifying patients at higher risk of poor treatment response and could provide insights into disease prognosis. However, further studies are required to validate its clinical applicability.

## Data Availability

The datasets analyzed during the current study are available from the corresponding author upon reasonable request. All data generated during this study are included in this published article and its Supplementary Information Files. The code used and generated in this study is available on GitHub at the following link: https://github.com/CatarinaEsquivel/AdhesionScore.git (accessed on 12 November 2024).

## Supplementary Materials

The following supporting information can be downloaded at https://www.mdpi.com/article/10.3390/cancers17233731/s1. Figure S1: List of the cellular adhesion genes with impact in prognosis (203 genes—72 genes ‘bad’ prognosis and 131 genes ‘good’ prognosis). Figure S2: (A) Unsupervised machine learning clustering showing that the 203 adhesion genes did not allow stratification of patients according to molecular subtypes (B) or 5-year overall survival (PCA). (C) Unsupervised hierarchical clustering. Figure S3: (A) R square and Concordance index were calculated for both models consisting of random selection of 61 and 57 genes, respectively, from the 203 gene list. (B) ROC curve for the Cox regression model with AIC selection. (C) The values obtained with Cox regression model with AIC selection model are shown with horizontal lines. Figure S4: Predictive performance of the *AdhesionScore* assessed by ROC curves in independent breast cancer cohorts. (A) ROC curve of the *AdhesionScore* in the METABRIC dataset, showing moderate discrimination of patients with different overall survival outcomes (AUC = 0.674). (B) ROC curve of the *AdhesionScore* in the TCGA dataset, indicating moderate predictive performance (AUC = 0.617). (C) ROC curve of the *AdhesionScore* in the GSE96058 cohort, confirming reproducibility and moderate discrimination of survival outcomes (AUC = 0.659). Table S1: Clinical and pathological features of the breast series METABRIC (A), TCGA-BRCA (B), and GSE96058 (C). Table S2: Highly variant genes with impact in prognosis constitute. HR > 1 indicates ‘bad’ prognosis genes; HR < 1 indicates ‘good’ prognosis genes. FDR cutoff of 0.05. Table S3: Gene ontology CC analysis of highly variant genes with impact in prognosis. Table S4: List of genes present in the Cox regression model with AIC selection. Table S5: List of genes present in the Lasso-penalized Cox Regression.

## Abbreviations

The following abbreviations are used in this manuscript:

AIC	Akaike Information Criterion
AUC	Area Under the Curve
BRCA	Breast Invasive Carcinoma
CC	Cellular Components
C-Index	Concordance-Index
CI	CI Confidence Interval
*Coxph*	Cox Proportional Hazards
EMT	Epithelial-to-Mesenchymal Transition
ER	Estrogen Receptor
FDR	False Discovery Rate
GEO	Gene Expression Omnibus
GO	Gene Ontology
HR	Hazard Ratios
HVG	Highly Variant Genes
KM	Kaplan–Meier
LOH	Loss of Heterozygosity
METABRIC	Molecular Taxonomy of Breast Cancer International Consortium
OS	Overall Survival
PC	Principal Components
PCA	Principal Component Analysis
ROC	Receiver Operating Characteristic
TCGA	The Cancer Genome Atlas
TNBC	Triple-Negative Breast Carcinomas
