# Supplementary material for: AdhesionScore: A Prognostic Predictor of Breast Cancer Patients Based on a Cell Adhesion-Associated Gene Signature
Source: Cancers (Basel). 2025 Nov 21;17(23):3731. doi: 10.3390/cancers17233731 (PMC12691146; doi:10.3390/cancers17233731)
Supplement: Supplementary file 1 [file cancers-17-03731-s001.zip › SuppTable1.pdf]

A

		No. of Patients	Percentage
Age	< 54 years	1382	69,7979798
	≥ 54 years	598	30,2020202
Menopausal state	post	1556	78,58585859
	pre	424	21,41414141
Laterality	left	963	48,63636364
	right	884	44,64646465
	NA	133	6,717171717
Cellularity	high	980	49,49494949
	moderate	736	37,17171717
	low	205	10,35353535
	NA	59	2,97979798
Histological subtype	Invasive Ductal Carcinoma	1551	78,33333333
	Mixed Ductal and Lobular Carcinoma	207	10,45454545
	Invasive Lobular Carcinoma	151	7,626262626
	Invasive Breast Carcinoma - NOS	32	1,616161616
	Mixed Mucinous Carcinoma	21	1,060606061
	Metaplastic Carcinoma	18	0,909090909
Nottingham Prognostic Index	<1= 2.4	161	8,131313131
	> 2.4 to ≤ 3.4	485	24,49494949
	> 3.4 to ≤ 5.4	1129	57,02020202
	> 5.4	205	10,35353535
ER (IHC)	neg	465	23,48484848
	pos	1515	76,51515152
HER2 (array)	gain	446	22,52525253
	loss	103	5,202020202
	neut	1426	72,02020202
	undefined	5	0,252525253
Subtypes	Luminal A	700	35,35353535
	Luminal B	487	24,5959596
	Her2	223	11,26262626
	Claudin-low	210	10,60606061
	Basal	207	10,45454545
	Normal	147	7,424242424
	Not Classified	6	0,303030303
IHC–SNP6 Subtype	Luminal	1268	64,04040404
	HER2	198	10
	Triple Negative	301	15,2020202
	NA	213	10,75757576
Therapy (any)	no	285	14,39393939
	yes	1695	85,60606061
Hormone therapy	no	737	37,22222222
	yes	1243	62,77777778
Chemotherapy	no	1561	78,83838384
	yes	419	21,16161616
Radiotherapy	no	794	40,1010101
	yes	1186	59,8989899
5 year overall survival	alive	821	41,46464646
	deceased	1159	58,53535354

B

		No. of Patients	Percentage
AJCC pT	T1 +T1a+T1b +T1c	189	25,96153846
	T2 + T2a + T2b	432	59,34065934
	T3 + T3a	76	10,43956044
	T4 + T4b +T4d	28	3,846153846
	TX	3	0,412087912
AJCC pN	N0 + N0 (i-) + N0 (i+) + N0 (mol+)	356	48,9010989
	N1 +N1a+N1b+N1c+N1mi	245	33,65384615
	N2 + N2a	87	11,95054945
	N3 + N3a + N3b + N3c	40	5,494505495
AJCC pM	MO + cM0 (i+)	711	97,66483516
	M1	12	1,648351648
	NA	5	0,686813187
AJCC pStage	I	115	15,7967033
	II A	221	30,35714286
	II B	108	14,83516484
	III A	75	10,3021978
	III B	9	1,236263736
	III C	28	3,846153846
	IV	4	0,549450549
	No_Conversion	168	23,07692308
ER (IHC)	neg	166	22,8021978
	pos	562	77,1978022
PR (IHC)	neg	234	32,14285714
	pos	494	67,85714286
HER2 (IHC+FISH)	neg	618	84,89010989
	pos	110	15,10989011
Subtype	Luminal A	219	30,08241758
	Luminal B	122	16,75824176
	HER2+	53	7,28021978
	Basal-like	92	12,63736264
	Normal-like	8	1,098901099
	NA	234	32,14285714
5 year overall survival	alive	654	89,83516484
	deceased	74	10,16483516

C

		No. of Patients	Percentage
Age	< 50 years	609	18,60678277
	≥ 50 years	2664	81,39321723
Positive lymph nodes (number)	0	2013	61,50320807
	1-3	869	26,55056523
	≥ 4	298	9,104796822
	Missing	93	2,841429881
Nottingham Histological Grade	Grade 1	496	15,1542927
	Grade 2	1532	46,80721051
	Grade 3	1184	36,17476321
	Missing	61	1,863733578
ER status	neg	241	7,363275283
	pos	2832	86,52612282
	Missing	200	6,110601894
PgR status	neg	386	11,79346166
	pos	2554	78,03238619
	Missing	333	10,17415215
HER2 status	neg	2731	83,44026887
	pos	420	12,83226398
	Missing	122	3,727467156
Ki67 status	low	907	27,71157959
	high	643	19,64558509
	Missing	1723	52,64283532
PAM50 Subtype	Luminal A	1657	50,62633669
	Luminal B	729	22,2731439
	Her2	327	9,990834097
	Basal	339	10,35747021
	Normal	221	6,752215093
