# Supplementary material for: AdhesionScore: A Prognostic Predictor of Breast Cancer Patients Based on a Cell Adhesion-Associated Gene Signature
Source: Cancers (Basel). 2025 Nov 21;17(23):3731. doi: 10.3390/cancers17233731 (PMC12691146; doi:10.3390/cancers17233731)
Supplement: Supplementary file 1 [file cancers-17-03731-s001.zip › SuppTable2.pdf]

Gene	HR	Coef	p_value	FDR
CIDEA	0.898122878369016	-0.107448384440833	0.000170332513653239	0.00137520811571761
SDS	1.19727414195003	0.180047424539291	0.000690711069177276	0.00418385609021982
HIPK2	0.869536001727115	-0.139795540953403	0.000573672705253533	0.0035774674391779
SHMT2	1.31671429414968	0.27513946236261	1.74543392601633e-07	7.11223132550011e-06
TM2D1	0.887746607532045	-0.119068928588888	0.0119796158747455	0.0383750008524964
RASD2	1.08251674828243	0.0792886526292613	0.00990672203291054	0.0333308459774802
ITGA10	0.790237879669909	-0.235421265340429	3.59308498806793e-05	0.000416846413818422
RPS24	1.11627599804051	0.109998143455166	0.00801429673801013	0.0282091197087301
GLT8D2	0.88680666037282	-0.120128290698925	0.000185003879620664	0.00148204507609026
ECI2	0.877292944857656	-0.130914311761298	0.000362856516300511	0.00250882404760259
ANTXR2	0.851712732460865	-0.160505977480054	0.00082251199394181	0.0048090343709356
MIEN1	1.19120934959933	0.174969051248174	1.31532485420931e-05	0.000195251824900063
PDXDC2P-NPIPB14P	1.10701419550746	0.101666477047585	0.00581349680860747	0.022106440238595
KRT8	1.12630254949116	0.118940187667607	8.08841877310634e-05	0.000753848877293937
CD52	0.921478052368734	-0.0817763194443007	0.00023955111952234	0.00181727218948391
ARPC5	0.854279482840158	-0.157496875453641	0.00236632890856803	0.0109909469865049
ZNF385A	1.14246184161863	0.133185444207568	0.0119006083564208	0.0381931673793917
SEPTIN11	0.849222108638411	-0.16343451482588	9.37653461117883e-05	0.000844379891296891
CEBPB	1.15008241226838	0.13983360264957	0.000538444286256846	0.003415673052351
CLDN3	1.0849593178733	0.0815424912418157	0.00785773444006075	0.0277607613859759
SIX4	1.21400890481385	0.193928027712426	7.70193156273256e-08	3.85171354171218e-06
PSME4	1.17226331082164	0.158936333857314	0.000558846147971844	0.00350623204409619
BSG	1.19431092349259	0.177569386006102	0.000243941638558701	0.0018370517254618
ACKR1	0.871642279043991	-0.137376169694829	1.22161145977509e-08	9.50717762694417e-07
TAT	0.915720056447376	-0.0880445762499811	1.48730855431964e-05	0.000213401848559902
PIGR	0.936877211889936	-0.0652030492048483	0.0144982982054631	0.0448532937275317
LRATD2	1.17928130351179	0.164905188101728	2.79361667273375e-05	0.000352694104932635
CPVL	0.920120899319676	-0.083250205268896	0.00642542701319848	0.0237496686324984
BOLA2B	1.13620973855793	0.12769793226938	0.0123022106055347	0.0392132963051417
NDN	0.877736606135713	-0.130408723365642	0.00019519091680742	0.00155245567550324
PSMD3	1.277696895337	0.245059156728019	1.43246761947193e-09	2.17018844349997e-07
GLRB	0.905966844099466	-0.0987525695255091	0.00128321606555232	0.00680025303874486
GLA	0.8092665313156	-0.21162695843806	1.31449136690747e-09	2.11592032216888e-07
TDG	1.29651203698201	0.25967761016637	2.03794363542797e-08	1.41857400893101e-06
LY96	0.896790239980316	-0.108933290445412	0.00576606993789473	0.0219682146820235
MIR99AHG	0.811670948674432	-0.208660256573224	4.87979064576199e-07	1.43286649214431e-05
BBOF1	0.892270178522544	-0.113986301543921	0.00380377075007799	0.0160089640785735
FGFR4	1.11486724570757	0.108735335677946	8.49415819366116e-05	0.000784111269812699
IQCG	0.867070668551921	-0.14263479623619	0.00487879171616369	0.0193461556042796
GAS6	0.854839986692231	-0.156840977627702	3.60347324822243e-05	0.000417112150597612
CGNL1	0.903609945043772	-0.101357488424317	0.000364318369902366	0.00251555485706044
CEMIP	1.09815829301425	0.0936344975547357	0.00143296500656645	0.00743323539659996
RDH5	0.814027128682889	-0.205761585914693	0.000130798449815885	0.00111362448760599

RAC2	0.917732221264909	-0.0858496288822831	0.00799227038925696	0.0281588131156379
TERF1	1.16393342267039	0.151805150654867	0.00281199986361839	0.0126282574520473
ARHGAP44	1.13856918841986	0.12977237623409	0.00838758905887243	0.0291922102988188
S100A11	1.12436722343536	0.117220409354429	0.0107972166564887	0.0356287399087595
SLC16A3	1.19305315821368	0.17651570055869	1.05375576948288e-07	4.9344508805512e-06
CDCA5	1.21132568986483	0.191715371322973	1.61120476902394e-10	5.53287717682822e-08
ALKAL2	0.869515402978772	-0.13981923059051	0.00576117817032701	0.0219658243933045
OLFML1	0.81763682404485	-0.201337021353132	0.000160856637939699	0.00131519451115459
CTTN	1.16257583504454	0.15063809079862	0.000607876183126531	0.00375890782627222
PPFIA1	1.12360222437812	0.1165397959346	0.00750380318777869	0.0267303528494108
RNASE6	0.896110275047014	-0.109691798751035	0.00626349949748787	0.0233622635130775
YPEL3	0.85617090250305	-0.15528527031146	0.00433387000989153	0.0176278645957003
IRF8	0.910949739625694	-0.0932675537977038	0.00406636188173564	0.0168374839652896
AUNIP	1.22136649271557	0.199970307924425	4.61026834975831e-05	0.000503777481275317
MMP10	0.891666940832526	-0.114662600844457	0.00896919734218299	0.0308413454670124
FABP4	0.92823196303689	-0.0744736172617119	0.000115717101102505	0.00100515815814335
EPB41L4A-AS1	0.866653975072736	-0.143115487894743	0.00232659475044546	0.0108259164946202
FAM83D	1.22950998130877	0.206615700826413	3.32164931450212e-08	2.03688281178576e-06
UGP2	0.898114605473247	-0.107457595801629	0.0133689544328085	0.0418877641626501
RPL36	0.824506696367378	-0.19297001528426	0.000555452953141298	0.00349772391397412
TPT1	0.861095397002362	-0.149549982779891	1.8703229583967e-06	4.21884363929114e-05
MELK	1.16500453307903	0.152724978065059	9.46115911017215e-07	2.46133487760085e-05
PKM	1.27717919492882	0.244653892130463	1.09320253854294e-06	2.77393412612547e-05
	1.17994960880389	0.165471733162231	0.00071450972928391	0.00428955666147019
TUBA3C	0.7681017931765	-0.263833011418971	1.3761575225555e-05	0.000200941515884006
AQP9	1.10645013071702	0.101156810100654	0.00355843121752606	0.0151608595545713
GRP	0.944406464794774	-0.0571986283952525	0.0051343259239239	0.020106535338485
RPL7A	0.821576535753454	-0.196530179985136	1.79389965610492e-05	0.000246410056762571
AGTR1	0.943630864185083	-0.0580202230124939	0.00400387116266144	0.0166322099668299
PHGDH	1.06407693968981	0.0621077000436239	0.00614398426784376	0.0230836345468003
DLGAP5	1.21629147339159	0.195806453333695	6.42568064275001e-05	0.000652151116730121
PPM1F	0.874972857282152	-0.133562413354629	0.00906208071157379	0.0311057056726251
ANXA3	0.873395374492961	-0.135366933922533	0.00638810312157093	0.0236557291007993
LRP11	1.17077144752244	0.157662888386225	0.00296228664075821	0.0130863966436926
GUCY1B2	1.22946764648743	0.206581267962228	1.72473801788054e-05	0.000238820578766201
SLC7A5	1.07825975281935	0.0753484015495214	0.000637763738550492	0.00391551968685767
GLYATL2	1.07895870495951	0.0759964139583701	3.88046478537277e-05	0.000440270354833813
CENPX	1.16001145817032	0.14842988280252	0.00273827839577701	0.0123510262842797
IL33	0.800546068141311	-0.222461198992877	1.66473742857977e-07	6.91537297952776e-06
KERA	0.715079586047529	-0.335361433325151	4.34657636866965e-07	1.31572096358616e-05
SOGA1	1.15370749891414	0.142980668812221	0.00213861900641363	0.0101250243584895
TNMD	0.770565708245658	-0.260630347850756	8.09315529302169e-05	0.000753848877293937
AGO2	1.1801880914368	0.16567382529753	2.84570682615106e-07	9.54660413084459e-06
DNASE1L3	0.702396272890195	-0.353257542925031	2.01099533245856e-08	1.41857400893101e-06

GAS1	0.905985305741108	-0.0987321918994837	0.00026627066298377	0.0019678051435142
KIT	0.851636599327798	-0.160595369751735	9.51793596405482e-08	4.62517812743834e-06
INSIG1	1.150200170205	0.139935988276119	0.000234988031742862	0.00179321978001109
AMPH	0.841847334166437	-0.172156594520585	6.39386925455676e-06	0.000110666406655553
SLC25A19	1.17907894436689	0.164733578064142	0.00149800014227678	0.00770079713859051
CFH	0.848685955240264	-0.16406606069974	1.1267292183431e-05	0.000171203014857974
RPL23	0.887873287448321	-0.118926240486723	0.000120201279468448	0.00104235149922891
CCL14	0.877465197706826	-0.130717985161586	9.08181225426797e-07	2.37464035135707e-05
SLC31A1	1.16914424561211	0.156272067189199	0.00357941029835588	0.0152098630795213
SRSF6	1.1419407456883	0.132729223450108	0.00507510140223077	0.0199284638499292
ACADM	0.889307874794025	-0.11731178756793	0.00646378261412309	0.0238673435450524
SEMA3B	0.828052691982404	-0.18867848896176	3.897223691569e-05	0.000441199983192789
NOL3	1.24325455901092	0.217732585616577	1.0083476797998e-05	0.0001564457499593
H2BC8	1.1079044126974	0.102470314474909	0.000681765814376316	0.00415103511802885
RBM14	1.09996956247353	0.0952825089428771	0.0117773507272235	0.0379394206353523
SOX10	0.886758852317221	-0.12018220250006	8.46400414171695e-05	0.000783343291603712
SEPTIN7	0.822175626750608	-0.19580124890009	0.0012727428094468	0.00677410944289039
IGLL3P	0.921637741342511	-0.0816030379014688	0.000603005328044682	0.00373326976533432
TSPAN7	0.852800799835425	-0.159229287677675	5.66909115613716e-06	0.000101043212959386
ROPN1B	0.917273303739216	-0.0863498099760257	0.00358839501812573	0.0152255541502188
RPS17	0.827967508602538	-0.18878136618501	7.29419315757972e-06	0.000121593491762761
TBX1	1.23260076066372	0.209126376667121	4.64166459243213e-05	0.00050441380412696
WT1-AS	1.15027823632788	0.140003857744219	0.00571271837375398	0.0218632155019871
FOXD2	1.16931763343187	0.156420359382139	1.66858007864136e-05	0.000233555869159827
ZIC2	1.17297255601697	0.159541172992666	1.26141663206319e-07	5.55346758269869e-06
ALDH1A2	0.747134483291213	-0.291510078873319	6.78681795993555e-08	3.5312019506695e-06
TENT4B	1.14411501793121	0.134631428053439	0.0043353157721572	0.0176278645957003
FCGR1A	1.13934334323329	0.130452081738559	0.0133232910240241	0.0417955371892498
TAOK2	1.15601660603384	0.144980135228186	0.00270950012697477	0.0122534110219904
CLK1	0.827180897846199	-0.189731868040306	6.67834622185457e-05	0.000667964298811124
P2RY13	0.769738949080179	-0.261703848785709	2.11357106029472e-07	7.66690459970287e-06
GAB2	0.860876590059487	-0.149804118091842	0.00158980698117908	0.00802082718912673
RPL36.1	0.808384221172458	-0.212717812232402	3.82973690443536e-05	0.000435474057279173
NMRAL1	1.14242765894559	0.133155523571995	0.0122841759064189	0.039180055785736
RNF125	0.764405607269979	-0.268656731124897	1.39625059401969e-06	3.35692743966551e-05
RAI2	0.838623628618397	-0.175993268374357	0.000535823828933979	0.00340744264548016
LAX1	0.881307519643481	-0.12634865646826	0.00982443939951033	0.0331405942022775
IGHG1	0.964563474312624	-0.0360796382132591	0.0159420942424733	0.0480465999894803
ADCY1	0.936423062060153	-0.0656879152404705	0.00426784250487494	0.0174612047201039
CCR2	0.795419123522107	-0.228886103849708	9.71191981499497e-06	0.00015205501205787
HIST1H4J	1.08761295450426	0.0839853447977989	0.0145414021778948	0.0449596414275727
HES6	1.1101977449011	0.104538148017222	0.000953715004897324	0.00535140086081276
ANKRD30B	1.07433185827016	0.0716989411609809	0.0123630123354383	0.0393337032175497
ELL2	1.14772802978596	0.137784361978326	0.0115349639785726	0.0374627209126635

STARD3	1.19429917144074	0.177559545930586	2.62539963356501e-06	5.59976766770388e-05
C11orf1	0.811085372970341	-0.209381961639717	1.40116365662606e-06	3.35692743966551e-05
CHI3L2	0.912894224453663	-0.091135260023663	0.000279935169653612	0.00205113237394844
FAM222A	1.23009633819036	0.20709249004918	0.000124016450582629	0.00106743650609022
RNASEH2B	0.854915188766414	-0.156753009390716	0.00318611643499978	0.0138378463378447
TNFRSF13B	0.759808287087189	-0.274689131355756	2.95737458228493e-06	6.14251470699584e-05
PBX1	1.24555059453921	0.219577676765153	1.91000200941162e-07	7.31253667275701e-06
SLC4A7	0.853833250562499	-0.158019361211265	0.000879653520897282	0.00505138827886499
STIP1	1.47849980675495	0.391027929606935	5.06001113211713e-13	8.68803911384511e-10
UCK2	1.1804237723589	0.165873503123855	0.000715790906659076	0.00429224558812677
RCAN2	0.790121047799009	-0.235569120196794	7.99920049468804e-05	0.000749161486329784
CKMT1B	1.05795335717112	0.0563362466146956	0.00829353780527928	0.0289430983976921
PRR15L	1.10110275554328	0.0963121826710826	0.0129261279510883	0.0407980913456225
ECE2	1.19695015269717	0.179776782180873	3.12670622946085e-05	0.000382557334630709
LST1	0.898494866303097	-0.107034286405234	0.0144688242018232	0.0447953224173137
RPS28	0.854470813026429	-0.157272933795771	4.70810767717836e-06	8.56942142938012e-05
PLCH2	0.837140708709382	-0.177763111857795	0.000191272626972989	0.001526567502462
CLIP3	0.85810827161543	-0.153024996761576	0.000295760268785582	0.00213669164728546
AFF3	0.939120802473498	-0.062811157917072	0.00194124615509607	0.00934519527560737
SESTD1	1.1348104663073	0.126465646951355	0.00532005154233492	0.0207132165491815
NES	0.897228724550268	-0.10844446107699	0.0146317225969081	0.0451846541346966
RUSC1	1.18467310052893	0.169466871996923	0.00112545787141544	0.00612815380091009
ID1	0.821460067261516	-0.196671952227969	6.96948466970484e-06	0.000117319658606698
CNIH2	1.20962540139919	0.190310726056626	0.000134546245346367	0.0011417589946938
C2	1.11562978537621	0.109419075389519	0.00833537450725863	0.0290497388950536
RPP25	1.1770696095641	0.163027968045148	0.000985951343776948	0.00550231351223733
PLCL2	0.833013085201604	-0.182705928413337	0.000196491294365142	0.001557117934269
LAMB3	0.849955947580826	-0.162570757216306	8.1244650715447e-06	0.000132016150105763
SERPINA1	0.9297285355937	-0.0728626326561939	0.000235352785412726	0.00179334644624401
ICAM2	0.858259355933967	-0.152848945528143	0.00455056659363795	0.0182554271992438
SLC40A1	0.918181397121964	-0.0853603075313014	7.11926494397604e-05	0.000693219919214
RHEX	0.856403242658849	-0.155013935890255	0.00108771595732218	0.00596045201719846
C14orf132	0.899848724106047	-0.105528614112195	0.00131846907681074	0.00694465696489218
SPRY4	1.18070686069275	0.166113293613417	0.000858643449502429	0.00494728457313984
CPXM1	0.828672677305114	-0.187930042220802	1.05248675569508e-05	0.000162803581939501
FBXO15	0.880492289500285	-0.12727410804017	0.00663576986006615	0.0243800645857352
CD79B	0.888730514109372	-0.11796122315779	3.72821398398591e-05	0.000427706686670633
TCL1B	1.27719281400788	0.244664555478645	3.12536961273492e-07	9.99924153738981e-06
PRKDC	1.12983817923272	0.122074418250712	0.0162361158292684	0.0487367323056887
SERPINE1	1.15516672124588	0.144244680955706	0.000330938178860063	0.00232559694312167
TSPAN32	0.83051769248535	-0.185706046741674	0.00098379356900086	0.00549622632746576
MZB1	0.924651395930882	-0.0783384817666919	0.000535460633267224	0.00340744264548016
ARHGEF6	0.883063595353523	-0.124358059053711	0.00179757765147374	0.00879327871105532
GPIHBP1	0.829798478293769	-0.186572404907937	0.000388162050926305	0.00264824201896874

TTYH3	1.11188654446641	0.106058162265597	0.0121760542166899	0.0388833572660694
SH3D19	0.743248114956391	-0.296725353324582	6.32340282836981e-08	3.4294988000839e-06
FRG1	0.864566051571175	-0.145527572415195	0.00195512111292488	0.00940320154311491
CIDEC	0.912980138448302	-0.0910411527877567	2.06250406162193e-05	0.000275946971984794
SLC11A1	1.20651454187043	0.187735658948918	7.84055935775328e-06	0.000127806079910719
MARCHF6	1.12085668418951	0.114093289520464	0.000458915557697054	0.00302946259372099
ADAM8	1.23250665182604	0.20905002393924	5.89694884126755e-05	0.000612348288952415
TRIM29	0.867934422023478	-0.141639117840077	0.00203995917966377	0.00973848909587405
LZTS3	1.10956652252605	0.103969418804549	0.0106084775806453	0.0351410083716425
PPARG	0.902929159104965	-0.102111179246144	0.00578929056217579	0.0220403811424741
ERGIC1	1.22359460525155	0.201792924369844	2.4666667018924e-05	0.000317645004536193
ZFP36L1	0.920200183875721	-0.0831640414393146	0.00695505718499801	0.0253362797453499
TSKU	1.11074299022507	0.105029151965504	0.000601220858099834	0.0037267011312542
LOC100132057	1.17307412531332	0.159627760610232	0.00244867924307518	0.011244414783438
PPME1	1.23997007851644	0.215087249097132	7.25442512369532e-05	0.00069976673805533
RPL5	0.806724016346134	-0.214773656382652	2.18116095139007e-05	0.000287344247074431
GDI1	1.19783507992337	0.180515827381607	0.0007252682305206	0.00432946738710646
DCBLD2	0.797744687776148	-0.225966672854231	3.02621700054559e-05	0.000371143899281198
FABP5P3	0.870061540941601	-0.139191333120747	0.00622080933158497	0.0232535477989798
MYBPC1	0.884540891310957	-0.122686535460575	6.31840489277178e-08	3.4294988000839e-06
ALDH2	0.873931408258819	-0.134753386659405	0.000209383696659947	0.00163895924806962
JAM2	0.767229169970922	-0.26496973479717	1.24666686742195e-06	3.08729857408196e-05
EXOSC10	0.87722224799051	-0.130994900253746	0.0103732258313972	0.0345688832855266
PCYOX1	1.13193916877203	0.123932240503164	0.002169168395295	0.0102414174190326
CYB561	1.20792818088397	0.188906644834605	6.49226513729117e-06	0.000110733965967506
RYBP	1.16528086272955	0.152962141849637	0.00118600362255932	0.00638359943553089
ENPP2	0.880852451462518	-0.126865145548186	7.21216173315191e-05	0.000697273705984921
TMIGD3	1.18177103204947	0.167014187909159	6.47256299965601e-06	0.000110733965967506
STEAP2	0.917357999659203	-0.086257479795144	0.00552216303859132	0.0213388310666046
EGR1	0.918394227601901	-0.0851285387132637	0.00073260286150563	0.00436763580974016
KCNMB4	0.839100668404733	-0.175424593533039	0.00288125568312892	0.0128604922803744
MAD2L1	1.10591680073936	0.100674674899292	0.00257505910804619	0.0117485646284729
MICALCL	1.12494073116696	0.11773035086142	0.00345076937750039	0.0147632168301533
CHCHD10	1.14139595051037	0.132252031287271	0.00849683572589796	0.0294728625078117
TWSG1	0.888845543064534	-0.117831800897787	0.0147891015538904	0.0455069666093726
SPDEF	1.08283155708824	0.0795794223010462	0.000772181216670652	0.00457710638328024
EIF3M	0.903112827911144	-0.101907785517612	0.00749667123994057	0.0267234280670823
FUT3	1.16145067312664	0.149669804075673	0.000150214691999308	0.00124598370126962
RUNX2	0.850423845281355	-0.162020412270663	0.00234073594715954	0.0108818870612083
PRTFDC1	0.819463410105803	-0.199105530842271	0.000678328275622716	0.00413499283755339
RTN1	0.945634500035382	-0.0558991481868665	0.00436233595202525	0.0177211297230931
RPL37A	0.895664058362984	-0.110189871175597	0.0054577902256617	0.0211854389241774
COL17A1	0.813927880270066	-0.205883516081558	9.13060857766137e-12	5.87897059794172e-09
PVT1	1.2068489639276	0.188012800836997	3.38709724583045e-05	0.000403220930882861

STOX2	0.853852351307833	-0.157996990881137	0.00051194257358424	0.0032850682404959
TTC39C	0.87742138879047	-0.130767913079096	0.0055011394329827	0.0212895335982674
FOXM1	1.17866959124694	0.16438633736575	1.06692088506239e-05	0.000164542199968754
OAS3	1.09711498191373	0.0926839906716159	0.00651593237420157	0.0240426702432036
ACOX2	1.0551477582046	0.0536808122914553	0.00471606811989705	0.018773158335077
LHFPL6	0.861752919831291	-0.148786685332949	3.27595459811097e-05	0.000394136672664617
TMEM131L	1.15311195096035	0.142464331942378	0.00991571019484215	0.0333393101916657
IFI6	1.05387834885549	0.0524770249147553	0.00661222873241015	0.0243109137763345
C1QTNF6	0.890680887621664	-0.115769066480086	0.00443362357587849	0.0179189246084112
SAPCD2	1.20083155359085	0.183014278132826	3.57578796377194e-07	1.11629598796299e-05
BTG2	0.892513728294154	-0.113713383604453	0.000148437339350649	0.00123521928109078
HES1	1.1723409690377	0.159002578056177	0.00258235321695187	0.0117714171863001
SCP2	0.841421718976191	-0.172662295256331	0.00216380904831927	0.0102254866127455
MAATS1	0.874467303562928	-0.134140373944094	0.00679060806777375	0.0248779673948098
NDNF	0.841563993261644	-0.17249322158594	0.00418964907574382	0.017188191166583
TGFB3	0.873660841350594	-0.135063032013402	0.000113696027244007	0.000992624129379456
SPARCL1	0.907765763646427	-0.0967689032131051	9.13332633331349e-05	0.000829731286470861
RGS16	1.12759771076613	0.120089450073183	0.00494711342875192	0.0195119305294802
CDKN1B	0.883449752682961	-0.123920861864097	0.000528172933358037	0.00337545754308592
SCARA5	0.710559843603532	-0.341702107541482	1.49011358491472e-08	1.09651072512796e-06
CYYR1	0.806468263708329	-0.215090732831763	0.000146839669925108	0.00122787522692245
STT3B	1.17425991272937	0.16063808764311	0.00292988029617056	0.0129989779548446
MMRN1	0.850548260904604	-0.161874124600196	0.00135886646782084	0.00711333452819628
F2RL1	1.23819097979843	0.213651427145457	0.000127977398935672	0.00109503585036154
KIF20A	1.27229366582282	0.240821307629266	4.49532384672889e-11	1.92961776120838e-08
LPAR1	0.864198326641961	-0.145952991794949	0.00047905559560646	0.00312752265268552
HLA-DPB1	0.902904695016292	-0.102138273752782	0.0116994470423145	0.0377829791316375
PLVAP	1.15464818680185	0.143795697402139	0.00616802404113721	0.0231423396336928
SNHG5	0.915242540963458	-0.0885661767408042	0.00618741643417063	0.0231718879340738
CCDC74B	0.82388028812809	-0.19373004102498	0.00024736057470811	0.00185221629688686
CDK1	1.12774533421745	0.120220360048816	0.000566429630507846	0.00354087260527417
HM13	1.11313989759808	0.107184758552762	0.011540113418346	0.0374627209126635
IL17RB	0.931823038217669	-0.0706123554817434	0.0144708632309086	0.0447953224173137
AXIN2	0.878544995923813	-0.129488153629853	0.00241018926477028	0.0111444209181613
MED1	1.11443919850638	0.108351317403953	0.00476061890424979	0.0189066676760144
CAV1	0.8773861316563	-0.130808096556502	7.26986578087451e-05	0.000699945395089432
SPR	1.10432846154346	0.0992374231187515	0.0155650889162713	0.0472177697336359
GPSM2	1.1693248500892	0.156426531045728	0.000830436710609362	0.00482888832508824
CST6	1.08145576979003	0.0783080684780679	0.00548307902432991	0.021267575342111
HYAL1	0.853550117306893	-0.158351018721265	0.00177013470384565	0.00869205325024684
GAS7	0.771871599882702	-0.258937064185761	1.04399924142401e-07	4.93361476383033e-06
VTCN1	0.950779117730622	-0.0504735065784479	0.000453441582300962	0.00300215628590264
VPS37A	0.816361521170979	-0.202897981480852	0.000138831160908544	0.00117232673744248
ZNF680	0.825950159940944	-0.19122084633618	0.00107078279556421	0.00588147486432431

TIMP4	0.892778796725349	-0.113416436824664	0.00608767215422639	0.0229222216859796
KIF11	1.18985309704088	0.173829851638315	0.00055808319102204	0.00350571526457869
TRPV6	1.12235891787303	0.115432647098675	9.16847592959959e-05	0.000831458090024076
CXCL13	0.9045420706581	-0.100326462615862	2.00180604475801e-05	0.000269929396244725
EBF1	0.810252193291804	-0.210409730031957	9.18117972027957e-06	0.000144931040670412
SULT2B1	1.13470904944572	0.126376273970916	0.00029140276374836	0.00211410652967296
AHSA2P	0.880141629360384	-0.127672442004751	0.0110753101822754	0.0363137636848509
LAD1	1.06654062083816	0.0644203461909603	0.00192412836736956	0.00928013597408298
CHD8	1.14228557552571	0.133031146107091	0.00442859769914093	0.0179189246084112
APOBEC3G	0.891568909708258	-0.11477254830243	0.00358075619874656	0.0152098630795213
HLA-DPA1	0.931322689076128	-0.0711494569507696	0.0106080494485873	0.0351410083716425
GIMAP8	0.853645150573346	-0.158239686094777	0.00151733113196742	0.00776915771447733
VPS28	1.19154123891031	0.175247627876987	0.00144211834272491	0.0074656799832925
FZD7	0.891888215623943	-0.114414473062424	0.00275101405479508	0.0123976145199033
GAA	1.15824063827009	0.146902162631482	0.00708003938205498	0.0256464717700177
NEFH	0.874873535001974	-0.133675934496511	0.00571304369358856	0.0218632155019871
ITPRIPL2	1.11912524028256	0.112547344677899	0.000512116054575462	0.0032850682404959
ACER3	1.16350229482737	0.151434676106667	8.09160037247502e-05	0.000753848877293937
NDRG1	1.15960406801608	0.148078626179318	1.22578384334095e-06	3.06383912129239e-05
DRC3	0.854221181239919	-0.157565124300699	0.00024214418812218	0.00182886321556796
DNAJB6	0.894929973373305	-0.111009805809349	0.00373746764555225	0.0158319867123681
LTC4S	0.872267862731916	-0.136658720211884	0.012242396804949	0.0390709950076159
RRBP1	1.20327811515299	0.185049594939315	5.91012606278893e-05	0.000612348288952415
RPL26	0.875917635277972	-0.132483216121529	2.00816072021933e-05	0.000270079265531326
UBL3	0.875566806695913	-0.132883823261786	0.0127027881329843	0.0402658841064629
ABAT	0.847286476220936	-0.165716416933144	0.000439015329851686	0.00292544367925748
MEOX1	0.783790720779312	-0.243613232047451	2.08789832202541e-07	7.66690459970287e-06
BIRC5	1.14967009697964	0.13947502902784	2.90721972316993e-06	6.06278898544467e-05
CFD	0.918409630549777	-0.085111767246013	0.000195447370003024	0.00155245567550324
ATL3	1.23010822114986	0.207102150188581	2.65024872434284e-05	0.000336242147268225
TXNIP	0.807300444162479	-0.214059382398636	1.1254411653432e-07	5.1760245023954e-06
CD27	0.861791409686468	-0.148742021723753	0.00012281898028736	0.00105969944298189
LAGE3	1.10720177915327	0.10183591277419	0.0147165965790319	0.0453443538878462
SESN1	0.798116661369142	-0.225500500025458	9.58293769894937e-05	0.000858464558039795
TNFRSF17	0.857585939393831	-0.153633884096376	1.55620957894062e-05	0.000221437445887379
ADGRL2	0.859336473099061	-0.151594730435065	0.00413441874107379	0.0169963215764334
TUBA3D	0.904923970408499	-0.0999043494096426	3.24606779197286e-06	6.55705693978518e-05
SPINT2	1.1998801119155	0.182221645065864	0.000706241756218746	0.00425977902375031
RPS27	0.921187997131112	-0.082091140721978	0.000502948142245289	0.00323835735088186
DLD	0.881323845699264	-0.126330131828221	0.0107834306025053	0.0356283227883459
GZMK	0.916742593824365	-0.0869285507932335	0.000247393673511582	0.00185221629688686
RFTN1	0.901109726744892	-0.104128245498114	0.014828275557224	0.0456002671016482
SRP14	0.871237989600354	-0.13784010225309	0.00530826656005227	0.0206829659991144
GNG7	0.743109432432124	-0.296911960544241	3.29637688489932e-07	1.03534373988515e-05

RDH13	1.22047044009185	0.199236391050695	7.61591854026027e-05	0.000721132286780894
CUEDC1	1.1808831702579	0.166262607900122	1.97192922414723e-06	4.37819285930275e-05
FAM83H	1.15396255587719	0.143201720312678	0.000155597054029434	0.00127827819028008
UNC5B	1.24944540051772	0.222699773273474	2.48862959899425e-05	0.000319360964512848
MAMDC2	0.788094201598879	-0.238137651092971	6.21287607601798e-08	3.4294988000839e-06
ZNF827	1.19893695445754	0.181435292892719	3.39091757403307e-05	0.000403220930882861
P2RX2	1.172628610106	0.159247904112688	0.000423858350182015	0.00283608613130032
ST3GAL1	1.17823534262412	0.164017846790255	4.53534545584498e-06	8.40604698186655e-05
FHOD3	0.856458186572352	-0.154949781356112	0.0020911686224476	0.0099460845560735
BAG3	1.16420552386945	0.152038900621295	0.000725360193180522	0.00432946738710646
C5	0.788415542986203	-0.237729989303529	3.25617354880028e-05	0.000393182445224159
CCDC167	1.15274805923973	0.142148708511615	0.00464439562469197	0.0185595669998358
CCL21	0.868614482126926	-0.140855886067465	1.10316392238348e-06	2.7779961306245e-05
LDHB	0.898081108106973	-0.1074948939268	5.73797516099306e-05	0.000598305871544033
TMEM101	0.847389717351317	-0.165594575213882	1.92230123676107e-07	7.31253667275701e-06
KCNG1	1.1096544070692	0.104048621863626	0.0120779279614464	0.038617881396282
MT1M	0.845294251599926	-0.16807048549635	0.000225968224338225	0.00173985399636203
JAM3	0.874017074859213	-0.134655367065247	0.00336172377261041	0.0144301992939302
ANXA8	0.874104494345479	-0.134555351725492	5.07183469343725e-05	0.00053977315094825
RPS6	0.773579063460375	-0.25672739898853	1.21075705516814e-07	5.42313877493139e-06
ARL4A	0.89792452279609	-0.107669264550795	0.00174233552931742	0.00858879415640068
PID1	0.826189436324012	-0.190931189959344	0.000577883931097829	0.00359937137736991
KLHL5	0.825365753545394	-0.191928653261767	3.39735748404507e-05	0.000403220930882861
CD59	0.825172431543047	-0.192162906555605	2.61878103707642e-05	0.000333070151160016
ATAD2	1.21141429975803	0.19178851981899	1.59117979129306e-06	3.70867289816766e-05
ATG12	1.20447347767689	0.186042523467049	2.49239191873743e-05	0.000319360964512848
SH3RF1	1.1663595544309	0.153887406114544	0.00193894175591929	0.00934283347496749
ARID5B	0.900214468862591	-0.105122245310248	0.0119420292397544	0.0382842744754944
CORO2A	1.11756427515968	0.111151562772962	0.0118161228039007	0.0379930390529917
TTLL12	1.10854508715294	0.103048423268663	0.00566479586725285	0.0217919070292901
KIF4A	1.3812178620766	0.322965618738381	4.98867957643004e-10	1.35245728937848e-07
SCML1	0.841711434108767	-0.172318038315154	0.00041153061991195	0.00276931132380213
FGL2	0.891319598077003	-0.115052219893549	0.000349963449756648	0.00243274187543386
CTSK	0.930241887706274	-0.0723106323456749	0.00444002429715134	0.0179189246084112
NECTIN4	1.19500599930002	0.178151205705619	0.00035654149772123	0.0024751283756901
PTPRCAP	0.90242767817504	-0.102666726972186	0.0138649380154461	0.0432575988598202
TMEM106C	1.14326861855674	0.13389136906238	0.00759318025740956	0.0269927339585346
WASHC5	1.12661867575471	0.11922082443562	0.012917148911254	0.0407948114322523
TLCD1	1.1954442616507	0.178517883366931	9.13419043286542e-06	0.000144769892060584
PCM1	0.86901295000002	-0.140397251641338	0.00181219536714977	0.00883123778258135
C1QC	1.08601024900042	0.0825106588524929	0.0119907254137376	0.038386716349386
NTM	1.1727262780629	0.159331190405475	7.74041714318076e-05	0.000731575939532552
RPL13AP6	0.846941660887157	-0.166123464049158	0.000203255121697486	0.00160331873179748
GEM	0.907272466846458	-0.0973124695180212	0.00721562510676843	0.0259550872381035

DSC3	0.874538449946153	-0.134059017569912	0.00618776172228226	0.0231718879340738
ALG1L	1.08327830845704	0.0799919141900708	0.00168743303078859	0.0083904158422581
BZW2	1.23507695592865	0.211133280631433	1.13612345650032e-05	0.000172122703659799
CILP	0.935431771171761	-0.0667470689349372	0.000775039291514547	0.00458876711562233
LDLR	1.12641636878208	0.119041238260374	0.00355607669198075	0.0151608595545713
ACAA2	0.840888747039084	-0.173295914276964	0.00168753073433884	0.0083904158422581
CASP17P	0.821477136788102	-0.196651172947367	0.000411821485931359	0.00276931132380213
CENPM	1.17939997943904	0.165005817150856	7.8701233032945e-05	0.00074111526755521
NCAPG	1.21201491317393	0.192284192170377	2.96843181521487e-07	9.80153351293065e-06
SIX1	1.19392760692404	0.177248382415412	6.04570701824909e-06	0.000106648756339045
NUCKS1	1.11783402858514	0.111392909853097	0.0096685627209828	0.0327522907132124
ARFGEF3	1.20783950420094	0.188833229925043	0.000450047065257824	0.00298736138291631
MAP3K8	0.868889737844254	-0.140539045720872	0.00475684289551892	0.0189062482676065
LAMA2	0.769594932641186	-0.261890964058261	5.90710404376773e-10	1.38306786042944e-07
PIAS4	1.16938834407842	0.156480829272114	0.00305472177134358	0.0133913802929283
C8orf33	1.23303651116424	0.209479835393623	4.56920069224223e-05	0.000500764952462547
EMCN	0.747276407598243	-0.291320138732595	8.70739932456187e-07	2.28835785310297e-05
PARP3	0.78651440402169	-0.24014424257434	1.94893579503677e-06	4.35405392661132e-05
KIF2C	1.20771807741114	0.18873269264882	6.49702231566254e-07	1.81881314934662e-05
THEM6	1.1758595339179	0.161999398395079	4.47090119349725e-05	0.00049208572751505
PIK3R1	0.894908462833265	-0.111033842101795	0.0115987350920293	0.0375283193838211
CDC20	1.15327861629565	0.142608856757867	7.97003614188172e-09	7.46430112124231e-07
KDELR2	1.13160102904129	0.123633469813267	0.00378528726812031	0.0159819792771211
ORMDL3	1.16340234284918	0.15134876628756	1.55030427224133e-06	3.62982604832504e-05
SORBS1	0.901563482143094	-0.103624820467389	0.00918181529043053	0.0313838955282068
FOXP1-IT1	1.11485628582459	0.108725504967706	0.000382954452626069	0.00262453416947901
ANKRD29	0.801130928024214	-0.221730889560577	2.14652606586636e-06	4.66987412454322e-05
WNT7B	1.33631639446148	0.28991686927821	8.65945540060266e-09	7.60126923954366e-07
RPS11	0.853174494269664	-0.158791187030488	2.90184484529397e-05	0.000361922585910635
NUSAP1	1.23047279646487	0.207398482896236	1.04693614490204e-08	8.69801303611356e-07
RAB37	0.791472662574691	-0.233859939013464	0.000106962986035934	0.000942834790671545
TFPT	1.18465048622456	0.169447782747575	0.00237586180452103	0.0110246812789499
LRRCC1	1.13359378337108	0.125392925408829	0.0038296939756554	0.0160880735127166
CD1C	0.691125371728126	-0.369434036464715	1.3071922399005e-09	2.11592032216888e-07
CTSG	0.898924397187592	-0.106556344612358	3.16884056857894e-05	0.00038587937987589
CBX7	0.723992218475191	-0.322974634616624	6.00331330133063e-09	6.31082996227634e-07
PTTG1	1.2010276636519	0.183177576680949	4.99810555070943e-09	5.47771099823495e-07
ELF3	1.19733903529616	0.180101623978903	2.43509814341057e-06	5.22632939029493e-05
LAMB1	0.851454064115912	-0.160809727350544	0.00138778085672299	0.00724261316411362
S100P	1.07983891206943	0.0768118745200713	3.18021645658962e-09	4.22956691258324e-07
PAFAH1B3	1.22328002216914	0.201535793840463	1.49208421665521e-05	0.000213492383333083
EPB41L3	0.867965102006523	-0.141603770191642	0.000240974267520107	0.00182269963582389
MFSD3	1.1206758048461	0.113931900520136	0.00618996656907116	0.0231718879340738
WDR97	1.13747072989142	0.128807139548306	0.00300348195620825	0.0132146636465032

KLC1	1.2006093364223	0.182829208269054	0.000493970290163394	0.00318702939448312
ADH1C	0.798003204586125	-0.225642665768305	0.000425810393731213	0.00284112608563404
HSPB2	0.8237277392987	-0.193915217143675	0.000654313201017801	0.00400757110397466
CYP2J2	1.07094746926542	0.0685437419577731	0.0123705298412795	0.0393337032175497
GNG11	0.878210009351795	-0.129869523342843	0.000245499980512854	0.00184339708399666
HCST	0.900218660562802	-0.105117588986008	0.000414453270692699	0.00278337522469113
FBXL6	1.26242655174756	0.233035703641428	3.11569170901068e-07	9.99924153738981e-06
CD3D	0.928246976777584	-0.0744574428356285	0.00176338350330505	0.00866716452817207
JAML	0.80743843068021	-0.213888473627916	2.42584195423889e-06	5.22632939029493e-05
ITM2A	0.84485308491964	-0.168592530741487	3.25792152038722e-11	1.67815537515146e-08
SNCAIP	0.805753356447128	-0.215977592676739	8.31894429533355e-06	0.000134751201463092
DUXAP9	1.13353070045443	0.125337275251887	0.00186655530712682	0.0090448037507152
BOP1	1.16440443656035	0.152209743050702	6.75632447662966e-05	0.000669265911136912
SUB1	0.911256566885029	-0.0929307892270416	0.00524595038746264	0.0204556324343831
ARHGAP15	0.865042065668001	-0.144977142402639	0.000320568010673532	0.00227131474962773
CALML5	1.06823182113071	0.0660047779674898	4.40422525277206e-06	8.24951428255595e-05
FAXDC2	0.858491933648381	-0.152577994555514	6.46275808053423e-05	0.000654020960173513
PAPSS2	1.10027929231286	0.0955640496792437	0.000458349232848216	0.00302946259372099
KREMEN2	1.09982726116378	0.0951531321673345	0.00720748522173581	0.0259439247918666
TMEM132A	1.20111652781859	0.183251564051936	9.1444480205874e-08	4.48600492895674e-06
SAA1	0.9237397201674	-0.0793249351525261	0.000460506544531845	0.00302946259372099
IRS1	0.86060541767925	-0.150119163362442	6.71353104735274e-05	0.000669140808847623
FAM110D	0.814462658936712	-0.205226697372538	1.34695861420642e-05	0.00019880182870422
FMOD	0.919366463701173	-0.0840704725628935	0.0150542203601646	0.0461041817572321
TMEM71	0.751175098063052	-0.28611650118087	2.2767590325261e-07	8.14415678926524e-06
DOCK10	0.880914802667436	-0.126794362980514	0.00989108249789377	0.033299977742909
TMEM106B	0.824992979976377	-0.192380401803202	0.000893164414715179	0.00509489468460452
PCSK5	0.838155808709559	-0.176551266535071	0.0002096824198171	0.00163895924806962
ULK1	1.1924545964687	0.176013868815432	0.00187666450684582	0.00908852433033485
MSL3	1.11242846840337	0.106545434926666	0.00804833712924833	0.0282597031716143
MTURN	0.861994445467618	-0.14850645211227	2.98569357104896e-05	0.000367926018767301
RAB8B	0.76514766605435	-0.26768643626099	1.39414453720729e-06	3.35692743966551e-05
SNX29	1.14318392204343	0.133817283541821	0.00389481544341327	0.016297477131618
JAK1	0.87884444791642	-0.129147361784819	0.00213844782584267	0.0101250243584895
DENND6A	0.854460155074864	-0.157285407034028	0.00437253604277388	0.0177485682870987
HOTAIR	1.14282555086786	0.133503749251827	0.000330167590863045	0.00232335144881905
SUSD3	0.908568376510713	-0.0958851308952468	3.79572246206618e-07	1.17076445521574e-05
MBD6	1.14889491378672	0.13880053583868	0.00224156250522266	0.0105157454138451
CYC1	1.24598197091212	0.219923950687637	1.27192740705634e-05	0.000189904291992672
CH25H	0.817589661820509	-0.201394704157027	1.39257717816625e-06	3.35692743966551e-05
TCN1	0.95199016960769	-0.049200570286428	0.00100097567791596	0.00556205578958478
EXO1	1.21303746291298	0.193127513996936	2.49468852507223e-07	8.68252742746423e-06
MYH10	0.889471270940827	-0.117128070384542	0.0166812616815098	0.0498440005290819
CHTF18	1.20291344582646	0.184746485797682	4.33879174705285e-05	0.0004795947701517

CSN3	0.907138501379168	-0.0974601377915045	0.00331199625030715	0.0142762281885625
CYP4F22	0.867733215682112	-0.141870966770019	1.46017790562468e-07	6.26781365989393e-06
METTL7A	0.82367778291254	-0.193975865703488	0.000296993328132662	0.00213959808840747
ATP8B4	0.774772904784151	-0.255185318654288	3.51635284340587e-05	0.000412160644427604
DUXAP10	1.16671047708913	0.154188230912888	0.000780732589789456	0.00461716827784786
CTSO	0.797624086809794	-0.226117861680922	1.19825631898162e-07	5.42313877493139e-06
MAPK9	1.1561979986536	0.145137034695702	0.00908455622432218	0.0311340978785652
PTGDS	0.927262745862002	-0.0755183168325483	0.000950676574129983	0.00534016906580539
NME1	1.11137148664388	0.105594826184466	0.0121010495507129	0.0386678078385373
PAPSS1	0.87266069567682	-0.136208463438165	0.00241673192426405	0.0111646512483266
PTGS2	0.876171449538509	-0.132193488544877	0.000286475742966433	0.00209013675923526
PODXL	0.894854964821466	-0.111093624317688	0.00694458152684072	0.0253339514481279
SYNE4	1.15735916348108	0.146140826556985	0.00131990277006978	0.00694465696489218
NFIC	1.16466478363243	0.152433306241447	8.67649309038166e-05	0.000798082426938499
RPL39	0.874653580301453	-0.133927379243787	0.00637958159883588	0.0236557291007993
GZMB	0.935590484158504	-0.0665774151676753	0.010482356315687	0.0348802437868887
LBP	1.06572898736758	0.0636590601822358	0.00823971617504156	0.0288138343636382
CLEC11A	0.89082301174461	-0.115609511260142	0.00410709437730963	0.0169516371294246
ABHD11	1.15919378416489	0.147724749833361	0.00133712656848699	0.00702609634351183
GBP2	0.903333920916292	-0.101663003315872	0.00572736304155564	0.0218764976669572
IFI27L2	0.833023967307375	-0.182692864951447	0.00012843243465657	0.00109710691694195
LINC00857	0.844851661557023	-0.168594215488623	0.000369669893461893	0.0025456813117944
PPFIBP1	1.20916173377899	0.189927337523471	0.000144151984473224	0.0012112999543582
STAT5A	0.694675554513955	-0.364310370452951	1.62105699058172e-12	1.47412728885042e-09
AARD	1.06339723653073	0.0614687233737344	0.00857598190592499	0.0297273773872272
UNC5A	1.07617413459835	0.0734122837742749	0.0111254505746653	0.0363855212127625
F2RL2	0.804698107034534	-0.217288094219378	2.26982429804814e-06	4.91254830220419e-05
APOL3	0.877547727871759	-0.130623934384192	0.00102692024697619	0.00567875113728741
SIAH2	0.908560725769606	-0.0958935515859923	0.00827142163573632	0.028904434384701
DACT3	0.877562539725442	-0.130607055839343	0.0156421909686429	0.0473548860536573
SLFN11	0.905880112685929	-0.0988483076487303	0.0146862340485972	0.0453258188042685
RAB3IP	1.10976064584149	0.104144357694772	0.010773016286144	0.0356173343324313
ZC2HC1A	1.11824268542311	0.111758422216744	0.00489786059761975	0.0193770199219196
TRIB3	1.25918944659499	0.230468217606327	8.59594179993121e-09	7.60126923954366e-07
PLA2G4C	0.866984863466836	-0.142733760869757	0.00100734776805022	0.00558707967733895
SCGB3A1	0.956252345679262	-0.0447334408464786	0.00717345604300093	0.0258756807265391
PITX1	1.09027280110001	0.0864279411657194	5.53291392648676e-06	9.93032739907084e-05
FAM189A2	0.882507922907873	-0.124987512415696	0.000143272501154681	0.00120645009590638
TMEM159	1.20131918892744	0.183420277085898	0.000488750446725942	0.00316673402652242
MFNG	0.822542112667564	-0.195355596841699	0.000196816383631268	0.00155729829813312
PGAP3	1.11405757021672	0.108008818999873	4.20778930335443e-05	0.00046812792012049
SRPK2	0.872690721682604	-0.13617405660698	0.00958427973802563	0.0325221508106522
AR	1.10444440453902	0.0993424072005915	0.000885463925549076	0.00506218055549755
RPL17	0.897675960518743	-0.107946121547933	0.00131076983353309	0.00692489785900404

EPS8L1	1.13948410605532	0.130575621431969	0.00433595504615652	0.0176278645957003
SERPING1	0.9030366667922	-0.101992120861074	0.00367352207623955	0.0155739195182798
EPN3	1.23600448178909	0.211883985071878	1.51981548465327e-06	3.57468929746529e-05
PAK6	1.15191942472942	0.141429616016249	0.00641107132398336	0.0237237272915505
CD48	0.875824452723645	-0.132589604561989	2.30809763775526e-05	0.000300987618533603
FMO1	0.847054365325494	-0.165990400645515	1.7088331681415e-05	0.000237443059687012
MRPL27	1.13798391784691	0.129258203659564	0.0114296969537394	0.0372085606770121
CX3CR1	0.884133261471808	-0.123147479434597	6.13841477246578e-06	0.000107914588713212
NTHL1	1.12719621987442	0.119733328043148	0.0116259920581803	0.0375693131064533
C21orf58	1.12526251282796	0.118016353171701	0.0131824289347918	0.041479958120411
BUB1	1.20180586870303	0.183825316166732	4.13842936674564e-06	7.90956610901929e-05
SLC52A3	1.1670459059736	0.15447568926905	0.00288708215545909	0.0128604922803744
SNRPB	1.2324081219837	0.208970078098161	0.000230326007957769	0.00176548998064058
C1orf21	0.90418773833287	-0.100718265004478	0.011582952280645	0.0375072401989574
PRC1	1.20616037319907	0.187442068899391	1.23661599884539e-08	9.50717762694417e-07
ACAT1	0.838250443187376	-0.176438364930451	6.74894727569481e-05	0.000669265911136912
ECI1	1.1354268387115	0.127008649586352	0.0154127743570603	0.0468661161235052
ISG15	1.06204428996705	0.0601956262505687	0.00561129333499414	0.0216507655195167
DPT	0.911093892148311	-0.0931093221062073	0.00157161474374372	0.00796790112699205
DYRK2	1.18043282514515	0.165881172193005	3.47977471341034e-05	0.000410167495395347
FKBP4	1.12964878037713	0.121906770586073	0.00276551858751829	0.0124520858778905
PIR	1.11938399654156	0.112778530911933	0.00763690910829966	0.0270931784842348
CYP1B1	0.901865569925969	-0.103289805585209	0.000277780344120272	0.00203824295236969
EIF2AK2	1.12349981595502	0.11644864882928	0.00887636290661286	0.0305833748039885
CMTM4	1.13822923154328	0.129473749112664	0.00504016231561866	0.0198637154458697
RAX2	1.11044943505296	0.104764829819904	0.00329947200347398	0.0142341543466453
SCD	1.07949427061583	0.0764926635161458	0.00820188949264882	0.0287010412884742
CMTM7	0.891743049013076	-0.114577249544386	0.000313665343828551	0.00223377193670004
SLC52A2	1.24850666933036	0.221948172597959	3.12083294081448e-07	9.99924153738981e-06
RACGAP1	1.35666585819013	0.305030114857569	2.80992303877583e-11	1.6082126191927e-08
PRSS23	0.924193483317514	-0.0788338317312785	0.00802868924934764	0.0282292002207438
EIF3L	0.833672787121804	-0.181914295190056	0.000269673508702148	0.00198441177617823
DIO2	0.838756484089764	-0.175834860067689	4.92612472580952e-08	2.95051958867963e-06
RNF183	0.879304341658094	-0.128624204979331	0.014999779062135	0.0460177855563177
CASTOR3	0.88118531835018	-0.126487325166212	0.016224687466605	0.0487308251548003
RPSAP52	0.925835140613813	-0.0770590940802429	0.00507428448519415	0.0199284638499292
IGFBP5	1.11474829785815	0.108628637597308	1.12382841411586e-05	0.000171203014857974
FGG	1.07005317563909	0.0677083440979335	0.0162222659806926	0.0487308251548003
RPL31	0.866778895964731	-0.142971356680552	4.16131317325612e-06	7.90956610901929e-05
TST	0.898286090666479	-0.107266674929552	0.0109221442188203	0.0358572115176184
LRRN3	0.828303783447474	-0.188375303662317	0.000958702804774764	0.00537353443677346
IGFALS	0.820690405421056	-0.197609335140949	5.39665870173593e-05	0.000568470122139913
H4C5	1.14702045539155	0.13716767180822	0.00164024161268217	0.00820163711642423
ACACB	0.885984494022718	-0.121055829633229	0.000542087310775794	0.00343437721112386

UBE2V1	1.22982691421312	0.20687343933038	9.28814616358625e-05	0.000839355103309347
TXNRD1	1.20988485595243	0.190525194710718	5.35999648925666e-05	0.000566926938730207
CDC45	1.18460790436953	0.169411837444854	2.89526384370956e-07	9.6216155219019e-06
SLC39A8	0.907656192967825	-0.0968896141897857	0.0125634370448785	0.0398978201098452
CALML3	0.808060037471959	-0.213118919419503	1.75887430032766e-07	7.11223132550011e-06
ECHDC2	0.901980075475883	-0.103162848436342	0.0108398507076402	0.0357236538675974
CD55	1.11045960809238	0.10477399096899	0.0113585192285352	0.0370067884542597
ARHGAP36	0.833179810428668	-0.182505801251521	0.00379046581621561	0.0159907366251651
CPA3	0.920694159976146	-0.0826273717342536	0.000329414954526725	0.00232122630747902
PLAAT1	1.14637062383606	0.136600972496648	0.00054272627642299	0.00343437721112386
GFPT1	1.18350241261837	0.168478188515127	0.00121361798916095	0.00649828093780463
KRT6B	0.957487597700896	-0.043442510804022	0.00984532343924022	0.033189306960423
RPS6KB2	1.24740889792233	0.221068518259314	1.62303639671198e-05	0.000228422417471678
TSTA3	1.20371784745415	0.185414973455006	0.000244361266160167	0.00183752537516937
ABLIM3	0.84370998178237	-0.16994646691886	0.00115507575293213	0.00624725862822632
FCRH3	0.859047279193158	-0.151931318706745	0.00549585840537561	0.0212853666558846
COMP	1.05290075749011	0.0515489813124642	0.0103753627566345	0.0345688832855266
PFKP	1.07819216516343	0.0752857174197452	0.0105371531474477	0.0350109516948687
MS4A2	0.872817450798148	-0.136028850623113	0.00491682322515624	0.0194222058533587
HOXC9	1.1361495496445	0.12764495744823	0.000387879545149188	0.00264824201896874
CEACAM5	1.17917684184522	0.1648166033907	1.43366479436315e-08	1.07026193561806e-06
TBX2	1.14790125625936	0.137935280486625	0.00607289545377994	0.0228833097896273
SNX29P2	1.14282856203742	0.133506384094556	0.00483996818493656	0.0192069923887583
RAD9A	1.16241731606665	0.150501729985629	0.00309129823888019	0.0134943027359931
TNFRSF11B	0.848233962430745	-0.16459878215053	0.000537683019043696	0.00341504960677445
MACROD1	1.24369135906948	0.218083859892294	1.96865917278131e-05	0.000266156519658701
A2M	0.864225102076239	-0.14592200930092	8.42570566627429e-06	0.000135613259606554
GSN	0.838848011955034	-0.175725742733328	4.15888775624716e-06	7.90956610901929e-05
MMP11	1.08649418047076	0.0829561644834935	5.62705496396222e-05	0.000587930225545018
C1R	0.910130788047196	-0.0941669666696342	0.00170150414925009	0.00842735372383388
NFE2L2	0.877866208838048	-0.130261078691727	0.00551726945154764	0.0213359271358272
MMP23B	0.863913603597483	-0.146282511014245	0.00020311190163015	0.00160331873179748
SLC9A3R1	1.08171059557372	0.0785436729040206	0.00891106217981982	0.0306824072782433
MBNL2	0.871827036672354	-0.137164227187565	0.00220911403453509	0.0104014135209234
ACO1	0.79697600165589	-0.226930711491072	3.94441253632296e-05	0.00044556291610964
SLC3A2	1.12482396787875	0.117626550416539	0.0139978716556634	0.0436195020558513
CCNDBP1	0.88282946717726	-0.124623225934541	0.00100764842171382	0.00558707967733895
SUGCT	1.10746697193358	0.102075400310597	0.00386454981536716	0.0162103388427983
PLEKHF1	0.878170600049517	-0.129914398926272	0.00144427127166678	0.00746931859473454
SRGAP1	1.11277499254743	0.106856888825546	0.00238284760978861	0.011037812983832
TSPAN6	0.894967291828196	-0.110968106818439	0.00394415151092821	0.0164504651277662
NFKBIZ	1.07647840254608	0.0736949749606236	0.00157554859617831	0.00797215208144842
INPPL1	1.15776020651387	0.146487282158364	0.001805706284048	0.00881629674799171
HLA-DOA	0.89524420542671	-0.110658742700125	0.00347228508054743	0.0148306305554725

SLC39A11	1.13081467096725	0.122938320724879	0.00196454845529194	0.00943093112135021
KMO	1.13373277174927	0.125515526520746	0.0041853515319516	0.017188191166583
CMTM8	1.13810111938084	0.129361188857491	6.82031965980075e-05	0.000673411112337845
PGM3	1.16279394048314	0.150825678546198	0.00736567315761874	0.0263842715124438
GPAA1	1.27342724059705	0.241711880391023	9.98476649623665e-07	2.57157661110575e-05
PLCG2	0.888997894690296	-0.117660411648432	0.00142840942509778	0.00741771726275673
KCNMB1	0.850386061060473	-0.162064843127688	2.96910493934794e-05	0.000367607013802928
NSMCE2	1.15959801723066	0.148073408190379	0.00873398325008373	0.0301937904169002
UBE2T	1.14092038325593	0.131835290404957	0.000234364869155043	0.00179111786501131
CD248	0.912310863221819	-0.0917744881911129	0.0131124180115588	0.0412852549290784
SPATA18	0.856351270521599	-0.155074624257288	0.00010271903154018	0.000909116377084992
PTPRE	0.795394331950324	-0.228917272270744	2.20409011572551e-05	0.000288887231198527
LUM	0.946912678264801	-0.0545483988492399	0.00564435443654057	0.0217294990303591
PLAC8	0.865477809425687	-0.14447354371745	0.000110467824311399	0.000971023486395934
USP14	1.13585247686759	0.127383449953839	0.0115849386830793	0.0375072401989574
THOC2	1.12363530587956	0.11656923786141	0.01054882489428	0.0350109516948687
PLEKHA4	0.853827734883119	-0.158025821134432	0.000256865143848024	0.00190650195383454
ADH1B	0.790097274509751	-0.235599208810405	1.68815494570289e-05	0.000235655450550558
CIART	1.12725449976933	0.119785030122431	0.00871339324839699	0.0301428399076514
COL8A1	1.07618401797058	0.073421467536025	0.00574295635828842	0.0219125690381805
CPM	1.11995401495769	0.113287626390603	0.0155836168420569	0.0472212165341275
SOWAHC	0.865894028604026	-0.143992746724898	0.00587430940184614	0.0222653184171519
GPR34	0.897792305320905	-0.107816523264813	0.00725743617681353	0.026050908534332
ATP2C2	1.19038022393365	0.174272771999727	3.75476851851016e-05	0.000429795836418796
PKMYT1	1.42548419265907	0.354511540329151	3.19041433873443e-13	8.21691212941054e-10
PIEZO2	0.911610020925751	-0.0925429889924931	0.00158983975152366	0.00802082718912673
DDIAS	1.19795510889111	0.180616027280913	0.000396702216383427	0.0026957956683259
TPD52	1.07707834717986	0.0742521412850048	0.0153823881086735	0.0468129428205238
TRIB2	1.15534382761831	0.144397985923613	0.00040350455258114	0.00273480519782296
SHC4	0.880099197518908	-0.127720653409505	0.00287216850369559	0.0128536402802224
HNRNPDL	0.854863218929537	-0.156813800703391	0.00262411562243579	0.0118986087774355
SLC26A3	0.887934163788888	-0.118857678615842	7.03426922306883e-05	0.000686240923636885
TMEM47	0.907184037662846	-0.0974099413322663	0.00900986094040983	0.0309605028045704
HSPB6	0.867470841993453	-0.142173379260928	0.000533832460323244	0.00340318193456068
SACM1L	0.87315661865077	-0.135640336425997	0.00696538770360366	0.0253559802553092
RAB26	1.07490068315538	0.0722282695491776	0.0111933973248895	0.0365470482585083
C1orf54	0.8363675552111	-0.178687103175865	0.00052952991043953	0.00337993626849321
SEC16A	1.18793379272592	0.172215489358793	0.00168009779576041	0.00837771901835612
CENPA	1.16640287919794	0.153924550717379	4.90958426173394e-05	0.000526193414399999
WDR45B	1.2184918521848	0.197613907302294	0.000339859241660359	0.00237532558180802
DDX27	1.25248430924736	0.225129026351396	8.5600015677266e-06	0.000136933441227825
MAPT	0.938874564299023	-0.0630733930431457	0.000920012990341449	0.00520196148545423
ENPP3	1.1374912617804	0.128825189862163	0.00181040292727312	0.00883085746059078
GPM6B	0.876073757667594	-0.132304993339541	0.00186346595044846	0.00903833626248589

CYB5D2	0.883744660965154	-0.123587103130306	0.0058345656874714	0.0221309630752321
CDT1	1.15424592614833	0.143447252962587	0.00030260734525785	0.00217250392710517
SMOC2	0.865156421807063	-0.144844953939806	7.42783772405633e-07	1.99036014273919e-05
RPL6	1.14463910153182	0.13508939215305	0.0115760016370168	0.0375072401989574
ASF1B	1.19266162972368	0.176187473148188	9.30501386869781e-05	0.000839406767734893
CHPT1	0.896124215166382	-0.109676242617678	0.00045970164084384	0.00302946259372099
GAPDH	1.14156090211422	0.132396538270786	0.00296800863069601	0.0131004391231492
ALDOA	1.12564588388282	0.11835698992007	2.97596825385014e-05	0.000367607013802928
BNIP3L	0.888992098711509	-0.117666931346302	0.00915318069209019	0.0313275971727286
VEGFA	1.25226936187436	0.224957394804542	2.99821472028931e-08	1.90664247212472e-06
RPS3A	0.823481630417615	-0.194214036331376	3.17667124842046e-07	1.01006380250703e-05
EZR	1.35376448789701	0.302889221323447	5.268890652353e-12	3.87715082146718e-09
CPQ	0.851613435478059	-0.160622569339895	0.000256307477746451	0.00190580243705266
RNF24	1.26263440671852	0.233200337267974	1.47387634460171e-06	3.49858850278497e-05
SFRP4	0.935475846368688	-0.0666999525551423	0.00142853339636084	0.00741771726275673
CCDC80	0.857022105033811	-0.154291567211974	0.0044006102578923	0.0178484594003175
FGGY	0.867694668983731	-0.141915390048829	0.00395589895973174	0.016486112897717
SDC4	1.11769064357725	0.111264631234771	0.00471018599599448	0.0187642444434397
FSTL1	0.895021929105983	-0.110907059213051	0.00159497659289517	0.00802316838867482
GREM1	1.11361297607542	0.107609662896709	0.00318914240042557	0.0138393197174323
CYP4V2	0.883313240536008	-0.124075395491696	0.00757461309482011	0.0269453260023608
CCNA2	1.20175416022418	0.18378228959096	8.45114663826514e-06	0.000135613259606554
CDH11	0.921657621091376	-0.0815814681093774	0.00710114617151041	0.0256868005122543
STC1	1.06308131753188	0.0611715945824966	0.00288867978419592	0.0128604922803744
OLFM1	0.906664356914129	-0.0979829558846301	0.000478082542465144	0.00312513093431213
RPL7	0.891313721147446	-0.115058813430754	0.000910537071081155	0.00516539256953638
KCNK2	0.89996131216088	-0.105403503069683	0.0164397166890857	0.0492904427622121
LEPR	0.786312202676802	-0.240401360994312	6.45557462346498e-09	6.52013036969963e-07
CYTIP	0.853961104030132	-0.157869631862965	0.00419682914484996	0.017188191166583
PTGER3	0.813725738742514	-0.206131900045113	1.84315562775491e-06	4.20092683122368e-05
ESD	0.867580885804029	-0.142046531387795	0.00261029692289951	0.0118749769655998
NCAPD2	1.14741464080917	0.137511273091298	0.00628252663630121	0.0234162769201068
JCHAIN	0.887399009965036	-0.119460555696013	1.03318983956868e-12	1.33049021590457e-09
FANCD2	1.31560207812812	0.274294414936355	2.44120182582927e-07	8.61276068825106e-06
MRPS17	1.15570180132777	0.144707779644555	0.00567781147633573	0.0217931497128207
SLX4IP	1.16317741667563	0.15115541278371	0.000494357694853817	0.00318702939448312
RPLP1	0.88819460787154	-0.118564406991988	3.72541922772206e-05	0.000427706686670633
SCPEP1	0.908489641592665	-0.0959717928700571	0.0111167476236485	0.0363855212127625
H2AZ1	1.12560682172341	0.118322287321542	0.00801750169939447	0.0282091197087301
PRNP	0.902645191453293	-0.102425724757853	0.000819959227548903	0.00479955679670955
ABCA8	0.786358496926044	-0.240342487576925	3.04547994747697e-08	1.91308136700657e-06
RPS12	0.920349262579746	-0.0830020477426624	0.00378041445908249	0.0159744994903478
POU2AF1	0.871668134955579	-0.137346506691175	0.0033998956510514	0.0145697691335821
MAP1B	1.18221802277557	0.167392354407447	6.65614236714821e-05	0.000667964298811124

CD69	0.88922330590637	-0.117406887264395	0.000691217536939831	0.00418385609021982
SORBS2	0.9013530258733	-0.10385828249385	0.000484733938144062	0.00314863116693577
PMAIP1	0.896557598498426	-0.109192739815136	0.00763721513475225	0.0270931784842348
NSG1	0.716565256374927	-0.333285959273655	2.5268932862919e-08	1.6903931581415e-06
POLR2J3	0.849098324771874	-0.163580286924385	0.00109548067087324	0.0059966216106993
LPAR6	0.755443530657051	-0.280450244395002	6.3713600577891e-08	3.4294988000839e-06
ADHFE1	0.793390609878694	-0.231439606264743	4.50151985751051e-06	8.40120608189733e-05
DNAJB9	0.847196560627544	-0.165822544396826	0.000903240472048674	0.00513746485052049
RASGRP1	0.870374828124825	-0.138831323192001	0.00907023294899688	0.0311057056726251
RPS29	0.862769984286294	-0.147607153799336	0.000159069534415276	0.00130472479581702
ENTPD5	1.13399358380535	0.125745547269581	0.00324249302573037	0.0140406186359156
SYNM	0.924714857514786	-0.0782698511384315	0.00427692841949764	0.0174844907054225
KLF4	0.922482069892046	-0.0806873396877315	0.0165947949566183	0.0496975516404308
S100A6	0.906713949090309	-0.0979282599879951	0.00742582163861993	0.0265259412347651
KLHDC2	0.85745890323233	-0.153782027350216	0.00282517645267966	0.0126763797105862
ABCB6	1.17332035132173	0.159837636666252	0.00135325754226948	0.00709117965435411
AQP5	1.13636433832987	0.127833989239984	0.00309651984816272	0.0135056509211568
HOXA10	0.85456721850371	-0.157160115384939	0.000149126614000836	0.00123895353019082
TRIM4	0.76929965232038	-0.262274720460182	2.73388234464395e-06	5.79515553796748e-05
PLIN4	0.923157996132535	-0.0799548823867104	0.000228098897569877	0.00175363794236185
AKNA	0.859536760666085	-0.151361685247094	0.00115252552109534	0.00624599890339238
P4HB	1.16402149435455	0.151880815079091	0.00814408246139365	0.0285375297677814
NPIPL3	1.08212415762987	0.0789259221128101	0.0154300601280994	0.0468909968848612
DPM3	0.805116911140575	-0.216767780878079	6.41741653829244e-05	0.000652151116730121
SLC25A10	1.17967205798259	0.16523648288759	0.000357112656795514	0.00247575679024723
FTO	0.882343339323477	-0.125174025113276	0.00507593565736887	0.0199284638499292
BHLHE41	0.826583546930535	-0.190454281614416	0.000205338413302849	0.0016172754845917
VASN	1.11593837741914	0.109695645068222	0.00537247862837629	0.0208857640866161
ZFP36	0.927051722735808	-0.0757459191413029	0.0135348579479443	0.0423560469561733
GET4	1.29368362544401	0.257493672710817	4.67742869353498e-06	8.54377134765911e-05
NVL	1.21150707923571	0.191865104621559	6.71609004415106e-05	0.000669140808847623
UHRF1	1.23546576694611	0.21144803820844	8.668754389541e-10	1.71741361002022e-07
OGN	0.825545623646844	-0.191710749254748	3.79623355583401e-10	1.08635550256117e-07
QPRT	1.11302556542833	0.107082041866378	4.54189970736602e-05	0.000498834230120306
PTGER4	0.788755415585015	-0.237298999115401	5.90059356983854e-07	1.66999766363947e-05
NDUFC2	0.922332262593844	-0.0808497487669468	0.0150391125957306	0.0461041817572321
ADAM9	1.13950405707958	0.130593130100578	0.0101967069919537	0.0340617624614484
H4C8	1.05533305051731	0.0538564048032886	0.0142762915461551	0.0443089985749614
PHYHD1	0.846315488085659	-0.166863071533376	0.000110885754842853	0.000973036666431921
SLC47A1	0.835899247958168	-0.179247189956618	0.00328783231193546	0.014207738455352
PKP4	1.20738487883629	0.188456763561734	2.43678137662009e-05	0.000314582979222308
KCNAB1	0.786672964757758	-0.239942663614642	6.99059181193699e-05	0.000683273973876422
PCDH17	1.25459419177072	0.226812167123098	1.10331029596372e-08	9.02087513414143e-07
ZNF296	1.10941157233093	0.103829759746416	0.0132574106644897	0.0416395867882845

SPINK8	1.09568194033129	0.0913769459818756	0.000106961525944099	0.000942834790671545
LAT2	0.833827749327648	-0.181728433533159	0.00129780222721557	0.00687048229433442
BCAS1	1.13475586735968	0.126417532959867	0.000114573322084711	0.000996904023747206
MFAP4	0.868664311179023	-0.140798521581923	3.52574739379179e-09	4.42954264034671e-07
SFRP2	0.949595832530875	-0.0517188243537699	0.0115420963091433	0.0374627209126635
HOOK1	1.11971513699511	0.113074310987945	0.00152362400780425	0.00777886361862536
TBC1D24	1.34312631050626	0.294999964128456	1.00217126504832e-09	1.91192006898662e-07
RAMP3	0.866234032277875	-0.143600161804771	3.33749469254681e-05	0.000398873205598807
SQLE	1.16706578709575	0.154492724548223	1.87558764006537e-06	4.21884363929114e-05
IMPDH1	1.14294760299417	0.133610542112018	0.0109080369323152	0.0358337361214002
IL23A	0.924251471222326	-0.07877108936539	0.00156940605767509	0.007964542466093
RPS13	0.843740332281035	-0.169910494898333	0.000749752894828718	0.00445441425751179
LRP2	0.905186197705807	-0.0996146131231314	0.000195601578994681	0.00155245567550324
C12orf57	0.837785813136251	-0.176992804085658	0.000239101517767873	0.0018165367522453
AMY2A	0.867964105659275	-0.141604918103946	0.00164160121666344	0.00820163711642423
PIM1	0.824641508995634	-0.192806521635699	3.52068886717425e-05	0.000412160644427604
CPSF1	1.14460084088894	0.135055965650065	0.00819619426167701	0.028700609545818
CCNF	1.26977813292191	0.238842186722256	9.89879474579134e-08	4.72520133602019e-06
RRP7A	1.12939012803209	0.12167777732243	0.00699100680035283	0.0253953991739192
SCGB2A2	1.02478680634196	0.0244845971439329	0.0138782528679782	0.0432729300986415
RPL12P38	0.825070066279415	-0.192286967430172	7.48970737659535e-05	0.000711798573742484
ECRG4	0.855648733908366	-0.155895344643793	5.38372104513269e-08	3.18753414982511e-06
SVIL	0.818472245178467	-0.200315792118557	2.70747808462799e-05	0.000342658958572942
APOBEC3B	1.12374939281724	0.116670766485183	0.00291382418417665	0.0129500503647057
GIMAP7	0.894196850815018	-0.111829336978866	0.000590947743200914	0.00367629447491293
CCNG1	0.848270726831631	-0.164555440801549	0.000222558713000092	0.00171616756087346
CD8A	0.915018296192717	-0.0888112180674944	0.00182602292938876	0.00888181691150282
DDAH1	0.843681695009804	-0.16997999413481	0.00064248372162027	0.0039398019643643
NUDT18	0.857213090024359	-0.154068744822271	0.00517321943676978	0.0202026181340418
MAL2	1.12656773394281	0.119175606861352	0.000214335011405856	0.00167025664712793
KLK5	0.943769005992984	-0.0578738398211729	0.00911616153404638	0.0312216409985857
IFT74	0.801286484407784	-0.221536737421626	5.48851557397687e-05	0.000575791114491952
RGL1	0.860437930571334	-0.150313797749327	0.000484277054557457	0.00314863116693577
OLFML3	0.872877980632759	-0.135959503091413	0.000557549303303911	0.00350571526457869
SYNJ2BP	1.17409751910346	0.160499783632001	3.32097830566512e-05	0.000397822308197233
SOX11	1.09607584019154	0.0917363833868595	6.87879066210946e-05	0.000677488541119041
ETV6	0.863153370274037	-0.147162886105715	0.0117123944623905	0.0378010926539935
PDXDC1	1.13666145109946	0.128095414141244	0.00751228795908697	0.0267420838128936
HJURP	1.22959939133053	0.206688418227922	6.47799205474772e-07	1.81881314934662e-05
LDHA	1.32811833951878	0.283763158167505	2.85415848602226e-07	9.54660413084459e-06
LYPD6B	0.924310709507655	-0.0787069981641671	0.00470076924073376	0.0187412247360833
DDIT4	1.14534058036178	0.135702042874319	2.09017664839749e-05	0.000278204132193681
TGFBR3	0.917420381481361	-0.0861894804958034	0.000291074329694068	0.00211410652967296
PLK4	1.29958709176549	0.262046592296175	7.10582108790552e-07	1.93661822348155e-05

TUSC3	0.929596629090889	-0.0730045190841705	0.0151929650545497	0.0463894267907441
KNTC1	1.17054575855491	0.157470100354218	0.00157482229842803	0.00797215208144842
SLC6A9	1.14214670076559	0.132909562498125	0.000240665580877315	0.00182269963582389
NAPSB	0.885874518871757	-0.121179964962622	0.000252412349821301	0.00188158612724967
CCDC74A	0.90983200766228	-0.0944953034777316	3.97569580246469e-05	0.000448113984212158
SERPINA5	0.955092447078813	-0.0459471399626485	0.00447360323042112	0.0180027579999213
AMOT	0.873980465932211	-0.134697253768349	0.00314687712805703	0.0137020829134588
DPYSL2	0.835009134603452	-0.180312614546267	6.46283626050373e-06	0.000110733965967506
RTRAF	0.872323449494908	-0.136594995530211	0.00572158933013774	0.0218764976669572
EVA1C	0.827039944295777	-0.189902284888133	0.000268456122624502	0.00197909387662848
IGF1	0.678932297136234	-0.387233866061593	1.16146390382047e-09	2.0630001960618e-07
SND1-IT1	1.11460853927954	0.108503257419071	0.0149638097137193	0.0459347937040333
PDK4	0.89666197284231	-0.109076329816416	0.000924101310063786	0.00520456461724019
RMND1	1.09317470908229	0.0890860400459252	0.0108955549634269	0.035815573463058
TNPO3	1.14189149503153	0.132686093612546	0.00773246011383806	0.0273933301556945
SSPN	0.805290235606824	-0.216552525416418	1.93070275188304e-07	7.31253667275701e-06
FCER1A	0.823888452185706	-0.193720131797529	1.71709643430451e-12	1.47412728885042e-09
SNX10	0.865437697344957	-0.144519891538104	0.000472530532830357	0.00309276337307391
PODXL2	1.12307742419337	0.116072617452235	0.000597086728731771	0.00371000451109451
NRXN2	0.780592641515237	-0.247701850983569	3.09621258882848e-06	6.35402033667549e-05
YAP1	0.874344089331298	-0.134281285917031	0.000714206874269502	0.00428955666147019
FRZB	0.840776494861721	-0.173429415493178	4.89583581085999e-07	1.43286649214431e-05
SPON1	0.912211064874065	-0.0918838849008125	0.000896754528821244	0.00510971524110423
ANXA2P1	0.911449551233519	-0.0927190333687685	0.00397022054642649	0.0165324220166878
LCN12	0.887034674506642	-0.119871205546387	0.0111961254420358	0.0365470482585083
PRAG1	0.879046443351823	-0.128917546115035	0.0001893182372411	0.00151425192551073
ADH1A	0.903331513286639	-0.101665668590369	1.95260426528289e-06	4.35405392661132e-05
GMFG	0.855498103037132	-0.156071403025658	0.00013380161405375	0.00113731371945688
FEN1	1.28754773965953	0.252739432215538	3.38497412963485e-09	4.35900043543727e-07
DLX5	1.15743246824986	0.146204162511548	0.00131915137445288	0.00694465696489218
IDNK	0.728411433712971	-0.316889234226935	2.27091008628294e-08	1.53913919137413e-06
SDHD	0.835023428896917	-0.180295495965733	3.4389571961954e-05	0.000407219965921897
EIF4E3	0.877752950076964	-0.13039010298571	0.0112740931249021	0.0367782480597661
RECK	0.778142018875479	-0.250846227921043	3.94261917254844e-07	1.20883519986887e-05
CEP131	1.13869033097705	0.129878769516247	0.0125957369774999	0.0399566534261087
RGS2	0.933204615646528	-0.0691307928049504	0.0136784672352466	0.0427535101509438
ZNF544	1.17446518408798	0.160812881499924	0.00288185698651791	0.0128604922803744
MUC20	1.10057414629409	0.0958319948116379	0.00357907361214528	0.0152098630795213
CRABP2	1.06216226082294	0.0603066991177214	0.0101339880424858	0.0338962158486004
CLDN11	0.798899658824775	-0.224519924550465	5.57538073668022e-10	1.38306786042944e-07
ZNF750	1.15165649456503	0.14120133600748	0.0020179945262406	0.00965152256700586
KPNA2	1.18534482426569	0.17003372319895	7.17586363845596e-05	0.000697273705984921
TMEM97	1.19939586975158	0.181817988150859	4.88552396776005e-07	1.43286649214431e-05
ENPP1	1.21635477090154	0.195858493378656	9.07053233543919e-05	0.000825482545226984

PBK	1.13617952347402	0.127671339040472	0.00457352046863515	0.0183332326334161
ESM1	1.18234235158882	0.167497514601043	5.37611774890488e-05	0.00056746685501248
GZMA	0.900957037215097	-0.104297705956754	0.000563569710501333	0.0035303755037826
ZBTB20	0.825471001453773	-0.191801144701999	8.9393155653856e-05	0.000817875923220271
ADAM15	1.12387157559815	0.116779488358442	0.0074110068574364	0.0264913923127376
GALC	0.84766033098221	-0.165275276520067	0.00182159643522973	0.00886866090535762
EEF1A1	0.909155921745458	-0.09523866844132	0.00127302231425313	0.00677410944289039
HBB	0.952259864025965	-0.0489173150254967	0.00998547165876839	0.0335519664150789
SH3KBP1	0.849607000230072	-0.162981389086231	0.000544522647228956	0.00344151675567651
ANGPT2	1.33237895778299	0.286966034495023	2.2616426808268e-07	8.14415678926524e-06
PELI1	0.828666966451157	-0.187936933812371	0.000851769714490855	0.00491868363154977
RNF144A	0.873586012577986	-0.135148685362826	0.00145465994831297	0.00750796933242498
TRAF3IP3	0.870130010288497	-0.139112641384906	0.00267302917277662	0.0120990977758984
HLA-DOB	0.876531152858426	-0.13178303291541	0.00172041049873878	0.00851280929779388
LONP2	1.13265358917277	0.124563188758156	0.00317284581166754	0.013803487141807
C6orf141	0.928113552490125	-0.0746011910845111	0.0082768473534137	0.028904434384701
MYL5	0.808298639220715	-0.212823685751469	7.5478087716886e-06	0.000125415364461187
GASK1B	0.914984093158678	-0.088848598383763	0.00110085180987734	0.00601324249488673
PAQR4	1.10175273547018	0.0969023075779753	0.00411668343141323	0.0169564360236058
HDGFL3	0.842900977820337	-0.170905791912536	0.00030997339000293	0.00221452556436212
CHKA	1.18012980606519	0.16562443756744	0.000490917123047318	0.00317677650856374
BRINP3	1.13918146967873	0.130309995460709	3.15121947840121e-06	6.4157832147212e-05
CCND2	0.793054488832067	-0.231863347435402	4.59270385459139e-09	5.25711501222227e-07
COL11A1	1.05809817842769	0.0564731253762571	0.00217231438025481	0.0102430534328149
DOCK8	0.842475546074734	-0.171410642607449	0.000829139760067884	0.00482888832508824
CXCL14	0.929627806168645	-0.0729709813604105	0.0002501347094387	0.00187002015721153
MSI2	1.20558174456792	0.186962226019024	0.000207192187678797	0.001629226251175
LGR6	0.844823333770775	-0.168627745944279	0.00329718828177546	0.0142341543466453
TUBB	1.13678473303909	0.128203867942	0.00735734427032143	0.0263727768520708
MAF	0.876429118290682	-0.131899446926085	0.00719207674170889	0.0259246936994699
OFD1	0.852610617376703	-0.159452321788495	0.00474503572935772	0.0188738834300553
F12	1.18318499433439	0.168209950060865	1.3667965288721e-07	5.96641433916965e-06
SLC7A2	0.922996001156423	-0.080130376930249	1.87511214993928e-07	7.31253667275701e-06
MST1	0.904659599013774	-0.100196539724666	0.0100229046653373	0.0336338644502623
CELSR2	0.887589045859214	-0.119246429303126	6.43162040960788e-05	0.000652151116730121
CAPN12	1.1204031529611	0.113688578538647	0.0157258158363538	0.0475094881953422
PSMB3	1.16470288178028	0.152466017391696	0.00117841362636805	0.00634938136968811
SLC9A2	1.19201440391648	0.175644652392699	0.000613789575454269	0.00378638335708376
MXD4	0.81416499206545	-0.20559224056394	0.000143340605454223	0.00120645009590638
PREX1	0.828556087980812	-0.188070746180252	1.82054191266051e-05	0.000249404558300912
FCRLA	0.908340398743509	-0.0961360821499335	0.00652677150714082	0.0240654259364942
RPS27A	0.843379495334116	-0.17033824987639	4.85566507390708e-05	0.000524451406044339
SF3B6	0.899832658645176	-0.105546467784545	0.00876420108226975	0.030254095040098
ZNF503	1.0947869620442	0.0905597891293718	0.0102273622037594	0.0341199110826196

MBP	0.869091806266708	-0.140306513420344	0.000883934510890686	0.00506218055549755
COL14A1	0.756192785686418	-0.279458927789779	6.94619061604135e-09	6.88073612754404e-07
ADCY3	0.73002517028309	-0.314676265594266	2.26848904507634e-08	1.53913919137413e-06
MMP7	0.950612017697253	-0.050649272659067	0.0022386138125938	0.01051148564145
LOC728392	0.811275724402496	-0.209147301879523	0.000382735416253572	0.00262453416947901
ME3	0.836092820208215	-0.179015643103499	9.20063100353812e-06	0.000144931040670412
GRINA	1.26345472401548	0.233849813421304	2.55118172036311e-07	8.76075802772692e-06
EMP3	0.8602853449122	-0.150491148357305	0.000829531324174387	0.00482888832508824
MBOAT7	1.12772194973772	0.120199624230656	0.0102197199460438	0.0341165116280438
ZWINT	1.28211947074744	0.248514545067114	7.23615197752308e-09	7.03272053513611e-07
PLIN1	0.878589589077862	-0.129437396955391	0.000717416199865089	0.00429698935523846
PXMP4	1.15056848612662	0.140256155993764	0.00230813886844629	0.0107594781098343
LMCD1	1.10602367132157	0.10077130551267	0.0105448461224469	0.0350109516948687
VWA1	1.15570919042722	0.144714173227932	0.00242321059201627	0.0111745369377581
TUBA1C	1.16958624906844	0.156650052991831	0.000395623492882032	0.00269201666028447
NACA4P	0.868818749469133	-0.140620749154651	0.00084440567988211	0.00488711646862106
CBX4	1.13993253451982	0.13096908040941	0.003747177287224	0.0158600741220138
OSCP1	0.874111962882244	-0.134546807546955	0.00849441013659648	0.0294728625078117
VPS37B	1.21481596946398	0.194592599858713	0.000215479237625697	0.00167663678702412
MRPS21	0.82550476266294	-0.191760246210445	7.30593488520125e-05	0.000702105794658053
KRT6A	1.05395440825982	0.0525491932631307	0.0152825774375725	0.0465802108762934
TMEM119	0.889456540328686	-0.117144631609846	5.87284167158038e-05	0.000611131463642637
GPAM	0.821101351750621	-0.19710872800079	0.000165679460465553	0.00134608028526508
NCCRP1	1.07381597778265	0.0712186385591123	0.00215242578948264	0.0101810332797292
SPP1	1.06044205574629	0.0586858549125013	0.00118738991380329	0.0063843898183724
KRT17	0.918620412182194	-0.0848822863682486	3.04089213991358e-06	6.26545416507794e-05
CARD10	0.870132233890218	-0.139110085906008	0.00213187315716221	0.0101116746156009
CRYZ	0.849296544309283	-0.163346867073775	0.000124484757209365	0.0010687016406424
CASP4.1	0.847508618218971	-0.1654542707893	0.000160522612083781	0.00131455003949691
LMO2	0.782662023172422	-0.245054319634142	1.57596370804111e-05	0.000223630552620379
PLCXD1	1.16055720273356	0.148900236972598	0.00154832864804369	0.00788866554507721
RBP7	0.820361289286028	-0.198010439067781	5.83617171813296e-09	6.26294177502143e-07
ARHGAP25	0.882625640288872	-0.124854131708844	0.00705709813098657	0.0255813599385727
KMT5B	1.15530257313441	0.14436227774785	0.00874735028348628	0.0302197191886236
BX648270	1.1272030314714	0.119739370980722	0.00333356879983025	0.014321278471998
HSPA1B	1.13623253229519	0.12771799327384	0.00184210774213114	0.00895160092426178
SNX7	0.827912735416312	-0.188847522160291	5.45249332689103e-05	0.000573179451567667
FICD	1.18691808624044	0.171360104181797	0.000910433835616348	0.00516539256953638
IQCN	0.859304192397011	-0.151632295823458	0.00174092462384463	0.00858879415640068
SBK1	1.10063882210315	0.0958907586023306	0.00549593043143593	0.0212853666558846
HNMT	0.832528435467398	-0.18328790099327	3.67887962234214e-06	7.2605015075419e-05
NTRK2	0.854896357494927	-0.15677503669721	6.71686381372648e-06	0.00011418668483335
SORBS3	0.81499121282238	-0.204577947612429	0.000144589274317452	0.0012129956873114
RPS7	0.880201541997998	-0.127604372734381	0.000250977257643411	0.00187359978858146

FUT8	0.857849543612652	-0.153326551965839	0.000383158550853856	0.00262453416947901
IFI16	0.885774584765832	-0.12129277974725	0.0016315063028128	0.00817498926633147
FURIN	1.14352204169656	0.134113009936864	0.00791705881293702	0.0279320342092045
MTMR11	0.858495761323055	-0.152573535962498	0.000382928522117619	0.00262453416947901
EIF4EBP1	1.21494488676329	0.19469871507503	0.000163717728354597	0.00133224331556797
H4C3	1.24438289740456	0.218639742299005	2.59708873959202e-07	8.85934046201226e-06
CXCR5	0.884789406448816	-0.122405621105795	0.00490686299517124	0.0193977369824459
CENPN	1.12779128088525	0.120261101278641	0.00107101988404896	0.00588147486432431
IL7R	0.905265339077765	-0.0995271859035679	0.000280905649019725	0.00205531959957472
TOP3A	1.19147058565605	0.175188330433677	0.000191450709879723	0.001526567502462
DCAKD	0.848707309203241	-0.164040899809302	0.00358174411094142	0.0152098630795213
RPL4	0.839368347141585	-0.175105637702884	8.93836344708198e-05	0.000817875923220271
CRIM1	0.899665987944248	-0.105731709047961	0.0130548002426741	0.0411577654352654
ARMCX2	0.87884856264053	-0.129142679824903	0.000122132107943595	0.00105554108727761
CLIC6	0.919809129839537	-0.0835890980291444	1.94809611766665e-09	2.86704088631455e-07
PLEKHA1	1.18029173646873	0.165761642215747	0.000788073652448267	0.00464458510727806
WDR4	1.13972519811356	0.130787179061802	0.0136328338243141	0.0426367498658421
RPL37	0.8009612244104	-0.22194274206131	2.27402584321707e-05	0.000297297134985054
RAMAC	0.876328888824005	-0.132013814644137	0.00858184090652997	0.029727681580051
OR7E156P	0.844177643463632	-0.169392328496096	0.00324370727562407	0.0140406186359156
OSBPL8	0.863361430931002	-0.14692186800633	0.000847931073378276	0.00490201229963131
SGK3	0.870819746927134	-0.138320273113661	3.25934583790946e-05	0.000393182445224159
PRUNE2	0.864407551212992	-0.145710918602056	0.0113385648854186	0.0369651567878426
TCEAL2	0.863105752586789	-0.147218054749436	0.00127864492990117	0.00679000003497004
CDR2L	1.19490781421431	0.178069039490913	0.000112478680790987	0.000983663301790109
OSR1	0.875574347587241	-0.132875210716407	4.81757277343673e-06	8.73778780139879e-05
CD79A	0.909239662670142	-0.0951465642529227	4.85659717886138e-05	0.000524451406044339
CCNE1	1.10149844965339	0.0966714797725649	0.00380411027609666	0.0160089640785735
TMEM91	0.843902754640624	-0.169718010644584	0.00029241257745297	0.00211844892610443
ADAP2	1.16447707586731	0.152272124332524	0.00284637306811098	0.0127603722139597
TOP2A	1.15657660074795	0.145464435470371	1.20857537530412e-07	5.42313877493139e-06
FN3KRP	1.15616354589393	0.145107235930233	0.0107901734711356	0.0356283227883459
P4HA2	1.25703555506755	0.228756214862453	7.1075222139359e-06	0.000119253573042293
FBXO5	1.22523138467744	0.203129711611049	4.22865555672233e-05	0.000469435447686999
IL6ST	0.918220810921042	-0.0853173825131205	0.00149437364607186	0.00768982882209404
BANK1	0.879678796450995	-0.128198442173032	0.0031376358261802	0.0136734028262726
SLURP1	1.1243477974596	0.11720313195326	0.00428188388473969	0.0174896840948372
NNAT	0.795860119077233	-0.228331838382516	0.000236795537519668	0.00180167476183724
RPL10A	0.888202212717292	-0.118555844889576	2.1788207792663e-05	0.000287344247074431
APMAP	1.15534662279423	0.144400405266211	0.00869846515594431	0.0301114207112024
CD300LG	0.78871365310282	-0.237351947833108	2.8048671726456e-07	9.50517816203783e-06
PDGFB	1.21107812305641	0.191510973684591	0.000296623607850254	0.00213959808840747
TUBA3E	0.793652585049034	-0.231109463804151	1.92656887315061e-07	7.31253667275701e-06
SLC39A4	1.1473186896554	0.137427645810907	0.000609844542462805	0.00376655783959941

TBC1D16	1.21460143092111	0.194415982581341	6.5042029016622e-05	0.000656924493067882
TMEM189	1.28659256951939	0.251997304693917	1.64634066685466e-07	6.89455347558403e-06
ERBB2	1.09240590122773	0.0883825126787407	7.41996558750164e-05	0.000707782272985573
ST13P4	0.846415034917686	-0.166745454664312	0.00213033537515737	0.0101116746156009
CLEC4A	0.814233415714145	-0.205508202593369	0.000297617658208959	0.00214110133719881
SET	1.1858718297788	0.170478225409147	0.00252359548306357	0.0115341972788469
HMMR	1.11257979849291	0.106681461468249	0.0118282612770519	0.0380083430056734
NMI	0.874317218765373	-0.13431201864475	0.000943654790675454	0.00530651291132016
ZFHX3	1.25270047563355	0.225301601554652	1.42233289201666e-05	0.000206378499345854
POLQ	1.279511484415	0.246478352282775	5.83693175626774e-08	3.3406706085039e-06
LINC00869	1.15034071507143	0.140058172469851	0.00924936780019377	0.0315102470494696
NDUFA4L2	1.11929706028794	0.112700863525232	0.00145729268533809	0.00751402865082735
SELE	0.809491856281091	-0.211348566099003	0.000711001723506813	0.00428347354126736
PRRT3	1.12230432087767	0.115384001052866	0.000781897874933595	0.00461875682773274
YBX2	1.10779762821247	0.102373925625654	0.0095070595577137	0.0323240024962266
ALDH3B2	1.07654135369299	0.073753452033808	4.69286123124331e-05	0.00050890375162388
PLSCR4	0.769045425021736	-0.262605240971741	2.9133573372306e-08	1.89470435606092e-06
LAMC1	0.839946205339026	-0.174417430458572	0.000145764203672325	0.00122086408636772
IGSF1	0.910414297735113	-0.0938555108398135	0.00244927846767957	0.011244414783438
KIF23	1.2751438746375	0.243059015097057	1.3650610600792e-06	3.33243105235449e-05
RRS1	1.17264726274047	0.159263810671646	0.00145439546604826	0.00750796933242498
H2AX	1.16557051554401	0.153210680060745	0.00091236724067582	0.00517008103049631
CERT1	1.18540064082285	0.170080810967748	0.000553208385274899	0.00348785360165361
PLXNA3	1.21896897525697	0.198005399196557	7.77907540823905e-06	0.000127806079910719
CD47	0.77040113032886	-0.26084395130192	7.20192202554262e-07	1.95247896597737e-05
RAD51AP1	1.11276277689214	0.106845911114488	0.00699013365686217	0.0253953991739192
BCL2	0.858621795296998	-0.152426738821343	2.10142862585286e-07	7.66690459970287e-06
P2RY2	1.20462657547228	0.186169623041344	0.000222355504918347	0.00171616756087346
TIMELESS	1.37073505987163	0.315347136006168	4.8636732727808e-09	5.4462567452378e-07
CRISPLD2	0.924434628167249	-0.0785729411285607	0.00737524988848602	0.0263848784106783
YWHAZ	1.23246628384322	0.20901727065239	6.80347427944582e-06	0.00011527860530732
RIPPLY3	1.12733933465547	0.119860285261414	0.000466369255015745	0.00306021405424981
OAF	0.877500282953867	-0.130678001187619	0.00207973737685593	0.00991001593726631
RDH16	1.17775770621202	0.163612381741132	1.24670992839529e-05	0.000186680315150121
TNFAIP8	0.86306768807628	-0.147262157514104	0.0101833789873433	0.0340393154859217
ACLY	1.17067483103461	0.157580361198844	0.00259281032844402	0.0118086348380329
G6PD	1.2000986867303	0.182403792354432	3.13879223915104e-06	6.4157832147212e-05
GPX7	0.88613373207954	-0.120887400625529	0.000787170738908752	0.0046445778649702
VAMP2	0.876740544230829	-0.1315441750433	0.014727451767495	0.0453443538878462
UQCRQ	1.14273092013084	0.133420941639521	0.00968049262873013	0.0327622979833042
WDR54	1.13253434320475	0.124457903040864	0.00413249935570472	0.0169963215764334
ELOVL5	0.906098744211534	-0.0986069896820954	0.00042395385771719	0.00283608613130032
NDRG2	0.842871569456451	-0.170940681985858	0.000147637819946408	0.00123201610749897
IFT122	1.13485059109352	0.126501004465287	0.000508551987080674	0.00327035116785587

RND3	0.915179463512642	-0.0886350979520733	0.00690018857328495	0.0252077101709154
PCSK1N	1.10656677112342	0.10126222312882	0.00293409910722125	0.0130064926861417
SAC3D1	1.22843331473384	0.205739629644851	7.52339742145715e-05	0.000713683611748173
SKAP1	0.917403912869887	-0.0862074316549689	0.0040495727613284	0.0167949672251229
GPI	1.34641685176006	0.297446879977797	1.1821154314031e-08	9.36781013408827e-07
DYSF	1.15244506980827	0.141885832981245	0.00638590828012187	0.0236557291007993
GSDMC	1.11374207708287	0.107725586047276	0.00638577231549345	0.0236557291007993
BIVM	0.859082787355556	-0.151889985209247	0.00298558109840066	0.013166719381731
EXT1	1.3034954570675	0.265049469205063	6.8567433540281e-07	1.90254544804759e-05
ETS2	0.866106261620485	-0.143747673997464	0.0053339572605738	0.0207516720915526
P4HA1	1.13398914758987	0.125741635233201	0.00567763733582911	0.0217931497128207
SIK3	0.786805702157546	-0.239773945216491	1.23124577384493e-06	3.06383912129239e-05
RNASEH2A	1.28772590984021	0.25287780211732	3.80720309295448e-06	7.45661716038347e-05
GGT2	1.1332046622189	0.12504960317234	0.00514172801156978	0.020106535338485
MAP3K1	0.879880465980394	-0.127969214849362	0.00133810920461757	0.00702609634351183
ARMCX1	0.880321968151354	-0.127467565525787	0.000107077917403	0.000942834790671545
REXO2	0.872943033936239	-0.135884978494713	0.00431675069813632	0.0176053704244657
PRKCD	1.22670862249219	0.20433466604645	2.34486428967232e-05	0.000305009998891468
BAG4	1.16557443211806	0.153214040275689	0.000407814750934512	0.00276038604738985
PLAAT2	1.17948095003397	0.165074468852559	4.05637234740916e-05	0.000455215118987028
SOX13	1.17917353724735	0.164813800925132	0.00120645666671473	0.00646665795030964
HPGD	0.89881891332271	-0.106673696032203	0.00201691661714224	0.00965152256700586
RECQL4	1.24388281496191	0.218237789688483	3.97433820196398e-06	7.6961714580137e-05
DNAJC10	0.850069314239459	-0.162437386658414	0.00449328243507837	0.0180678359274697
WLS	0.894353512272029	-0.111654154372164	8.81368561276577e-05	0.000809256588081221
IL27RA	0.794459746219467	-0.230092959840241	2.83713060497461e-09	3.9497458773579e-07
SQSTM1	1.1591334900362	0.147672734633115	0.00110337874362623	0.0060206609199351
HID1	1.17405009466714	0.16045939056979	3.47257072534454e-05	0.000410167495395347
NABP1	0.868493499542493	-0.140995178023872	0.00308114651083448	0.0134728231556099
UBE2C	1.16292685768376	0.150939980487229	7.37430197290541e-10	1.51940117849743e-07
CLEC3B	0.798212778875928	-0.225380076879109	3.19215763656427e-05	0.000386957521709619
COMMD6	0.857007028615264	-0.154309158999945	6.30456831732544e-05	0.000644341892907606
CCDC151	0.887752738688645	-0.119062022186345	0.00633379824524317	0.0235614740170838
FOS	0.930839342823301	-0.0716685806955366	8.66617114729245e-05	0.000798082426938499
DUXAP9.1	1.14225111396623	0.133000976699315	0.00249886020134725	0.0114414479085686
PHB	1.12160074938868	0.114756905440513	0.0166824028173437	0.0498440005290819
P3H2	0.83568650249697	-0.179501733239592	0.000411199454144577	0.00276931132380213
SELENOP	0.939038252464466	-0.0628990631547759	0.00725212511079964	0.0260499975214288
LAMP5	0.909425688215198	-0.0949419905575875	0.00187922383629975	0.00908909106176525
AFDN	1.12915933880386	0.121473407896451	0.00106085059025757	0.00584432234269172
MSRB3	0.880766881523502	-0.126962294730738	0.00294110386747724	0.0130263336383278
SCRIB	1.13499669194131	0.126629736339962	0.00567763034811136	0.0217931497128207
TMC6	1.17445298556213	0.160802494994329	0.000329380708628762	0.00232122630747902
RPLP2	0.855892604115413	-0.155610373193464	0.00012940320963446	0.00110356942521043

TMEM241	1.16677851306752	0.154246543575775	3.62134645344093e-05	0.000418241156539781
HEPACAM2	0.880759253536596	-0.126970955387829	0.00239667345223749	0.0110918822573902
TMEM243	0.798962336960462	-0.224441472048454	6.86136826628667e-06	0.000115878386687353
FMO2	0.841661238640838	-0.172377675101181	2.54697686205774e-05	0.000325545355247132
ABCC11	1.07313172172016	0.0705812163379586	0.000656365907367588	0.00401536910789839
EYA2	1.09983914714753	0.09516393924581	0.00175312070285279	0.00863319764856091
LAMC2	0.899979991552184	-0.105382747513636	0.00123406865961721	0.0066009217712235
CPZ	0.854795083132401	-0.156893507621538	0.00150176903411093	0.00771247487009511
TOB1	1.14717718915556	0.137304306735576	0.000600672273530386	0.0037267011312542
GSE1	1.14907056616939	0.138953412271805	0.00243970838734675	0.0112204802707349
TACC3	1.2111829830982	0.19159755398065	5.92019894968555e-05	0.000612348288952415
VSTM2L	1.24757063560341	0.221198168766768	3.48781216448494e-05	0.000410176266193195
PTPN1	1.11656842331612	0.110260074178585	0.00781975321902994	0.0276645252961698
LRRC17	0.897349593633669	-0.108309756358496	3.57188699251742e-05	0.00041643292163505
PGM5	0.861688565029132	-0.148861367070339	6.24340545304614e-05	0.000639359472935202
PRAME	1.13620025942688	0.127689589469398	2.87622973707695e-06	6.02254446165992e-05
DHCR24	1.12909656218341	0.12141781045912	0.00115703450423761	0.00624725862822632
GALNT6	1.17880745882175	0.164503299331484	1.22386768926511e-07	5.43460557534877e-06
ARMCX6	0.903494046944376	-0.101485757854376	0.0101325779630582	0.0338962158486004
CKAP4	1.13194340775502	0.123935985382015	0.0159349106012996	0.0480465999894803
BC132896	1.10869016295148	0.103179285159599	0.0155845599122533	0.0472212165341275
PLPP1	0.893592951155197	-0.112504919401501	0.00669481540562839	0.0245619616484272
RMI2	1.23265349942078	0.209169162320448	4.56644255732391e-06	8.43073319454318e-05
SLC19A3	0.872410919832751	-0.136494727730763	0.00631153867861061	0.0235074011088382
MBOAT2	1.28281274667061	0.249055125387597	6.90645332459635e-08	3.55751410749958e-06
CXCL12	0.87029517650683	-0.138922841535901	1.7001660033689e-08	1.21632709491016e-06
PRKCQ-AS1	0.823895250005026	-0.193711880933886	7.76028173248833e-05	0.000732110095312224
PDLIM7	1.32289539957685	0.279822818951125	1.69119251267107e-07	6.96906610621496e-06
CD44	0.895833967410016	-0.11000018740805	0.00264673847764068	0.0119906331559606
SLIT2	0.814225430234531	-0.205518010000703	3.22971754611035e-06	6.54971459843087e-05
HROB	1.25224588871311	0.224938650130307	3.28633549934027e-06	6.61246646761786e-05
LEF1	0.877023993788551	-0.131220928046288	0.000449031701686235	0.00298446747791716
HTATSF1P2	1.12076902340225	0.114015077723542	0.00211926498182818	0.0100704187466761
IMPDH2	0.863986463810535	-0.146198177186763	0.00279506412434864	0.0125631547159859
CCNB1	1.22386125429636	0.202010823332005	1.87674689000381e-05	0.000256422366854366
OSBPL6	1.18325591973509	0.168269892737057	0.000568413470844874	0.00354896701614781
EVI2A	0.860055938603399	-0.150757846962237	0.000319005187704325	0.00226335498879474
CNOT7	0.860364420937327	-0.150399234213297	0.00460772681403644	0.0184416478780899
ANKRD35	0.850839141852404	-0.16153219077588	4.91359022182876e-05	0.000526193414399999
PNMA2	0.877828984204811	-0.130303483128887	0.00583339735336995	0.0221309630752321
C8orf34	0.843375509976788	-0.170342975349048	0.000750660520784102	0.0044546685974181
OMD	0.814207307891178	-0.205540267405543	2.49012643334294e-09	3.56295590504152e-07
MEIS3P1	0.894245608418804	-0.111774811764989	0.0108567770465173	0.0357376901778206
ACTG2	0.928763718520121	-0.073900912108926	6.82431762066308e-05	0.000673411112337845

TLE1	1.14221550149617	0.132969798769361	0.00308952875742564	0.0134943027359931
UBE2S	1.22224498386038	0.200689318447235	1.01717836588675e-08	8.58932092243055e-07
OSTC	0.873632152982357	-0.135095869514993	0.000451832639109851	0.00299535382761241
CHAF1B	1.14634670178435	0.136580104636977	0.00778456320662697	0.0275589588160381
C17orf97	0.90791818272569	-0.0966010115710328	0.00906753928702593	0.0311057056726251
MLLT6	1.11354263719878	0.107546498135125	0.00916988986131121	0.0313639459997437
FHL1	0.904679588741421	-0.100174443560738	0.00743808331225385	0.0265511492477843
CDO1	0.795811188545005	-0.228393321594544	3.34529226131423e-06	6.67891489846109e-05
CNN3	0.885750770727474	-0.12131966509584	0.0038775967983375	0.0162518316584511
RGS5	0.849504441170779	-0.163102109901734	2.78078829208576e-06	5.87042643136628e-05
KIAA0562	1.15439648477396	0.143577683405285	0.00514471240072282	0.020106535338485
SLC27A2	0.922147054037921	-0.0810505735253223	0.00140151066406897	0.00729947566291129
SYF2	0.842942922308806	-0.170856031091295	0.000153337692450023	0.00126577316315716
FBLN5	0.852918842937115	-0.15909087911519	0.000232749996366282	0.00178141936297581
SEPTIN4	0.870180640937738	-0.139054455624543	0.00588882427303086	0.0222875340414269
TK1	1.23660091649929	0.21236641928656	2.61894736053003e-10	7.93541050240599e-08
IGLL1	0.931247881884822	-0.0712297837770517	6.86999521135415e-07	1.90254544804759e-05
CCNE2	1.26585641825954	0.235748903593121	3.2023511859978e-09	4.22956691258324e-07
HNRNPA1	0.907725033717607	-0.0968137725441823	0.00285589890042698	0.0127919436835647
ZMYND8	1.27367033771001	0.241902762056316	6.42385082314315e-06	0.000110666406655553
CDC25B	1.11544862804753	0.109256680981516	0.00191132644612591	0.00922703141892649
PDXDC1.1	1.24604306106666	0.219972979211644	6.91181441586671e-05	0.000679441909468119
TROAP	1.30014731122543	0.262477574374933	3.84748954773231e-11	1.80167442366992e-08
CENPF	1.21451397173868	0.194343973501519	7.45903701986298e-08	3.77007780530958e-06
LRP8	1.1251101868339	0.117880974712551	0.0041181326859893	0.0169564360236058
RPL29	1.11708769340553	0.110725024978198	0.0132856353399325	0.0417028078220551
UNC93B1	1.17061689076417	0.157530866921891	0.00161953104814388	0.00813080353702643
ERO1A	1.20613237684036	0.187418857488826	5.03431529715715e-05	0.000538003279992873
PHLDA2	1.25920939696991	0.230484061303975	2.14863165893369e-06	4.66987412454322e-05
GABRP	0.942623731095227	-0.0590880885997608	0.00109677012575051	0.0059973067067313
SOSTDC1	0.853368831224994	-0.158563431950067	6.74998624112365e-05	0.000669265911136912
CST7	0.889610038588727	-0.116972071164677	0.00846827918070256	0.0294134228319615
PCOLCE2	0.907198565796696	-0.0973939269218301	0.00505906430933711	0.019907746567911
KCNK1	1.07439087230365	0.0717538705687333	0.00278868007008121	0.0125454070226972
RIMS4	1.12029037662885	0.113587916550868	0.00845782603810655	0.0293969378692894
LDB2	0.799829701642268	-0.223356446922037	2.07992831483904e-06	4.5785088674085e-05
KRT80	1.1243595349545	0.117213571279649	8.2884055635106e-05	0.000769253640678255
CDCA8	1.20479389394139	0.186308509940912	5.23222280025431e-06	9.45655426109121e-05
FBLN1	0.877768819346436	-0.130372023723181	7.32574961270918e-07	1.97565111283063e-05
ABCC8	0.933596023828489	-0.0687114569857748	0.0106070944797557	0.0351410083716425
ZNF264	1.16420045126816	0.152034543476242	0.000903619327231909	0.00513746485052049
ARHGAP39	1.19218639545124	0.175788928441183	1.47123122802056e-05	0.000211684694288656
TRIM46	1.13104376183778	0.123140889433862	0.00616859489750731	0.0231423396336928
CNN1	0.855003995907228	-0.156649136480592	0.000492224974111064	0.00318124321411053

STX1A	1.23043810010976	0.207370284918688	4.53675220531722e-06	8.40604698186655e-05
RNFT2	1.14518047296388	0.13556224289481	0.00411121997861033	0.0169550793513385
SLC35A2	1.21714047245503	0.196504232533432	0.000295667828507223	0.00213669164728546
DDX39A	1.12758981652701	0.120082449112932	0.0150713139291672	0.0461041817572321
ARGLU1	0.867330803485102	-0.142334825394	0.00510920057640658	0.020043786876672
KLRG1	0.844254316666314	-0.169301506710504	0.0043343308649465	0.0176278645957003
RERG	0.945684483167047	-0.0558462928704227	0.0148692250654603	0.0456989130740967
KRT18P55	1.07885918910538	0.0759041764674547	0.00770650402784319	0.0273201667222438
JUP	1.18133377844815	0.166644120864849	0.000217526371679215	0.00169001257996928
IDH2	1.15403195615148	0.143261859341568	4.36318536550461e-05	0.000481258411514224
SYNCRIP	1.14495666226934	0.13536678674346	0.0159804104551762	0.0481093478986632
GIMAP4	0.888268181052786	-0.118481575894432	0.00125229822370433	0.00669148148371473
GADD45A	0.825431944510444	-0.191848460558368	4.10844878406153e-05	0.000458678348072957
NUFIP2	1.20826263164416	0.189183486183885	0.000315874187964812	0.0022442316444231
RAP1GAP	1.07328556398151	0.0707245642905184	0.0149310778255476	0.0458616469167537
LY6E	1.08118463275243	0.0780573221785953	0.00812231991574223	0.0284806466208225
TSHZ2	0.839168823102569	-0.175343373325892	2.14736408801904e-06	4.66987412454322e-05
TMEM54	1.16326699395821	0.151232420669402	0.00185619996126618	0.00901157964230168
ABCD1	1.17982466879571	0.165365841671268	0.00106512485009557	0.00586160053722467
SRPX	0.915915831102764	-0.0878308059723136	0.00131624157476111	0.00694465696489218
FBXL20	1.18761745535475	0.171949161807829	1.71018006491713e-05	0.000237443059687012
C2orf88	0.83604387286223	-0.179074187774161	0.00408151435575072	0.0168595673187426
MORF4L1	0.909761200285751	-0.0945731311770832	0.0117873479678158	0.0379478933638871
KRT14	0.887134291925514	-0.119758907990899	2.03473993043197e-07	7.59488795772109e-06
ABCA12	1.18814264831872	0.17239128807446	0.00125546997733189	0.00670147756812081
SFMBT2	1.15462982944782	0.143779798620008	0.00208444397886869	0.00992326334117617
RPL21	0.893916598245111	-0.112142798714024	2.82438826116266e-05	0.000354839608128021
ELMO3	1.16935619376732	0.156453335622783	0.00176014233369396	0.00865949681075225
ALDH1A3	0.921423987844701	-0.0818349926921121	0.0111601619427623	0.0364758846238381
C7	0.840553888195451	-0.173694213738441	0.000290270605973045	0.00211410652967296
MB	1.10474518800175	0.0996147092842147	0.00217349202136802	0.0102430534328149
MTERF3	1.14757476040248	0.137650811507889	0.0127548649665697	0.0404060943682661
PUF60	1.26127054825189	0.232119584526719	1.50845747171881e-06	3.5642497416622e-05
ELOC	1.14031241150407	0.131302270041893	0.00940865949577548	0.0320105713756536
SALL4	1.17285849655555	0.159443928597009	7.96384766849742e-05	0.000747239909326033
SPARC	0.923460682760789	-0.0796270544118016	0.00844024067063378	0.0293556243716642
DCLK1	0.908838940469287	-0.0955873837055606	0.000479799060873095	0.00312841134500926
RPL15	0.904128632088874	-0.100783636578816	0.00180321570520699	0.00881248965609224
TBC1D10C	0.906221222684197	-0.098471827596937	0.00623586619079043	0.0232760483686677
TESC	0.822029343668917	-0.195979186669053	0.00120049355465457	0.00644139822919342
BIRC3	0.899883202659359	-0.105490298902242	0.00169352784699327	0.00840772943447104
SERPINE2	0.913893612767649	-0.0900411117122651	0.00128176715027789	0.00679957012469766
DOP1B	1.21712251918126	0.196489482053445	3.45637644444957e-06	6.87405214878753e-05
ACTN1	1.12785418526611	0.120316876338879	0.00545314981347327	0.0211833896600308

ZBTB16	0.880979516493283	-0.126720903607643	6.94570184174779e-05	0.000680176999749864
MAP3K6	0.882866248689098	-0.124581563589764	0.013200547375334	0.0415107973817115
TNPO1	1.16531958720086	0.152995373175335	0.00383227843139259	0.0160880735127166
MRPL12	1.21319629352015	0.193258441700807	0.000336800989155813	0.00235966350332135
BARD1	1.18289941341217	0.167968554679812	0.00104833879902681	0.00578777401263353
KIFC1	1.18902993218557	0.173137791643733	5.64365628969448e-06	0.00010093914426464
MAFA	1.09167261408354	0.0877110284197249	0.00452374615196599	0.0181761438602003
CD24	1.06174830879816	0.0599168973662349	0.00248565555573926	0.0114012571394594
HSPA4	1.1907199433331	0.174558118585666	0.00189058239006573	0.00913545017938891
LRIG1	0.920662712106157	-0.0826615290130185	0.0131125368013924	0.0412852549290784
PRKCB	0.90358216695892	-0.101388230130935	0.00152375721048204	0.00777886361862536
PCLAF	1.17813473737006	0.16393245676178	7.01889219977869e-07	1.92310179367341e-05
SOX2	1.14071042980345	0.131651252345981	0.00665892839715925	0.0244477121694706
CCDC25	0.784735148864889	-0.242409008109566	2.92605926488499e-05	0.000364061141870111
S100A13	0.86292596254516	-0.147426382372508	9.02886979596213e-05	0.000823436553152716
HHEX	0.850071795795418	-0.162434467423128	0.00610765495771827	0.0229638910125597
TUFT1	1.15620903454756	0.145146579636366	0.000982488610313339	0.00549489558276222
CDHR3	0.910902036973995	-0.0933199210125588	0.00782642199556138	0.0276691144125852
RPL36AL	0.879579807240333	-0.128310977317733	0.0108134770427257	0.0356595520147761
UQCRC2	0.892862656976106	-0.113322509512834	0.00697626462390685	0.0253776405916272
ACSS1	1.1105578655484	0.104862470647556	0.0160946150423613	0.0484248610299084
TH	1.16903251874576	0.156176499678528	0.000623230172060394	0.00384002226828121
GSR	1.13527276589971	0.126872944439172	0.0127809091532926	0.0404388593664682
FLNB	0.916155202974515	-0.087569493098893	0.0123516437822094	0.0393337032175497
CDKN3	1.18980306900038	0.173787805193773	7.81030295231738e-06	0.000127806079910719
OPTN	0.78985188584314	-0.235909837373325	2.38343951671607e-05	0.000308469772628253
CDCP1	1.15347104870802	0.142775699667387	0.00291061304174025	0.0129469497219378
ST3GAL5	0.840502547827786	-0.173755294814104	0.00116272193048156	0.00627139336535134
ADAM17	1.18577382970206	0.17039558230738	0.000924514972777087	0.00520456461724019
HMGCS1	1.23886575969371	0.214196251090106	2.19498800580568e-05	0.000288428143313904
PPP2R3C	0.883394071119897	-0.123983891275449	0.0137313557826397	0.0428927917746374
CDC42	0.885917526998566	-0.121131417361432	0.0101226324772143	0.0338962158486004
FNBP1	0.841188253913252	-0.172939798739846	0.000488648135761774	0.00316673402652242
FCMR	0.897499011510518	-0.108143260001348	0.000795040937215523	0.00468029242011104
FGD3	0.897901743644438	-0.107694633543419	2.37912624636089e-05	0.000308469772628253
ACTB	1.09602092060918	0.0916862764907105	0.0116088493563092	0.037537465809384
SLC30A8	1.05493715300237	0.0534811945397625	0.00797559767749103	0.0281193043372733
PPP1R14B	1.22274615073457	0.201099272392765	9.92334708339165e-05	0.000881295876319834
ATP6V0A4	1.09364548169403	0.0895165945295501	0.0120256167556794	0.0384745042909967
SGCE	0.894832303716806	-0.111118948412081	1.95811553435477e-05	0.000265809401268848
NME5	0.88006343591452	-0.127761287841475	0.000321463689897183	0.00227453223442361
SMARCA2	0.768675275232843	-0.26308666753507	3.24211568323355e-07	1.02454833646233e-05
EIF3F	0.866288439348079	-0.143537355034743	0.00332366966716327	0.0142983081887979
SLC35B1	1.22901502523875	0.206213056089919	6.18486734676328e-05	0.000635893247568416

CITED1	0.841326908140209	-0.172774980935648	1.20991351689847e-05	0.000181698674214112
LTB	0.926031155359763	-0.0768473998050054	0.000809633181659936	0.00475532556297643
SETBP1	0.88658142602358	-0.120382306559501	0.000460431407993346	0.00302946259372099
TMTC1	0.884689869931749	-0.122518124844608	0.0132083472934625	0.0415107973817115
TLE4	0.787825874990146	-0.238478184366595	1.59150622506454e-05	0.000225215619926029
AKR1B10	1.11166299748563	0.105857090081401	0.00163806841600436	0.00819989349935708
GOLGA2P7	1.14137125327718	0.132230393309342	0.00138400849018897	0.00723025125046998
PSIP1	0.816067656353294	-0.203258015257392	0.00047041138227382	0.00308281047085553
UBE2E3	0.898299558744595	-0.107251681958724	0.000408424304353958	0.00276088397864467
CRIP2	1.11824632387549	0.111761675934838	0.00199966139131422	0.00958163332712516
E2F2	1.13640244372843	0.127867521407792	0.000153705474645283	0.00126677903983657
TMEM26	0.857157587035723	-0.154133495099907	2.4341048933519e-07	8.61276068825106e-06
KRCC1	0.74641668270505	-0.29247127880777	2.52926868695462e-07	8.74380067550554e-06
DYNLRB2	0.868810015167395	-0.140630802282128	0.000147813231301566	0.00123201610749897
CCNB2	1.16226885333512	0.150374002868148	8.32036070010825e-07	2.20918443125039e-05
GIMAP5	0.90718873441109	-0.0974047640629468	0.00919013898131824	0.0313915158440121
SNCA	0.843997343516508	-0.169605931883422	0.00156840900756445	0.007964542466093
NQO1	1.11139142445692	0.105612765851299	5.1227944145951e-05	0.000544072454218131
KIF15	1.28755747106721	0.252746990281627	8.47907875377195e-07	2.23978126465022e-05
RPS6KA2	0.813662432650035	-0.206209700896346	4.32065666236819e-05	0.000478617257373302
OPRPN	0.857960626604968	-0.153197070284941	7.80659622966117e-06	0.000127806079910719
VCX3A	1.1203887109849	0.113675688473813	0.0056361841854455	0.0217142743000971
PLAU	1.07588002734556	0.0731389567736632	0.0166947787209342	0.0498520609805984
CCNG2	0.880676706095388	-0.127064682827441	0.0020332873100277	0.00971564279587448
COL16A1	0.870792809520608	-0.138351206981734	7.21504339526234e-05	0.000697273705984921
TRIM3	1.15000719576014	0.139768199538309	0.00895504165437374	0.0308132395201597
RASSF2	0.835492296560551	-0.17973415114413	3.19271882598695e-05	0.000386957521709619
CORO1A	0.911101606357159	-0.0931008551674467	0.0144500937241229	0.0447848572641137
COX7A1	0.903597368382288	-0.101371406762729	0.00168339287944771	0.00838603164606879
TMED9	1.15518560418916	0.144261027331167	0.00346959586985025	0.0148306305554725
TMEM98	0.924775335285433	-0.0782044517425281	0.0109528437967783	0.0359350945205128
DKK3	0.924450359835417	-0.0785559236631479	0.0123679041790739	0.0393337032175497
FAM107A	0.888701089098532	-0.117994332742192	0.00179054463186889	0.00876720094938846
PPEF1	1.16374994446358	0.151647501887185	0.000662589978280056	0.00404863698472191
UPF2	1.16131019638468	0.149548847378006	0.0048873233225622	0.0193529124176787
CPE	0.875007736829541	-0.13352255057271	1.17224672838165e-05	0.000176556809879938
PIK3IP1	0.794565443211287	-0.229959926087624	5.97919674874949e-05	0.000617211271599372
VCX	1.14469876518015	0.135141515213794	0.000243403976523111	0.00183568650522774
AURKA	1.209553544268	0.190251319842512	1.36807910528783e-08	1.03631992225553e-06
SRC	1.13129612739818	0.123363990801298	0.0150726930846785	0.0461041817572321
CNKSR3	0.926498545853653	-0.0763428027629156	0.00642730306226029	0.0237496686324984
ROPN1	0.916610744018159	-0.0870723853762484	0.0013687112921038	0.0071575958026667
TGFBR2	0.89265156058466	-0.113558963964279	0.000402439186000064	0.00273117819115458
UTP3	0.868366956703986	-0.141140892480409	0.014046514088936	0.0437446155212271

FAM102B	0.849108298842884	-0.163568540331396	0.000688731922628341	0.00417863148817266
PPP1R16B	0.86819312128514	-0.141341099109168	0.000992572355358348	0.00552728670535227
IFT46	0.849285366424689	-0.163360028505112	0.00299022232247328	0.013175906914508
CD1E	0.76383936419396	-0.269397768213197	6.26311123131965e-09	6.4522571905055e-07
CEBPD	0.905332015423335	-0.0994535346906859	0.00737608715033522	0.0263848784106783
ATL1	0.882304656700854	-0.125217866857031	0.0115084952265236	0.0374244058786762
ADGRG1	1.25204256934608	0.224776273175031	4.31249233560127e-09	5.04855636833685e-07
DNAAF1	0.833475346551934	-0.182151155450768	5.06803631732171e-05	0.00053977315094825
LHX2	1.19787352425044	0.180547921708115	0.000211763454204384	0.00165271750394967
NOP56	1.23717562787611	0.212831062213559	3.15443701081632e-05	0.00038503566451931
ICAM3	0.850634997641292	-0.161772152356668	0.00512395593481786	0.0200863752056673
ENTPD1	0.843702428255582	-0.169955419711602	0.00140349407777441	0.00730242221678381
TMED3	1.23617309853722	0.212020396589991	9.65076343142075e-05	0.000863039625611949
DCN	0.933306671933446	-0.0690214376805551	9.03206469678285e-05	0.000823436553152716
C3	0.894134051458678	-0.111899569346137	4.63844431402e-05	0.00050441380412696
SLC51A	0.893051955172401	-0.113110519313845	0.0111231630344018	0.0363855212127625
LPL	0.886143763025261	-0.120876080789049	0.000515434102221646	0.00329813796340832
SRM	1.13229827468982	0.12424943861491	0.0152110383064822	0.0464170961591764
QPCT	0.900275447061995	-0.10505451019117	0.00224648280720555	0.0105179496515431
VCX3B	1.12956795148038	0.121835215789541	0.000892745290300795	0.00509489468460452
BRWD1	1.22465095352184	0.202655867494209	4.87622487954283e-05	0.000525469337960777
GLS	0.839881021971171	-0.174495037687252	0.00224815813751679	0.0105179496515431
SMIM30	0.861572173664917	-0.148996449779274	0.00228378746558095	0.0106556061913111
RNPEP	1.2564490875367	0.228289557910146	6.03584913454448e-05	0.000621813177840772
TM7SF2	1.11674638987427	0.110419448537699	0.000917203103443356	0.00519177273168871
RPS27L	0.88903163963794	-0.117622453957632	0.0115539619943114	0.0374776185344446
TCIM	0.900088579302002	-0.105262099054231	1.6764965822218e-06	3.86767877831411e-05
FBLN7	0.743153754382206	-0.296852318415596	1.5101478702835e-07	6.42873692547961e-06
TUBG1	1.14365251419268	0.134227100482278	0.00876904754316955	0.030254095040098
SEMA3C	0.884423351998777	-0.12281942600689	0.00159339953202981	0.00802307037095359
ADM	1.06143538002235	0.0596221242105039	0.0152259829445479	0.046435191324669
GCHFR	1.11460039684954	0.108495952199836	0.0111004135067391	0.0363729198302881
MATK	0.883821548245532	-0.123500105223211	0.0033861905116828	0.0145231201712557
MME	0.886095445761728	-0.120930607585776	0.00222497265744919	0.0104612402402665
FAM171A1	0.891342316110443	-0.115026732129144	0.00063208778369926	0.0038853033100655
CERS6	1.13620774263186	0.127696175614997	0.00162946188539107	0.0081726953959585
PDGFD	0.777313159583762	-0.251911973024661	4.94756797050402e-07	1.43982613650091e-05
REPS2	1.12613819394468	0.118794252155422	4.04692346721514e-06	7.80735025887016e-05
GPRC5A	1.24630827407571	0.2201858007399	1.30975632143162e-09	2.11592032216888e-07
MCM6	1.12707626336897	0.119626902128394	0.0159241271754253	0.0480465999894803
C11orf80	1.23655270249238	0.212327429385937	1.89284006433784e-07	7.31253667275701e-06
GREB1	0.839117269532715	-0.175404809299291	9.88727296590908e-05	0.000879608688210667
ALPL	0.921232317633286	-0.0820430295479668	0.00581522549471873	0.022106440238595
BAIAP2L1	1.16545301204481	0.153109862975108	0.00012413016252146	0.00106743650609022

TRIP13	1.17565644942687	0.16182667195564	1.67621219088068e-06	3.86767877831411e-05
WIPF1	0.824430159717711	-0.193062846794987	2.13239441833204e-05	0.000283091846619287
SPRED1	0.883284304928839	-0.12410815406452	0.0153623083720469	0.0467955354372642
DCBLD1	1.26347675183664	0.233867247864122	4.63009222838891e-06	8.48740393894351e-05
ZNF562	1.24478515858706	0.218962951642008	2.60826479827258e-05	0.000332553761779754
AQP7P1	0.844545542977891	-0.168956615262532	0.00375232818796318	0.0158688362037753
CHST1	1.08373159824454	0.0804102692633079	0.00397929081108674	0.0165568069207656
DNAJC12	0.952716505287885	-0.0484378956709862	0.00419777606048562	0.017188191166583
SFXN2	0.864254209764623	-0.145888329188942	0.0030187672903495	0.0132676367854866
DYNC2LI1	0.898821997689456	-0.106670264460605	0.0158674081738914	0.0479091556293754
TMEM176A	0.893128333080148	-0.113024998374782	0.00288782302974039	0.0128604922803744
IRAK3	0.838314501092679	-0.176361949274078	0.000125257737372227	0.00107354842796063
ANKRD36.1	1.14608014306423	0.136347548713175	0.00722185168033673	0.0259593565983353
FTL	1.17620378151415	0.162292118065036	0.00306555898503515	0.0134160529582974
TPSAB1	0.935026990916081	-0.0671798828201759	0.001592713597531	0.00802307037095359
GMPS	1.24640362186097	0.220262301987768	1.28931407591567e-05	0.000191943838296
MATN2	0.922743578279052	-0.0804038964253683	0.00570948507010913	0.0218632155019871
CDS1	1.24460532744304	0.218818473589776	1.30077800352663e-06	3.19062261722174e-05
KRT5	0.903937389572373	-0.100995180311095	0.000207803408412652	0.001629226251175
CANT1	1.18224994309492	0.167419354408983	0.000114058468055456	0.000994103500767608
NEK6	1.17822529918727	0.164009322619158	0.0027380499013491	0.0123510262842797
NBPF22P	0.830664884854253	-0.185528832784225	0.00115643395160624	0.00624725862822632
SPINT1	1.28529892870503	0.250991320635613	4.29606797575526e-06	8.07629421281581e-05
PIMREG	1.1891319530277	0.173223589707209	0.000176951707004131	0.00142418475434106
SLC2A8	1.17441762305634	0.16077238477399	0.00242200551800977	0.0111745369377581
TSHZ3	0.856612649820152	-0.154769446433659	0.00127247009460115	0.00677410944289039
KDM5B	1.14142332649547	0.132276015652026	0.00956298842234743	0.0324712942409437
RAB33B	0.871966468469724	-0.137004309402282	0.00791225636079277	0.0279320342092045
TTK	1.17715450305774	0.163100088191424	4.27341764672343e-06	8.06314076859794e-05
GNG12	0.895052473712563	-0.110872932579505	0.0097740328922533	0.0330137989691782
TRAPPC9	1.29255096337224	0.256617756698335	2.05092438988027e-06	4.53403928423746e-05
CPXM2	0.903174654610871	-0.101839328308724	0.00318240178330789	0.01383337686567
MTPN	0.882886434962904	-0.124558699375546	0.00977120536572666	0.0330137989691782
CDKN1C	0.786607339044767	-0.240026088943072	1.44203822283142e-06	3.43886059527993e-05
PDE1A	0.785380416172976	-0.241587071993802	5.98134667274027e-06	0.000105876002444279
GRB7	1.1226525309682	0.11569421645236	5.73787261579756e-07	1.63291612397642e-05
TAOK1	1.18928458956022	0.173351941095617	6.35938709759862e-05	0.000648657483955059
HOXC13	1.16646364151234	0.153976643122895	7.17565066080457e-06	0.000120005768031832
ARFGAP1	1.21221567827587	0.192449824188308	7.40980810550591e-05	0.000707782272985573
GNL3L	1.20954576926377	0.190244891826774	3.87590652164238e-06	7.56242215643177e-05
GGTLC2	1.12788991137779	0.120348552016752	0.00155004580931442	0.00788961063614485
ACP7	1.23595367968354	0.211842882349062	2.0562275039612e-07	7.61987616755694e-06
TYMS	1.11798639953727	0.111529209665416	0.000819845710327241	0.00479955679670955
COL4A1	1.23031596289498	0.207271016798626	1.11855207727936e-05	0.000170969191396617

STEAP1	0.865505277424572	-0.144441806840186	6.63930245142435e-05	0.000667950135298571
SH2D1A	0.808689649133279	-0.212340058352308	0.000304303554717444	0.00218006621745418
PTP4A3	1.13737710018948	0.128724822216346	0.0124053093254604	0.0394199557899114
SMC4	1.20722529258336	0.188324579696155	0.000152082619034687	0.00125742788225951
ISCA1	0.804383903932413	-0.217678631313166	3.58740882955476e-05	0.000416846413818422
PTTG3P	1.16472415299343	0.152484280434782	3.72162137279355e-06	7.31682125620594e-05
AQP7	0.839052698836584	-0.175481763001692	0.00295558197875436	0.013067985212501
BCYRN1	1.16528121458979	0.15296244380275	1.92205868516347e-07	7.31253667275701e-06
PCDH18	0.800020418336634	-0.223118028719121	1.05649396511105e-09	1.94357157653108e-07
JSRP1	0.924560568724308	-0.0784367151854485	0.0128194769517751	0.0405359888143606
RPL36A	0.88026524208111	-0.127532005469386	2.16362938450331e-05	0.000286500127495541
MCM4	1.21867259477763	0.197762229335621	1.96927026692708e-07	7.40416871893534e-06
PDZK1	0.943125589307542	-0.0585558246145814	0.00257226497343956	0.0117462206366907
EGR2	0.871698313748794	-0.137311885406315	3.28255935882587e-05	0.000394136672664617
FST	0.868437020170785	-0.141060211565613	1.05690075615084e-06	2.70850537061341e-05
DENND2D	0.845749690278485	-0.167531837530686	0.00130301869131257	0.00689101568680806
PPP1R14A	0.842865140750586	-0.170948309162541	0.00151304863497481	0.00775493882463206
MMP25	0.83480202289959	-0.180560680567377	0.00021876314947939	0.00169693823565059
RGMA	0.859500198166394	-0.151404223599173	2.01397464142332e-05	0.000270155817134675
GINS2	1.09762650776354	0.0931501283570672	0.00300414892837414	0.0132146636465032
SLC30A2	1.13646125816421	0.127919275004583	0.00177302028274014	0.00869793092989949
SPIRE2	1.17947292887175	0.165067668242946	0.000168332593347983	0.00136333520178531
SDHAF3	0.865867838535435	-0.144022993456958	0.000120981443465449	0.00104735363914374
RLN2	0.719020014527041	-0.329866085032331	2.90370966746784e-08	1.89470435606092e-06
PGR	0.870226048926489	-0.139002274730242	6.41015422163506e-06	0.000110666406655553
OCLN	1.1308313793175	0.122953096114023	0.0106296245420811	0.0351884293163622
CA9	1.16580796756958	0.153414381028312	1.96093132366846e-05	0.000265809401268848
TRIM6	0.86704870479177	-0.142660127549005	0.0032617897861805	0.0141070354228512
ADGRA2	0.833334549219306	-0.182320097731852	0.000701536517613176	0.00423635943988918
PIGQ	1.13296904364121	0.124841659202358	0.0044423348330307	0.0179189246084112
ZNF415	0.85431275549425	-0.15745792800515	0.00390315421318554	0.016319113110486
TP63	0.732282679086424	-0.311588665951332	6.94640546203471e-10	1.51940117849743e-07
RPL13A	0.844440939555136	-0.16908048057936	6.22097158148952e-05	0.000638331167654433
EIF1B	0.897662190110056	-0.107961461731822	0.0165719126594552	0.0496578953512818
S100A8	1.03951420833793	0.0387534966572768	0.00355045176158542	0.0151519279402871
RBP5	0.872352743123275	-0.136561414939734	0.00262209172542454	0.0118986087774355
RPL13	0.795068757018474	-0.229326681251884	1.09750810389982e-05	0.000168251911999642
FDCSP	0.945940080907114	-0.0555760513579401	0.0017805767514113	0.00872668967318708
VSNL1	0.85013788719517	-0.162356722424442	0.00886322522921451	0.0305585496356653
SELENOF	0.868485160620088	-0.141004779664464	0.000384349842126745	0.00262919792403036
RTKN	1.18795882562059	0.172236561771302	0.00227010236480496	0.0106018742655381
NPTX1	0.838700462458199	-0.175901653593093	0.00342876579493436	0.0146812739897813
CENPE	1.29331243883497	0.257206709296599	8.43336642845602e-08	4.17694908394009e-06
CRYAB	0.931534024430388	-0.0709225631122527	0.000865424214733153	0.00497522335947597

TMEM74B	1.09322204766199	0.089129342872422	0.00681441872377854	0.0249297378168915
ZCCHC24	0.809810706747052	-0.210954754000682	4.65264599318408e-07	1.39335927388902e-05
RSU1	0.843850171138798	-0.169780322492532	0.00237787243271468	0.0110246812789499
GRHL2	1.39915844970209	0.335870948528103	1.99789259766632e-10	6.43196548161201e-08
PRCP	0.897028484104342	-0.108667662573176	0.00979767521215563	0.0330719692121977
IRS2	0.86682032247288	-0.142923564194588	2.80782288906848e-05	0.000353620921799309
RAB11FIP1	0.92620423233042	-0.0766605153816632	0.00923579991043797	0.031506002297837
GSTM2	0.930838546133847	-0.0716694365787794	0.000307318265309857	0.0021986060897376
SMIM19	0.857524928703424	-0.153705028990351	0.00224391974077804	0.010517225281845
ASPM	1.15886895636471	0.147444491843254	9.23598762980326e-06	0.000145044427686331
ELN	0.89282939025459	-0.113359768704946	0.00332544616776934	0.0142983081887979
NACA	0.860914829831466	-0.149759699503687	0.00419550667210088	0.017188191166583
CPNE1	1.30603803804147	0.266998156036119	7.46550060457343e-08	3.77007780530958e-06
G0S2	0.8541409906565	-0.157659004374606	0.000884737961443957	0.00506218055549755
SVEP1	0.882993110419506	-0.124437880878032	4.90051194586748e-05	0.000526193414399999
AKR7A2	0.837210921742142	-0.177679242936209	0.000922375776287747	0.00520456461724019
KLRB1	0.817819182784047	-0.20111401475535	1.39604314456922e-09	2.17018844349997e-07
PIM2	0.910338470144936	-0.0939388034115004	0.00634602593489628	0.0235676853573545
WDR34	1.22936247950203	0.206495725667665	0.000207804629590754	0.001629226251175
SLC4A8	1.15401702280518	0.143248919108112	3.02307267349536e-05	0.000371143899281198
DNALI1	0.935979235632489	-0.0661619869039408	0.00261198775034452	0.0118749769655998
AKT1	1.21958734220679	0.198512557434933	0.000136846148121341	0.00115936596870563
SAPS2	1.10869635403668	0.103184869287721	0.0105486353894091	0.0350109516948687
RPS4X	0.811601280391425	-0.208746093417842	2.94265867763296e-08	1.89470435606092e-06
PENK	0.752333599048367	-0.284575437644869	0.000311654562494513	0.00222345242577457
STIL	1.2077227537892	0.188736564718881	1.40543788148836e-05	0.000204503122247078
PRDM1	0.875914620156852	-0.132486658370193	0.00470044387506061	0.0187412247360833
CACNA1H	1.09755986827006	0.0930894141559998	4.00632647340344e-05	0.000450580516692164
NANOS3	0.83791551139613	-0.176838005305901	0.00195932123851721	0.00941461166007662
SASH1	1.13897571895679	0.130129366377227	0.000838431069324458	0.0048579959933524
MSRA	0.814279747536657	-0.205451301828217	0.000154921548595513	0.00127476181599919
RPS9	0.827040475302091	-0.189901642831909	0.000366562537954089	0.00252766215930591
CABYR	0.890275008175532	-0.11622486603595	0.00583081950847429	0.0221309630752321
LZTFL1	0.855371947542493	-0.156218878276423	0.00294907985637372	0.0130504384365817
CENPP	0.843382268988752	-0.170334961143275	0.00174201046853262	0.00858879415640068
ARHGEF3	0.880302095928726	-0.127490139592601	0.0100703248084819	0.0337709915940691
AK5	0.862503502674181	-0.147916069013355	0.000344477011839004	0.00239783930808474
AADAT	1.10279660531879	0.0978493219244598	0.0151123000381095	0.0461978976239181
EGFLAM	0.88458771386465	-0.122633602560652	0.00596535685555036	0.0225275316443841
RPL23AP64	0.91710987602721	-0.0865279927180412	0.00514125726169328	0.020106535338485
GSDMB	1.12572907623803	0.118430893518289	9.02625166591635e-06	0.000143500686207207
ZNF689	0.896502415226496	-0.109254291878676	0.00969957191773466	0.0328053151334545
PLAC9	0.848703022831315	-0.164045950293378	5.78555776630614e-08	3.3406706085039e-06
FAM89A	0.881721747836619	-0.125878751392491	0.00700335895580457	0.0254223410721278

MYH11	0.923411876250011	-0.0796799075559471	0.000162507199773149	0.00132456249694635
PPM1B	1.13298035933597	0.124851646799385	0.00562791645209325	0.0216986509316859
C4orf48	1.11674934442856	0.110422094214823	0.0016942774515591	0.00840772943447104
SERPINA3	0.948159573953845	-0.0532324639369751	0.00113424328535468	0.00615696592797422
SARAF	0.822804248412957	-0.195036957856241	0.000257857165176187	0.00191111116233459
HDC	0.935584651035336	-0.0665836498840936	0.0155283749430551	0.0471341539962739
CXCL16	0.880714021078769	-0.127022312917334	0.00671640965514997	0.0246236484937206
BIK	1.19209757094271	0.175714420110948	2.08084208382859e-05	0.000277679211756504
RPS25	0.862711025621444	-0.147675492615625	1.06352788380801e-07	4.93534426080636e-06
PEX11A	1.16415268015896	0.151993509228851	0.00271352740797247	0.0122608593670756
IL23A.3	0.854245003118851	-0.157537237440366	0.00394163051966664	0.0164504651277662
CAVIN2	0.762461767292297	-0.271202913013689	3.32940544037477e-06	6.67306125422975e-05
PLAC9P1	0.875333743580751	-0.133150044111966	1.8683043531688e-07	7.31253667275701e-06
SLC16A9	0.845804842327305	-0.167466628815535	0.000737488348052661	0.00439167916857717
ABCC12	1.12193133616509	0.115051607514021	0.000337160228779754	0.00235966350332135
SERPINF1	0.885397345147677	-0.121718757166481	0.000410145218019341	0.00276888337879112
TPX2	1.25801232535265	0.229532955807873	5.79078421162285e-10	1.38306786042944e-07
CD247	0.91758708697808	-0.0860077858705295	0.00251379056912123	0.0114995872305004
MTHFD1L	1.16293198189643	0.150944386784448	0.00152137547905977	0.00777886361862536
SERPINB5	0.910973239988501	-0.093241756480176	0.000668533404611992	0.00408011323122793
ITGA5	1.17711281504637	0.163064673341926	0.00113433520979374	0.00615696592797422
EEF1B2	0.870522490257309	-0.138661684085456	8.47063120604286e-05	0.000783343291603712
CTSS	0.873187593724153	-0.135604862235781	0.0166798825443106	0.0498440005290819
HSPA12B	0.80025252128792	-0.222827949511798	1.9170975879598e-05	0.000261242584010077
PTH1R	0.844803452290649	-0.168651279520203	0.00102451364266068	0.00567448362725287
PTK6	1.15015287586309	0.139894869073134	0.000739338458423529	0.00439761247036905
GIMAP6	0.885936367855618	-0.121110150532257	0.0049207984604283	0.0194230136932308
MIR4435-2HG.1	1.1343554368182	0.126064592542266	0.00695031501484201	0.0253362797453499
DTX2	1.15739664890508	0.146173214788868	0.00466278763686765	0.0186186194709343
ALG5	0.865001609230003	-0.145023911670482	0.00811896424790386	0.0284806466208225
CTPS1	1.15670878007857	0.145578713917683	0.00303900198446279	0.0133451826274236
EEF1A2	1.04102828398968	0.0402089592826395	0.00704257419208626	0.0255466899038284
STEAP1B	0.890724327877513	-0.115720295705779	0.000100934681193297	0.00089486151949513
CSRP1	0.890802366001211	-0.115632687561577	0.0161948316095211	0.0486979437365108
CLECL1	0.788464846330052	-0.237667456540488	9.00319667665792e-06	0.000143500686207207
ETS1	0.876347189179622	-0.131992931885655	0.000137256005113948	0.00116092887084064
CDCA3	1.15688562697212	0.145731590232878	9.87276316165699e-05	0.000879608688210667
PTBP3	0.872568872812077	-0.136313690700509	0.0138257277195448	0.0431614081717425
NFU1	0.878786751565548	-0.129213014182102	0.015701448628538	0.0474637100267601
CKAP2L	1.3058964156898	0.266889713535503	4.17922390439814e-09	5.00632147245461e-07
ENO1	1.22758636148157	0.2050499337975	6.67526124795148e-05	0.000667964298811124
SRSF5	0.845551668045266	-0.167766003054351	0.00167807324453195	0.00837573186296906
SS18	0.884061916071099	-0.123228177992826	0.0151573018102899	0.0463079843563484
FAM110A	1.26730262889209	0.236890727507653	3.1235149333974e-07	9.99924153738981e-06

INMT	0.791998118330671	-0.233196263015645	9.63560715613018e-07	2.49412122920736e-05
EDN2	1.15499770856938	0.144098360049174	0.00158093880286656	0.0079915758327435
PVRIG	0.912622210014284	-0.0914332737103722	0.0141315939473888	0.0439564253762076
IGF1R	0.92240598677996	-0.080769819610406	0.00227021302948719	0.0106018742655381
PROS1	0.807293125007645	-0.214068448649084	7.81449587538563e-09	7.45416078779841e-07
CYB5D1	0.890013682701052	-0.116518442552721	0.0130560645935204	0.0411577654352654
HACD3	1.28326153275103	0.249404909560718	8.70655960266115e-09	7.60126923954366e-07
GABARAPL1	0.84868639290223	-0.164065545006213	0.00399221708251513	0.0165971833672602
UBD	0.927067134360248	-0.0757292949379723	7.41523010421592e-05	0.000707782272985573
SCARA3	0.845493510499227	-0.167834786013907	7.35175288956486e-05	0.000705193279965523
RPL9	0.85809321352802	-0.153042544919576	3.0146629209924e-07	9.8907826153069e-06
C5AR1	1.15890153390681	0.147472602945913	0.00623388155969753	0.0232760483686677
IGKC	0.950944389500369	-0.0502996939610676	4.10771243346897e-05	0.000458678348072957
LSS	1.18012576966772	0.165621017262026	0.00134328025522619	0.00704606577868644
WWOX	1.19965123893925	0.182030880334352	7.83158566274191e-06	0.000127806079910719
IL20RA	0.86826133160063	-0.141262536362634	0.000342121406440241	0.00238466490470051
CST3	0.892735096824876	-0.113465386206284	0.0048880099117453	0.0193529124176787
NBEA	0.878699297014856	-0.129312536509275	0.0166062685499591	0.0497030152823006
ANLN	1.19019211465722	0.17411473498168	0.000302826212320232	0.00217250392710517
PODN	0.921575903680847	-0.0816701355619637	0.0024393798947665	0.0112204802707349
GAPT	0.841236433014856	-0.172882525327264	0.000275475850890012	0.00202421698706769
DNAJA4	1.13892870342162	0.130088086739054	0.000341437156649341	0.00238312031693869
NAA38	0.837970764641829	-0.176772066170878	0.00160692872783595	0.00807540475812972
NDUFAF6	1.13953115442257	0.130616909762126	0.00261446074222069	0.0118757383449548
MN1	0.892548255972626	-0.113674698473314	0.00803776648340706	0.0282418384420394
COL4A6	0.881698041281828	-0.125905638416146	0.00380410459468692	0.0160089640785735
BEX1	0.949507567568923	-0.0518117787047661	0.000444260111641492	0.0029565682623583
EPB41L5	1.1705440293912	0.1574686231245	0.00324014678359083	0.0140406186359156
NBPF1.2	0.830739454560013	-0.185439065707011	0.000698884477817369	0.00422529805779022
LAMA3	0.845841666174126	-0.167423092714296	3.68167162903139e-07	1.14242714223739e-05
GSK3B	1.46475025302913	0.381684752188358	7.42167842322482e-14	3.82290655580311e-10
JUN	0.904267254693708	-0.100630326558462	0.00658223358443118	0.0242525645160265
WDR19	0.808883956039469	-0.212099813449074	0.000151854063215517	0.00125742788225951
MYOF	0.866535938062264	-0.143251695715663	0.000331930558746185	0.00232939279032915
ALDH1A1	0.856285383151446	-0.15515156687094	4.36785643124119e-07	1.31572096358616e-05
ZNF148	1.20794588244401	0.188921299207789	7.57355289000393e-06	0.000125438491756946
ANXA1	0.905726887564115	-0.0990174669886506	0.000923773122544662	0.00520456461724019
IQGAP1	0.875278094824676	-0.133213620462672	0.0125974772207339	0.0399566534261087
PLGRKT	0.862212473234307	-0.148253550020896	3.82257089592284e-05	0.000435474057279173
PYCR1	1.24977448725576	0.222963124842943	4.22420010424274e-06	7.99957894740969e-05
TNFRSF14	0.822317011206845	-0.195629299870557	7.43506870771182e-05	0.000707911994702839
DHCR7	1.14156995128673	0.132404465256227	3.57335180280901e-05	0.00041643292163505
FAM162B	0.86333424857447	-0.146953352832357	0.00923962169998874	0.031506002297837
CLCA2	1.05786590455258	0.0562535811241477	0.00461588477419752	0.0184599553353194

MAOA	0.905889532664231	-0.0988379089997334	1.36782489556756e-05	0.000200731226127308
CYP4Z2P	1.07858015770882	0.0756455074128372	0.00999212666093095	0.0335524409585758
IGSF5	1.13930764482936	0.130420748810327	0.00556032245334863	0.0214701806275853
FBLN2	0.918138737698499	-0.0854067693901722	0.00231383097362918	0.010776259805754
CAST	0.862011103667635	-0.148487127116447	0.00150327079960201	0.00771249789716131
CYP4F12	0.88142867989131	-0.126211188084674	0.0150565473190873	0.0461041817572321
FAR2	1.08940289386672	0.0856297423497368	0.0156562552804074	0.0473548860536573
SLC22A23	1.12542559428477	0.118161270147603	0.00443045362153792	0.0179189246084112
GALNT16	0.911245958033315	-0.0929424312978423	0.000811438681262821	0.00476050187606468
ITPKA	1.11951531219612	0.112895834672934	0.00836927769463743	0.0291481740399441
PRKCA	0.796978522001696	-0.226927549110002	5.54425044267349e-05	0.000580455976223804
TEDC2	1.29370835928404	0.257512791452914	3.97096521020652e-07	1.21032199986827e-05
UCP2	1.10242193679535	0.0975095202042226	0.00243897947900514	0.0112204802707349
KRT18	1.1250352131561	0.117814335749729	9.70036813096725e-05	0.000865972205244581
BCL11A	0.901658809037828	-0.103519090968271	6.93160309322934e-05	0.000680089286347131
HSD17B11	0.851798480705723	-0.160405305122552	1.14820042253812e-05	0.000173442239779879
ABCC5	1.17008424686596	0.157075752085707	6.29666607007188e-06	0.000110320159615443
RBBP8	0.814937246085656	-0.20464416737373	9.38338299840653e-09	8.05563430413201e-07
CLN3	1.17106627731617	0.157914681917572	0.000548307781641353	0.00346119287161104
ARSD	0.90656809198724	-0.0980891363437743	0.0147078207668271	0.0453443538878462
ZNF521	0.763384290418324	-0.269993717323506	1.68192592961048e-06	3.86767877831411e-05
CERS4	1.16046329505391	0.148819317670254	0.000362712556138901	0.00250882404760259
ATP6AP1	1.26129983931633	0.232142807715719	2.87826910656857e-05	0.000359853499221717
EPPK1	1.09148229426169	0.0875366754028063	0.00520523685559256	0.0203122538205737
CBX2	1.117361784358	0.110970356955104	1.1055896074122e-06	2.7779961306245e-05
SLC38A10	1.3123608044799	0.271827656129945	6.54579055173223e-08	3.47601723010028e-06
RPL3	0.869576812998398	-0.13974860751591	1.62279419096827e-05	0.000228422417471678
TF	0.902888629849028	-0.102156066671876	5.26896809627948e-05	0.000558445569216782
SPRY2	0.734267064255725	-0.308882468784323	1.03159698670357e-10	4.08750467577699e-08
ARID4B	1.15672256202502	0.145590628640371	0.000682642484598526	0.00415146568850886
ARPP19	0.875819731636461	-0.132594995025676	0.00763633120644894	0.0270931784842348
KCNH1	0.848082561345164	-0.164777287852574	0.000564065043605723	0.0035303755037826
HOXA5	0.870941160343657	-0.138180858551981	1.42709874917839e-05	0.000206488361152187
TTC23	0.815905588951566	-0.203456630526027	0.000328354237708035	0.00232009969606871
TMEM176B	0.86376267335184	-0.146457231531092	0.00966182832326598	0.0327522907132124
MTHFD2	1.16454991473852	0.152334673089177	0.000865085844531984	0.00497522335947597
CNST	1.22571647221857	0.203525548304274	3.64859593504468e-06	7.22842986977506e-05
GNA13	0.875375236370784	-0.133102642984176	0.00714601070339789	0.0258014606677908
LAPTM4B	1.11189887534177	0.106069252252296	4.61624871213259e-05	0.000503777481275317
ZNHIT3	0.889958801445281	-0.116580107838311	0.00709120290556143	0.0256688588661609
ZP2	1.16421661259947	0.152048425294551	0.00102748904289495	0.00567875113728741
CEL	1.07761350647882	0.0747488798873802	0.00967117728107086	0.0327522907132124
RBP1	0.931192868682918	-0.0712888602356216	0.00085752758158193	0.00494638809936005
INPP1	0.771275153521016	-0.259710090328702	1.8234668065357e-06	4.17452334242907e-05

FCN1	0.91440370655623	-0.0894831129705675	0.00589611626054805	0.0222987480602665
POC1A	1.36479425206775	0.311003686189957	7.0959438858993e-10	1.51940117849743e-07
DBN1	1.3040112896988	0.265445121211465	1.56197572996026e-08	1.13320239225708e-06
SYTL2	1.07204704384263	0.0695699458672859	0.0108579858529003	0.0357376901778206
COL4A2	1.17018453056808	0.157161454807679	0.00197720936535081	0.00948287285002049
TIPARP	0.865708710921831	-0.144206788524062	0.00714787116539262	0.0258014606677908
RGS22	0.875614107220811	-0.132829801973027	0.000291078058853276	0.00211410652967296
OSBPL10	1.18262568292052	0.167737121503903	0.000284530195838946	0.00207888657981051
AURKB	1.19187711959285	0.175529475739373	4.85441428092721e-07	1.43286649214431e-05
CDC42SE2	0.829476669287356	-0.186960295983045	0.000991714321371693	0.00552728670535227
C2CD2	1.16565237832108	0.153280911678081	0.000514345743054711	0.00329526731651096
FABP6	1.13649613367382	0.127949962345868	0.00694155534643358	0.0253339514481279
RPS15A	0.913510358214936	-0.0904605641862861	0.00939101437541161	0.0319716556825811
MTFR1	1.15152741138133	0.141089244929119	0.00112912321849611	0.00614161953376291
DAPL1	0.881707184492455	-0.125895268467716	0.00393642014840896	0.0164448501090467
SRI	0.88590458044596	-0.121146031190385	0.00951511598012694	0.0323300543625553
MCM2	1.22796644653818	0.205359505685678	1.08958868835679e-06	2.77393412612547e-05
DHDH	1.14442344899992	0.134900972197038	0.011811181916453	0.0379930390529917
USP8	0.878369544774981	-0.129687880076816	0.0142489549854054	0.0442680139504363
FOXO1	0.873048490990418	-0.135764179449812	0.0156493949867011	0.0473548860536573
SCGB2A1	1.04304255308513	0.0421419739257322	0.00155414549831012	0.00790266876781387
ECM2	0.845402285728906	-0.16794268712835	5.53149794190251e-07	1.58705563424761e-05
WNT4	0.884680116272758	-0.122529149852456	0.0163262179586445	0.0489786538759335
ECT2	1.14612077941196	0.136383004896218	0.00115316345508176	0.00624599890339238
LYPD6	0.875231814609516	-0.133266496730234	6.36491836003807e-06	0.000110666406655553
EVPL	1.19992995677865	0.182263185739277	9.43761833929335e-05	0.000847396201902037
PPP2CB	0.855947843084535	-0.155545835672772	0.00119719607628839	0.00643040353384933
NT5DC2	1.11338346148707	0.107403542594682	0.0101074759043527	0.0338735252981919
RPL13P5	0.788464967460715	-0.237667302912011	1.35951798882461e-05	0.000200082204583874
OGFRL1	0.85798898619357	-0.153164016180795	0.00602760965592192	0.0227459467675119
BRI3BP	1.14216813965773	0.132928333021146	0.0142793511229734	0.0443089985749614
CLPSL2	0.941495569123783	-0.0602856370842322	0.0108697598891778	0.0357535971833684
SNAR-A3	1.06540154932334	0.0633517697172202	0.00021907666991024	0.00169693823565059
SLC16A5	0.905273978384454	-0.0995176425519196	0.0117297457669033	0.0378333878806005
TENT5C	0.886486899493147	-0.120488931362576	8.19315225742385e-05	0.000761785690938452
TPRG1	0.930335279361641	-0.0722102423622879	0.0117414852082565	0.0378475533840609
DNAH2	0.845914516666835	-0.167336968610253	0.00382987657754665	0.0160880735127166
HOXC4	1.10090720216256	0.0961345691407326	0.00924200533333949	0.031506002297837
ABCB9	1.196102431284	0.17906829674741	6.9179038583763e-07	1.90556806280729e-05
EFEMP1	0.935529122202328	-0.0666430036591747	0.00292649943264413	0.012995171187543
PTHLH	0.847102829405872	-0.165933187441755	6.27560523482336e-08	3.4294988000839e-06
SLC25A1	1.30214276982353	0.26401119201713	1.4563064662749e-07	6.26781365989393e-06
QDPR	0.903693701418481	-0.101264801868926	0.000268566612262339	0.00197909387662848
CENPBD2P	0.917660498377772	-0.0859277842398867	0.00610013941407472	0.0229523872329429

EPHX1	1.19668839704251	0.17955807275565	1.52649498869885e-05	0.000217810960852848
ARHGEF10.1	0.876958123410582	-0.131296037566909	0.0159502399499148	0.0480465999894803
GJA1	0.931502692976466	-0.0709561979339743	0.000722460838163893	0.00432217860323137
HTR2B	0.873603367084945	-0.135128819735022	0.0107595414130616	0.0355956312258705
LINC01116	0.929251774994995	-0.0733755596693974	0.00621295728151664	0.0232410624234511
COMT	1.17633901400526	0.162407085147903	0.000625882669001676	0.00385175821747626
GGT1.1	1.13860357903008	0.129802580890245	0.000824606349796642	0.0048158132741525
SELL	0.901608599490959	-0.103574778268607	0.00633977926376978	0.0235614740170838
CP	1.12005430111025	0.113377167265883	0.00169697417833857	0.00841300673014625
ASPSCR1	1.20332449661436	0.185088140115942	0.000830595040968391	0.00482888832508824
PDGFRA	0.802998793828377	-0.219402067118226	3.99828821053195e-09	4.9036148982024e-07
STXBP2	1.15027745449613	0.140003178054739	0.0153861992225096	0.0468129428205238
COMTD1	1.22972344758672	0.206789304743775	4.06206536473928e-06	7.80735025887016e-05
SFRP1	0.911722268341492	-0.0924198656138935	1.76735703681618e-07	7.11223132550011e-06
ADIPOR2	1.10433049098267	0.0992392608304627	0.0144442466538243	0.0447848572641137
ZNF22	0.799882622590771	-0.223290283840436	1.37705989336157e-05	0.000200941515884006
GOT2	1.15170358332116	0.141242223019966	0.00406203411952275	0.0168330955347238
PHACTR2	1.16312838698721	0.151113260381951	0.00389031173556784	0.0162918664633414
PI16	0.881102664490243	-0.126581128064266	0.000221874962467182	0.00171603293043311
GALE	1.11361650745788	0.107612833995584	0.0146252306569971	0.0451846541346966
TMPRSS3	0.936697712395889	-0.065394660965093	0.00680234681698969	0.0249032611615593
LMTK3	1.10999513053974	0.104355628413485	3.80686594610981e-05	0.00043479304852354
ABI3BP	0.813048828563544	-0.206964111504084	9.90723634809125e-08	4.72520133602019e-06
SIDT1	1.1482411636527	0.138231348703366	0.00444738960451282	0.0179252768801608
OIP5	1.21537385493603	0.19505172899855	8.41045313300904e-06	0.000135613259606554
RPL10	0.875373968014799	-0.133104091913563	0.00139230751692642	0.00725888261102021
ACSL4	0.873007026716452	-0.135811674244284	0.00444088440975632	0.0179189246084112
SLC66A3	0.896641197266469	-0.109099499993315	0.00730327128985909	0.0261971799540837
KANK1	0.893158018695089	-0.112991761133828	0.0127658407193387	0.0404160083253312
CARS1	1.27685529878904	0.244400257230615	7.45757149191737e-07	1.99036014273919e-05
C20orf24	1.13489323168971	0.126538577512752	0.00569577290191883	0.0218458125225494
ACSL5	0.878200317545924	-0.129880559267335	0.000803761546661798	0.00472622799869283
ANKRD65.1	0.897532367427265	-0.108106095284183	0.005729255552849	0.0218764976669572
CATSPERB	1.39234985115369	0.33099286018596	1.43631493920612e-10	5.28461303703625e-08
EVI2B	0.911457741981859	-0.092710046901524	0.00660905796037784	0.0243109137763345
NMU	1.10451061402798	0.0994023536353762	0.0135151009656173	0.0423199301361061
ZFP36L2	0.869571801290371	-0.139754370919895	0.000184644885186373	0.00148147321432244
TUBB2B	0.887797651141959	-0.119011432291988	5.5459136898577e-07	1.58705563424761e-05
PLPP3	0.876020441302721	-0.132365853497541	7.96417608658497e-05	0.000747239909326033
RARA	1.09714886412262	0.0927148732010442	0.00445671122389886	0.0179488033731845
SEMA6A	0.911163145625453	-0.0930333136444599	0.00331866207788117	0.0142930003036504
OXTR	0.873710324890515	-0.135006394315711	0.000684718221285803	0.00415917872387166
INAVA	1.09456574804688	0.090357707484826	1.78685800439842e-05	0.000246099079696692
CCT2	1.16894466782646	0.156101348458198	0.0010759589752823	0.00590230530530257

KLHL8	1.26042982900126	0.231452796918357	1.07287888811779e-05	0.00016496713888641
ACE2	1.10073725679285	0.0959801887462374	0.0155061325940968	0.0470943920944533
EVA1B	0.869603428706111	-0.139718000328597	0.000167608175266317	0.00135960584377448
CR593862	0.895406480868573	-0.110477495242371	0.000464621578634843	0.00305263488717867
SNHG29	0.838297786451293	-0.176381887862755	0.000162516695412559	0.00132456249694635
CCL19	0.901474726556319	-0.103723271611041	3.18878389625396e-08	1.97896696983183e-06
STAMBPL1	0.788869479039504	-0.237154397626065	3.00913227783366e-06	6.22491580848241e-05
SLC4A11	1.13993650989342	0.130972567779542	0.000261414882137081	0.00193469548547141
LRRC26	1.06653295509969	0.0644131586859873	0.00154095886298432	0.00785889020122004
TRNP1	0.794130702117039	-0.230507219044025	4.11394674450992e-05	0.000458678348072957
LRMP	0.798232440177906	-0.225355445527222	6.54233341956419e-05	0.000659482572293055
TPD52L1	1.09074471606688	0.0868606886980271	0.000994709241595904	0.00553320443138283
PCDH19	0.900290221361407	-0.105038099460173	0.0114348964379169	0.0372085606770121
ENC1	1.29283055094214	0.256834040112947	4.87451773824727e-08	2.95051958867963e-06
GSTM3	0.879136713212658	-0.128814860725771	4.597475198721e-06	8.45771241021852e-05
APOD	0.954472700940509	-0.0465962366019607	0.0028838682116557	0.0128604922803744
USP13	1.17469629454431	0.161009641458045	0.00105484300942961	0.00581744790318191
FOSB	0.939672466530961	-0.0622239042980406	0.00110949266473849	0.00604761557255871
MYLK	0.87164807835999	-0.137369516395825	7.2113137989247e-05	0.000697273705984921
CLEC3A	1.06060737687286	0.0588417410737192	5.23116065928381e-07	1.51380385145904e-05
CFAP69	0.845683586245273	-0.167610000866081	0.000256400910284894	0.00190580243705266
HLA-DQB1	0.933230966308194	-0.0691025564576345	0.00407995142649191	0.0168595673187426
SH3BGRL2	0.846304541245339	-0.166876006321018	9.28266066072183e-05	0.000839355103309347
PDGFRL	0.860873801732221	-0.149807357036674	6.39161104267239e-08	3.4294988000839e-06
TBX3	0.916179734327869	-0.0875427170408089	0.0142353843466196	0.0442525436146273
SULF2	0.856379023984161	-0.155042215812533	6.32620955258126e-06	0.000110462052221512
CHEK1	1.15381690889474	0.143075497694908	0.00304274223468371	0.0133502259376966
ASCL2	1.07191359333436	0.0694454561671236	0.011943861207944	0.0382842744754944
HSPB1	1.09137073842964	0.0874344643693766	0.014725936689627	0.0453443538878462
SNORD13	0.880476654905418	-0.127291864849473	5.77791052827182e-06	0.000102627645279752
NOSTRIN	0.925432416448294	-0.0774941734617575	0.00428498958021442	0.0174896840948372
FARP1	0.90493119885858	-0.0998963615328187	0.0129171689858287	0.0407948114322523
FGB	1.08003871779504	0.0769968903037629	0.0104456471800486	0.0347805614896124
PSMD12	1.16817579942084	0.155443386282837	0.0022258822176921	0.0104612402402665
GYPC	0.85651008860147	-0.154889182418132	1.65148711414377e-05	0.000231793191415656
BHLHE22	0.750969587510252	-0.286390124029663	6.69809283504533e-08	3.52059961156311e-06
AKR1C3	0.903414194515332	-0.101574143559045	0.000313968332784087	0.00223377193670004
NKD2	0.923258992860709	-0.0798454848574661	0.00987811365542891	0.0332780663434364
STAT4	0.852701474606833	-0.159345763897253	0.000112026278115595	0.000981373058798354
HILPDA	1.27910435598401	0.246160111124416	1.85353419276629e-06	4.20597120129478e-05
PAMR1	0.816767942564556	-0.202400260496703	2.63083629505793e-06	5.59976766770388e-05
ILVBL	1.17895889069997	0.164631753008377	0.00207733109624782	0.00990771525627085
DYNLT3	0.871748069265986	-0.137254808208312	0.00505435814553096	0.0199044333391666
ANXA8L1	0.839924367922447	-0.174443429386022	0.00633779354267787	0.0235614740170838

SLITRK4	0.846731952761203	-0.166371101049978	0.00146191325769097	0.00753031519036618
ANXA2R	0.883973302992349	-0.123328417035429	0.0065966548115794	0.0242883266150432
ALDOC	1.1253292136538	0.118075627206235	0.000174002119108154	0.00140263680051033
CNBP	0.899867867378215	-0.105507340460265	0.0118421753400301	0.038029329910533
KLHL13	0.76942920538973	-0.262106330728609	1.45303414466161e-05	0.000209652069444033
RPL22	0.866338452470381	-0.14347962405694	0.000230065838767186	0.00176548998064058
IGFBP6	0.799340317621376	-0.223968494459539	1.16126592051208e-08	9.34637618212147e-07
GRB2	1.18379286765194	0.168723578295989	0.0014384535284506	0.00745419932097489
ANKRA2	0.850561921838669	-0.161858063401805	0.00306465722286422	0.0134160529582974
HMGA1	1.14204554716698	0.132820994122359	0.000482690537472197	0.00314328566184486
RUNX1	0.887227353669564	-0.11965401196323	0.00454334574854966	0.0182406655890719
CEACAM6	1.0537204265158	0.0523271649491631	3.69711024977774e-05	0.00042603612744083
MMP3	0.904383066321211	-0.10050226241437	0.00402459176736006	0.0167048124042479
MED24	1.12810675968835	0.120540793711083	0.00041761052943471	0.00280092687124764
CRB3	1.13962590352742	0.130700053751324	0.00829966362752774	0.0289448661783313
PFKFB4	1.18064470144368	0.166060646434506	0.00127774385091533	0.00679000003497004
ZNF285	0.854719160820044	-0.156982330879578	0.00460441792674841	0.0184416478780899
C1S	0.888727511786074	-0.117964601379108	3.93505571399127e-06	7.64885735198831e-05
RNF213	1.16183705104915	0.150002417141252	0.000712639417450939	0.0042883243449647
OLR1	1.11557687473264	0.109371647560866	0.000836724491025077	0.0048579959933524
NRIP1	0.883338338329419	-0.124046982652726	0.0017424363994251	0.00858879415640068
IGFBP4	0.870926483340047	-0.138197710582804	1.33371456612491e-05	0.000197412750865213
CPED1	0.767026353822053	-0.265234118593616	8.53748615305742e-09	7.60126923954366e-07
GPRC5C	1.11076307670302	0.10504723562348	0.00083752061815131	0.0048579959933524
CEP55	1.17617330432293	0.162266206240435	2.83433952249541e-05	0.000355223427746323
TRAF3IP2	1.2654499429605	0.235427745072946	9.91149560111767e-06	0.0001547094358829
KCTD5.1	1.34847529935189	0.298974546301145	2.47097183505704e-07	8.6584870220264e-06
IGSF21	1.09722505311554	0.0927843135023112	0.000389632542602019	0.00265475823669709
SLC16A10	1.23338515974416	0.209762551513381	9.44293185579051e-05	0.000847396201902037
SELENBP1	1.08985891531642	0.0860482523740317	0.0140837573528797	0.0438340991689928
SHROOM2	1.27565719684599	0.243461494319102	2.83379253321565e-06	5.9579042198342e-05
HIGD1A	0.894486664934965	-0.111505283986895	0.000960499880210544	0.00537775530757012
CXCL17	1.14162489855448	0.132452597169073	0.000183714890326427	0.00147631107655449
S100A7A	1.07085998029065	0.0684620455617371	0.00720606995805702	0.0259439247918666
TMEM35B	0.820749460648944	-0.197537379744794	1.00234004732339e-05	0.000155983491956579
CIRBP	0.86167137180441	-0.148881320214606	0.000425494606564078	0.00284112608563404
TPI1P2	1.13950121385912	0.130590634958955	0.013353052221795	0.0418634035267597
EXOSC4	1.21707768952195	0.196452648879942	0.000170253750320826	0.00137520811571761
HMGB3	1.18751990295016	0.171867017165291	5.70109817608905e-08	3.33708598920849e-06
LMNB2	1.15090574358989	0.140549235500226	0.00588024421666153	0.0222714249706055
TEK	0.847162312838685	-0.165862970058001	0.00407496627380305	0.0168595592581201
MCM10	1.16851049845778	0.155729859515919	1.76754256344699e-05	0.000244091467676017
CPNE3	1.17076635427153	0.157658538039282	0.00440488021920505	0.0178517214863298
GPR65	0.860387099521548	-0.150372875283547	0.00687259993772948	0.0251247425686619

CCL5	0.935605053723315	-0.0665618427012859	0.00249646951964863	0.0114406712595286
NFS1	0.911724142654805	-0.0924178098218486	0.00713310184774203	0.0257842860475223
PCDHB2	1.076039796546	0.0732874466931877	0.00744318763614841	0.0265511492477843
SLC1A5	1.23201409077159	0.208650302360527	5.25581647566974e-06	9.46598275041079e-05
NKX2-2	1.17175291896605	0.158500848934325	1.27647742068122e-06	3.14599770044448e-05
GSPT2	0.800262617216783	-0.222815333662538	1.53917533625279e-07	6.49860012871979e-06
NCEH1	1.16338864984205	0.151336996423253	0.0059149985986842	0.0223537474554822
WDR86	0.80364743344804	-0.218594621591149	2.9373279006962e-05	0.000364582554614124
EXPH5	1.1850561282126	0.16979013904563	0.000881340413764282	0.00505543927761672
TGFBI	1.11304905285979	0.107103143972546	0.00603808290366007	0.0227687884602877
