# Supplementary material for: AdhesionScore: A Prognostic Predictor of Breast Cancer Patients Based on a Cell Adhesion-Associated Gene Signature
Source: Cancers (Basel). 2025 Nov 21;17(23):3731. doi: 10.3390/cancers17233731 (PMC12691146; doi:10.3390/cancers17233731)
Supplement: Supplementary file 1 [file cancers-17-03731-s001.zip › SuppTable3.pdf]

ID	Description	GeneRatio	BgRatio	pvalue
GO:0022626	cytosolic ribosome	46/1610	117/17367	2.02026264565713e-18
GO:0022625	cytosolic large ribosomal subunit	27/1610	57/17367	9.40120086924409e-14
GO:0044391	ribosomal subunit	48/1610	187/17367	4.31188053283676e-11
GO:0030055	cell-substrate junction	83/1610	427/17367	5.01383479449241e-11
GO:0005925	focal adhesion	80/1610	417/17367	2.18210096307623e-10
GO:0005840	ribosome	53/1610	233/17367	5.42416530098864e-10
GO:0062023	collagen-containing extracellular matrix	78/1610	420/17367	1.753411586027e-09
GO:0022627	cytosolic small ribosomal subunit	17/1610	43/17367	1.0257878374492e-07
GO:0015934	large ribosomal subunit	29/1610	115/17367	4.42693081473133e-07
GO:0045178	basal part of cell	54/1610	303/17367	2.06791176761142e-06
GO:0009925	basal plasma membrane	51/1610	284/17367	3.13994472638204e-06
GO:0005788	endoplasmic reticulum lumen	53/1610	309/17367	8.13344021518675e-06
GO:0019814	immunoglobulin complex	7/1610	11/17367	1.37098401948779e-05
GO:0031091	platelet alpha granule	22/1610	89/17367	1.48805922044253e-05
GO:0045121	membrane raft	49/1610	284/17367	1.49812905100952e-05
GO:0016323	basolateral plasma membrane	45/1610	253/17367	1.50314021876736e-05
GO:0034774	secretory granule lumen	53/1610	316/17367	1.56190791629066e-05
GO:0098857	membrane microdomain	49/1610	285/17367	1.64982493930813e-05
GO:0031093	platelet alpha granule lumen	18/1610	65/17367	1.66850838047611e-05
GO:0005604	basement membrane	22/1610	90/17367	1.79914963489123e-05

p.adjust	qvalue	geneID	Count
1.28488704263793e-15	1.15686618867103e-15	6229/25873/6130/9349/6218/6234/6	46
2.98958187641962e-11	2.69171224887831e-11	25873/6130/9349/6125/6168/6154/6	27
7.97199732324294e-09	7.17770033737861e-09	6229/25873/6130/9349/6218/6234/6	48
7.97199732324294e-09	7.17770033737861e-09	10092/682/5880/2017/8500/6130/93	83
2.77563242503296e-08	2.49907983981783e-08	10092/682/5880/2017/8500/6130/93	80
5.74961521904796e-08	5.17674723462776e-08	6229/25873/6130/9349/6218/6234/6	53
1.59309966959025e-07	1.43436977864464e-07	5315/7869/3914/5265/5054/1308/70	78
8.15501330772113e-06	7.34248136279427e-06	6229/6218/6234/6232/6194/6205/61	17
3.12836444241014e-05	2.81666709147818e-05	25873/6130/9349/6125/6168/6154/6	29
0.000131519188420086	0.000118415158061117	682/9123/366/8140/1317/9497/3006	54
0.000181545895088998	0.000163457409679601	682/9123/366/8140/1317/9497/3006	51
0.000431072331404898	0.000388122059391368	2621/5959/80020/5265/51237/1308/	53
0.000558511226306741	0.000502863467578397	5284/3500/974/3512/973/3543/3514	7
0.000558511226306741	0.000502863467578397	2621/5265/5054/7043/22915/1675/7	22
0.000558511226306741	0.000502863467578397	9846/107/25999/83483/857/23180/2	49
0.000558511226306741	0.000502863467578397	682/9123/366/8140/1317/9497/3006	45
0.000558511226306741	0.000502863467578397	10092/5709/2717/2621/6282/5315/2	53
0.000558511226306741	0.000502863467578397	9846/107/25999/83483/857/23180/2	49
0.000558511226306741	0.000502863467578397	2621/5265/5054/7043/22915/1675/7	18
0.000572129583895412	0.000515124948095174	3914/1308/3898/3908/3912/1295/64	22
