# Supplementary material for: AdhesionScore: A Prognostic Predictor of Breast Cancer Patients Based on a Cell Adhesion-Associated Gene Signature
Source: Cancers (Basel). 2025 Nov 21;17(23):3731. doi: 10.3390/cancers17233731 (PMC12691146; doi:10.3390/cancers17233731)
Supplement: Supplementary file 1 [file cancers-17-03731-s001.zip › SuppTable4.pdf]

	coef	exp(coef)	se(coef)	z	Pr(>|z|)
BSG	-0.188131097080533	0.828506085383835	0.0960671395609357	-1.95832933030343	0.0501913812928259
PKM	0.260518712902266	1.29760299554323	0.110473568571868	2.35819948853003	0.0183638214936687
RPL23	-0.149292612028245	0.861317046293726	0.0776794110388749	-1.92190710552029	0.0546174475926134
SLC31A1	-0.303473824289279	0.73824921307673	0.102254458022256	-2.96782976663206	0.00299910380732185
SEMA3B	-0.241601462062843	0.78536911457588	0.0895495571938374	-2.69796378266703	0.0069765030260288
SLC4A7	-0.194315682207079	0.823397931160081	0.0960914326653512	-2.0221957027512	0.0431561380566436
SPRY4	-0.341294930925374	0.71084922586704	0.117598034020005	-2.90221629782786	0.00370532606377175
SERPINE1	0.245484923776989	1.27824101239894	0.0900650689971504	2.72563965708788	0.00641770198963535
COL17A1	-0.139588229506638	0.869716285180412	0.0742897595686011	-1.8789699995965	0.0602485896983761
TGFB3	-0.202698435982064	0.816524438692101	0.0929311181159274	-2.18116859122697	0.0291709490315187
LAD1	0.122687255945963	1.13053079886852	0.042903868582535	2.85958492787073	0.00424195817534049
ANXA8	-0.172673606057358	0.841412201876371	0.0717345104599995	-2.40712043547916	0.0160788664359705
CD59	-0.163719868924793	0.848979814200324	0.0900613433475145	-1.81787060729325	0.069083911690465
FGL2	-0.441909935103346	0.642807527241992	0.135166763349447	-3.2693683280769	0.00107787891019307
C1QC	0.458135501151412	1.58112323256546	0.116782137920359	3.92299292776986	8.74557194781327e-05
SORBS1	-0.227771146202032	0.796306476745692	0.0968468076064261	-2.35187046255223	0.0186792786877553
CEACAM5	0.181505817453751	1.19902151194162	0.0516562086447545	3.5137270468684	0.000441866607896974
A2M	0.302679740430499	1.35348092980428	0.0990053876184103	3.05720474119143	0.00223411619816936
SLC3A2	0.301943994313761	1.35248547771101	0.11028940089388	2.73774262863474	0.00618624683884996
ATP2C2	-0.175828360764771	0.838761935439945	0.0788032848777864	-2.23123136348259	0.0256658074687035
EZR	0.346692687725444	1.41438200162587	0.0891856400238488	3.88731512867695	0.000101359130275171
LEPR	0.244688606302415	1.27722353191656	0.104339571483818	2.34511799140716	0.0190210591033558
AQP5	0.147557064828628	1.15899942104605	0.075753281758228	1.94786366219178	0.0514312788076337
P4HB	-0.16268094438266	0.849862298502995	0.110693845313564	-1.46964760255488	0.141657220664134
SFRP2	0.0991363180917376	1.10421681402868	0.0635780213107809	1.55928599298712	0.118928705454914
RPS13	0.273254229717443	1.31423431977682	0.12466220301912	2.19195733028665	0.0283825878132727
SLC6A9	0.113015899051045	1.11964973417535	0.0702338975517469	1.60913608657098	0.107586588032088
SERPINA5	-0.069800417033453	0.93257992840194	0.0304780907776684	-2.29018338263486	0.0220106883138344
ENPP1	0.245598642263801	1.278386380298	0.0986754912321411	2.48895282097976	0.0128119966225393
ANGPT2	0.331436178511402	1.3929672421984	0.111426768020648	2.97447538323993	0.00293490003296732
HSPA1B	0.190612439184458	1.20999041632468	0.0774885342930178	2.45987927018557	0.0138983760307442
SORBS3	-0.162567867879827	0.849958403393099	0.109774344058245	-1.48092770924298	0.138625838258468
CD300LG	0.205588852556635	1.22824810909832	0.120305689070453	1.70888720346581	0.0874718453202907
KIF23	-0.30827054115432	0.734716520063332	0.113792424026325	-2.70906032446416	0.00674740703454045
REXO2	-0.277794517271569	0.757452448920277	0.112891894507974	-2.46071268873911	0.0138661357501296
P3H2	-0.243613726660871	0.783790333105998	0.102068966783581	-2.3867560761873	0.0169977649614111
PGM5	0.157447898156125	1.1705197701546	0.0926293082282748	1.69976329487544	0.0891754582731819
CD44	-0.132677146706731	0.875747784528535	0.0675459477155809	-1.96425027990429	0.049501074534335
FHL1	-0.325848906186059	0.72191425596999	0.12701020291161	-2.56553330926357	0.0103017329709155
CNN3	0.18928587317509	1.20838634835298	0.0866500895818883	2.18448560282452	0.0289265840726559
ADAM17	0.468800627478193	1.59807635447841	0.148572512594716	3.15536581626651	0.00160296981599214
ATP6V0A4	0.0937522025077976	1.09828755929208	0.0560060102841633	1.67396681234956	0.0941371219733289
FAM107A	0.176255420141087	1.19274267024816	0.0857494575321228	2.05546979787085	0.0398336499140709
RPL13A	0.365305122199908	1.44095360801481	0.109962738843093	3.32208097072923	0.00089348761544595
PRCP	0.178901026979759	1.19590237624027	0.0802818706141696	2.22841129150499	0.0258531008131971
ITGA5	-0.360069499039667	0.69762783992126	0.159604389070917	-2.25601251403981	0.0240698474266639
CTSS	-0.270249451551476	0.763189091886706	0.166857908740031	-1.61963825144501	0.105310007665904
HACD3	0.25979390942472	1.29666282913869	0.0893570556765511	2.90736872939397	0.00364483285348444
EPB41L5	0.440348277729102	1.55324808603595	0.121326562533854	3.62944658228681	0.000284029497482191
ANXA1	0.16521426085845	1.17964584356701	0.115740574406364	1.42745326525151	0.153449286921915
CLCA2	0.0919911438709314	1.09635511257544	0.0428804113199947	2.14529527677448	0.0319292459065865

FBLN2	-0.16972316362929	0.84389840603384	0.0782807318011347	-2.16813460636082	0.0301484464992497
RPL3	-0.196216867882292	0.821833985954544	0.104000583627319	-1.88669006498488	0.0592020247366373
GNA13	-0.496089090489365	0.608907390798917	0.140842869357806	-3.5222875872336	0.000427839745459526
ZP2	0.237008716938899	1.26745216603022	0.0993108743223873	2.38653338374115	0.0170080631063686
COL4A2	0.235461684481872	1.26549289231242	0.134928901564164	1.74507968087105	0.0809709999630069
SLC16A5	-0.136706292220454	0.872226368184672	0.0721876792709326	-1.8937621156565	0.058256592286248
EFEMP1	-0.0720734543045203	0.930462546821714	0.0503453773926034	-1.43158037613815	0.152263949075258
TEK	-0.248093460949609	0.780287013504795	0.151759680225543	-1.63477849044553	0.102095491421225
SLC1A5	0.162901401862418	1.17692064178415	0.0907539732874393	1.79497818069597	0.0726571763834832
TGFBI	0.450021919366156	1.56834656227597	0.112849939226422	3.987790533615	6.66915067051969e-05
