# Supplementary material for: AdhesionScore: A Prognostic Predictor of Breast Cancer Patients Based on a Cell Adhesion-Associated Gene Signature
Source: Cancers (Basel). 2025 Nov 21;17(23):3731. doi: 10.3390/cancers17233731 (PMC12691146; doi:10.3390/cancers17233731)
Supplement: Supplementary file 1 [file cancers-17-03731-s001.zip › SuppTable5.pdf]

Gene	Coef
CTTN	0.0073724671636502
PKM	0.00703576910894851
SEMA3B	-0.121738044397175
LAMB3	-0.014836943931847
SERPINA1	-0.00782429976026681
SERPINE1	0.0393586158578353
COL17A1	-0.0697185587247556
TGFB3	-0.0135046230339521
C1QC	0.0966988366244997
SLC39A8	-0.018500706747714
CEACAM5	0.0973209701143567
RPL6	0.026525714489153
EZR	0.183989041807977
GREM1	0.0446969419762387
AQP5	0.063163418667241
OGN	-0.0106396130522906
SLC47A1	-0.0913689486076044
RPS13	0.0467940333738643
SVIL	-0.0464904265445392
SLC6A9	0.0415232090999721
SERPINA5	-0.001558281515445
ENPP1	0.028868924976311
ANGPT2	0.233886201712768
F12	0.0438461683396056
HSPA1B	0.050901938774983
SORBS3	-0.0307660793115048
LAMC1	-0.0418790461734597
YWHAZ	0.00193600419951264
PGM5	0.00263885787491853
PDLIM7	0.0778608286944869
CD44	-0.0289933902615637
FBLN1	-0.024763746226886
ACTN1	0.0014153497740771
FLNB	-0.0114465760331545
ADAM17	0.165357292832258
ACTB	0.0230418694292494
AURKA	0.00125006325875106
SLC51A	-0.0658431606969681
TPSAB1	-0.00705762376805719
MATN2	0.00347420988431705
CA9	0.0131307181893591
SLC4A8	0.0133013499329606
CTSS	-0.0277072870981432

CSRP1	-0.0117801288070637
HACD3	0.23284167665824
EPB41L5	0.157486312190897
LAMA3	-0.0392112718480622
CLCA2	0.0205812641142881
ABCC5	0.0410089124892488
GNA13	-0.00196488508275753
ZP2	0.0139849401297409
SLC16A5	-0.0148260736254143
SLC4A11	0.0133916986763158
FGB	0.0139867914524806
SLC16A10	0.140166159831981
SLC1A5	0.0366219855130566
TGFBI	0.0192032998928875
