# Supplementary figures and images for: AdhesionScore: A Prognostic Predictor of Breast Cancer Patients Based on a Cell Adhesion-Associated Gene Signature
Source: Cancers (Basel). 2025 Nov 21;17(23):3731. doi: 10.3390/cancers17233731 (PMC12691146; doi:10.3390/cancers17233731)

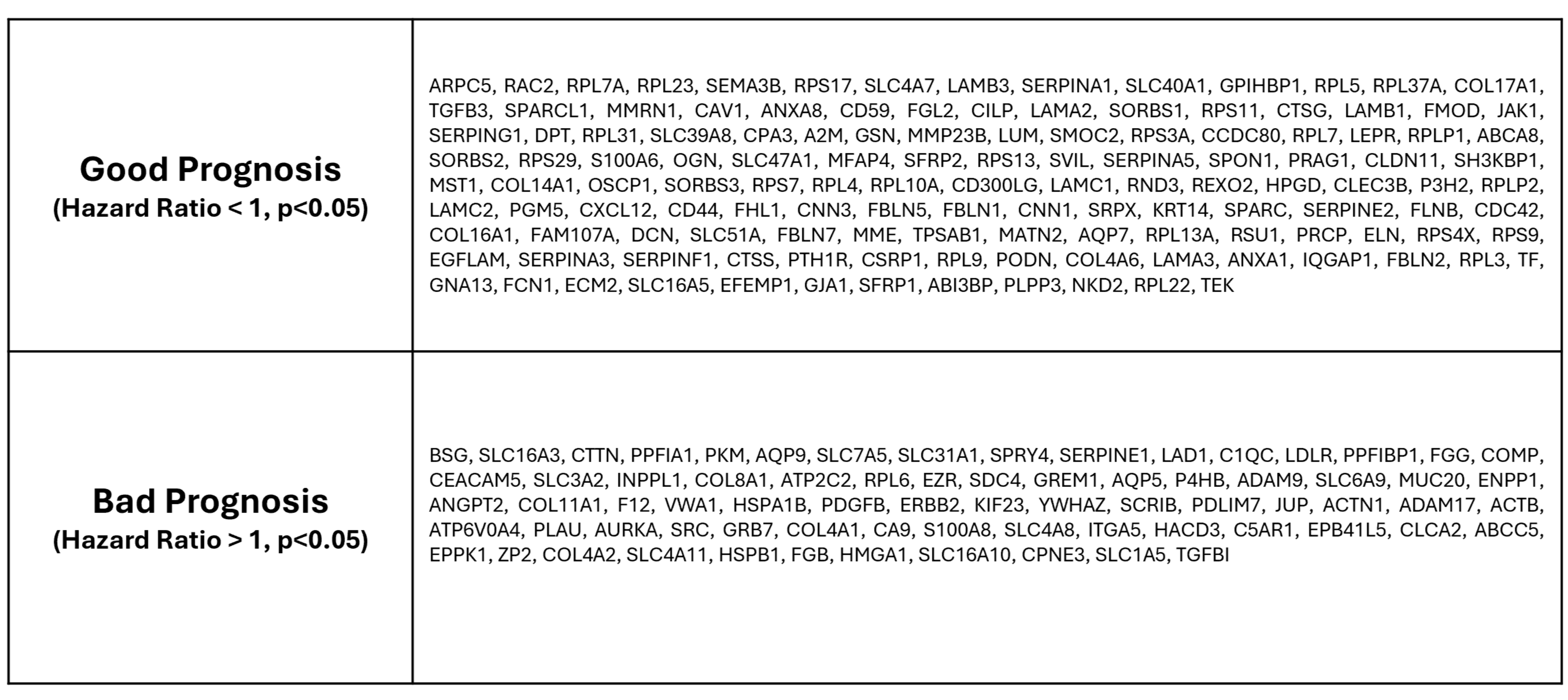

Supplement: Supplementary file 1 [file cancers-17-03731-s001.zip › FigSupp 1.png]

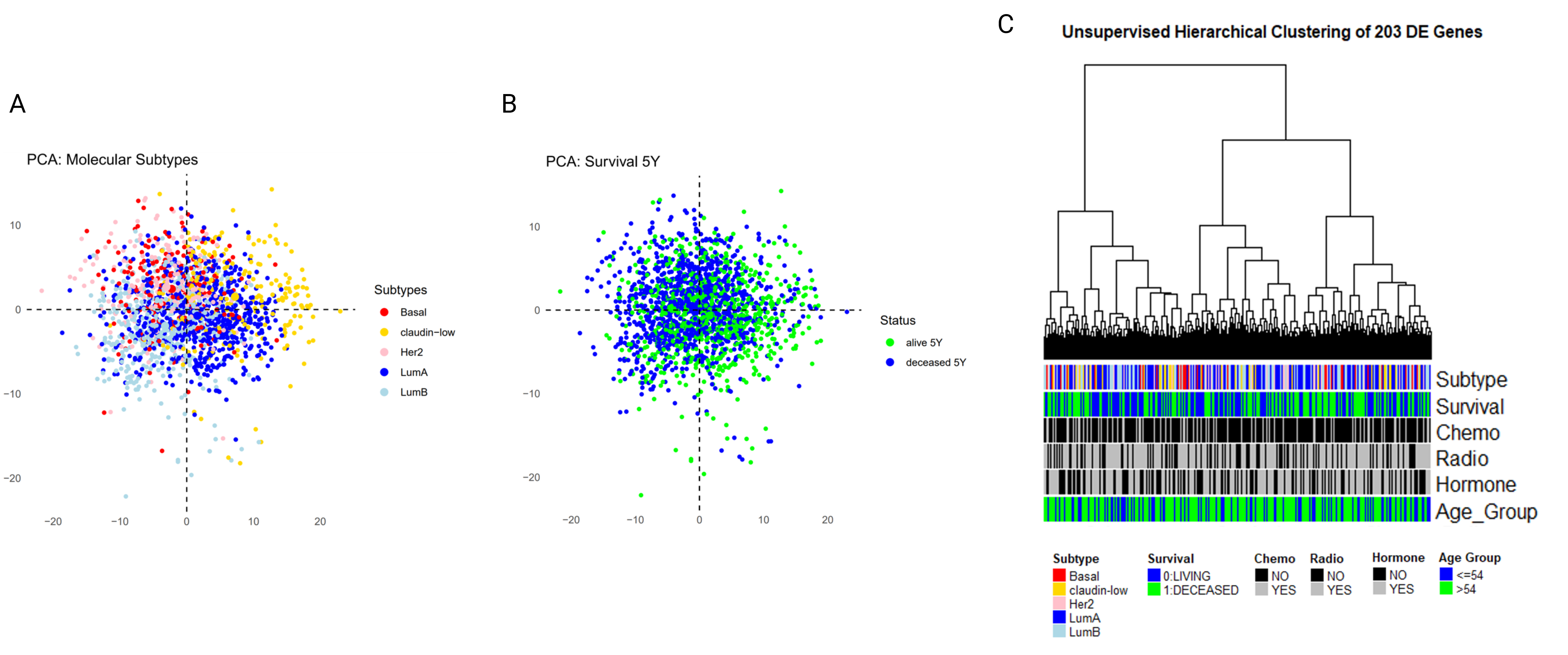

Supplement: Supplementary file 1 [file cancers-17-03731-s001.zip › FigSupp 2.png]

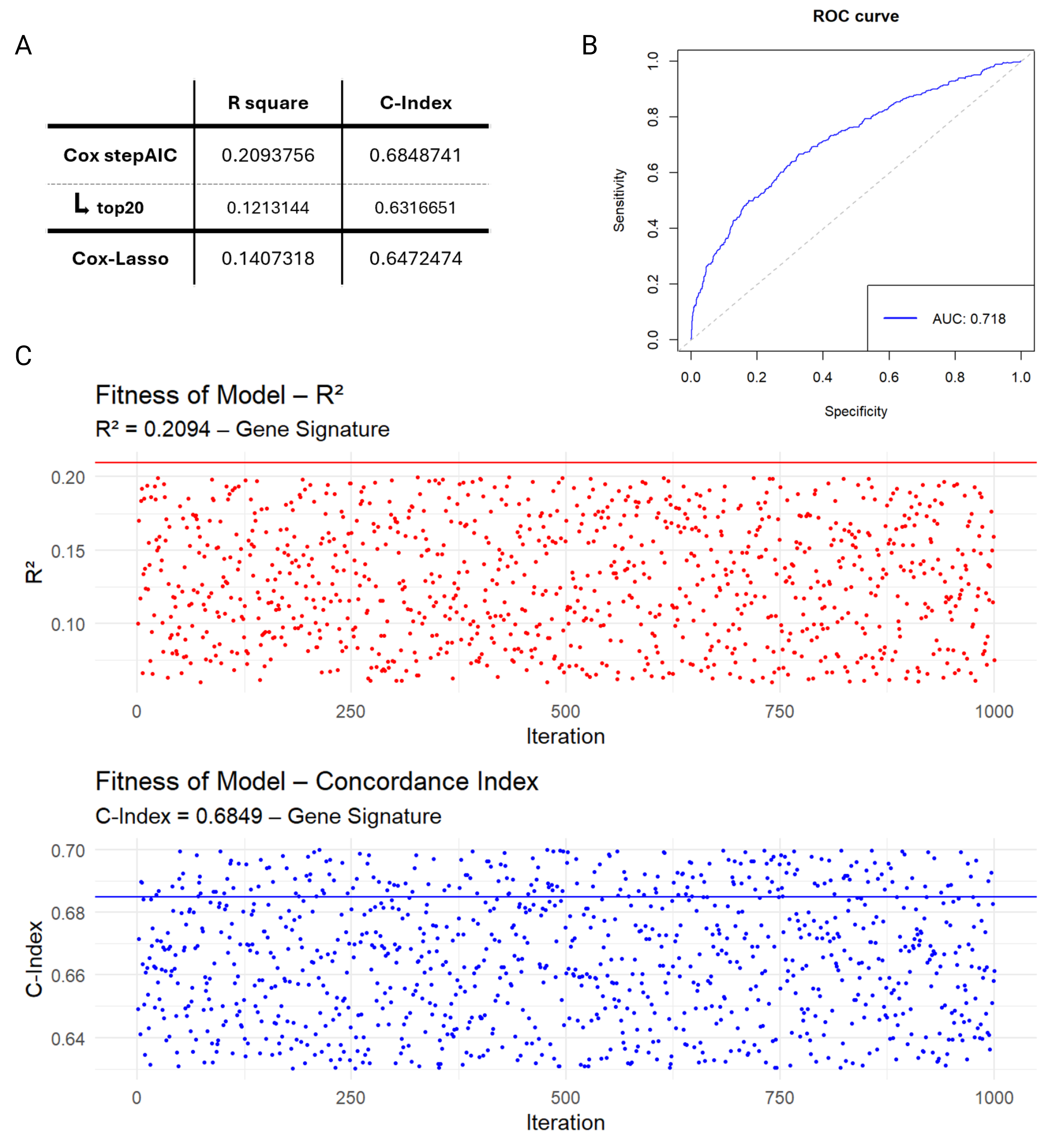

Supplement: Supplementary file 1 [file cancers-17-03731-s001.zip › FigSupp 3.png]

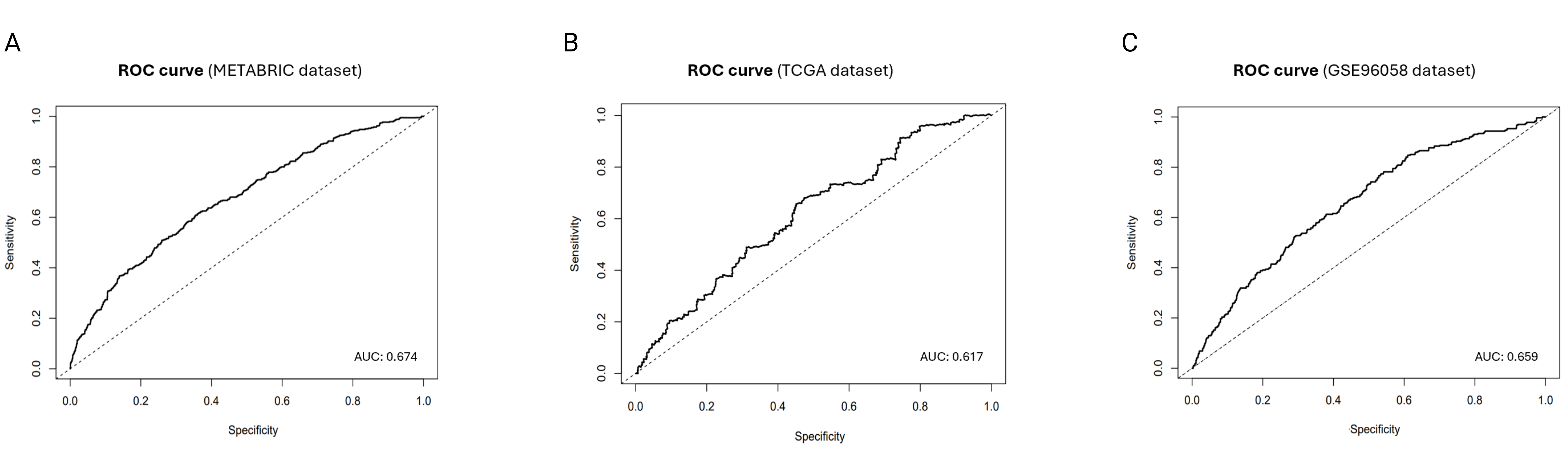

Supplement: Supplementary file 1 [file cancers-17-03731-s001.zip › FigSupp 4.png]
